# Expanding the Chemical Space of Drug-like Passerini Compounds: Can α-Acyloxy Carboxamides Be Considered Hard Drugs?

**DOI:** 10.1021/acsmedchemlett.2c00420

**Published:** 2022-11-03

**Authors:** Francesca Brunelli, Chiara Ceresa, Letizia Fracchia, Gian Cesare Tron, Silvio Aprile

**Affiliations:** Dipartimento di Scienze del Farmaco, Università degli Studi del Piemonte Orientale “A. Avogadro”, Largo Donegani 2, 28100 Novara, Italy

**Keywords:** Passerini reaction, α-acyloxy carboxamides, metabolism, prodrug, soft drug, hard drug

## Abstract

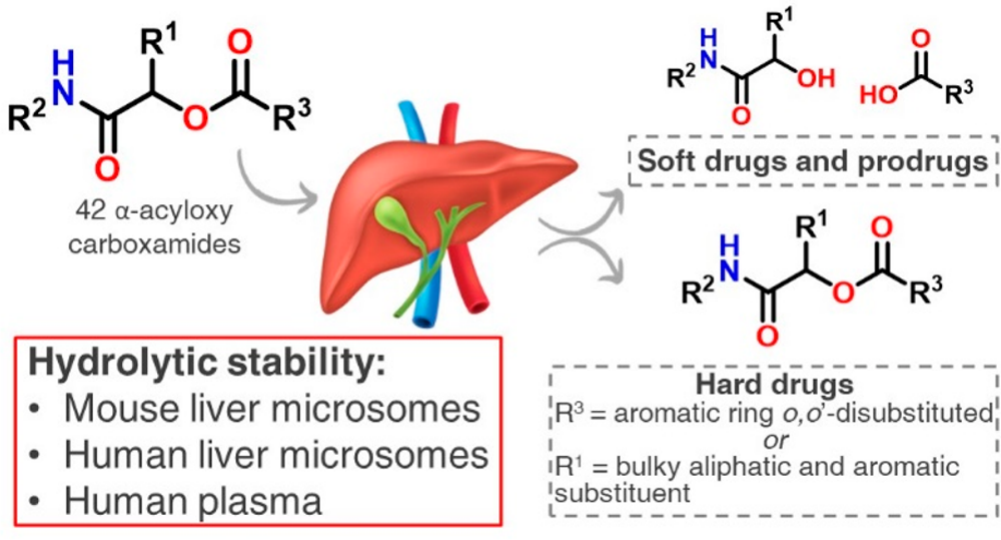

With their three points of diversity, α-acyloxy carboxamides, which are accessible with the Passerini reaction, provide heterogeneity for the preparation of libraries of putative active agents or intermediates used for the formation of more complex structures. If on the one hand the presence of a hydrolyzable ester function has been exploited to design both prodrugs and soft drugs, on the other hand medicinal chemists are reluctant to use this skeleton to prepare hard drugs. Herein we investigated whether the stability of the ester could be controlled, leading to the formation of hydrolytically stable α-acyloxy carboxamides. When the group directly attached to the ester moiety (R^3^) is an *ortho*-substituted or *ortho*,*ortho*′-disubstituted aromatic ring, α-acyloxy carboxamides are stable. In human liver but not in rodents, due to the different expression of esterases, the ester function is also stable toward hydrolysis when the R^1^ group is a bulky substituent regardless of the nature of the R^3^ substituent.

Discovered by serendipity in 1921 by Professor Mario Passerini in Florence,^1^ the eponymous transformation consists of a three-component reaction between an isocyanide, a carboxylic acid, and a carbonyl compound to give an α-acyloxy carboxamide. The very first Passerini product was obtained from the reaction between acetone (**1**), acetic acid (**2**), and *p*-isocyanoazobenzene (**3**) to form product **4** (Figure 1).^2^

**Figure 1:**
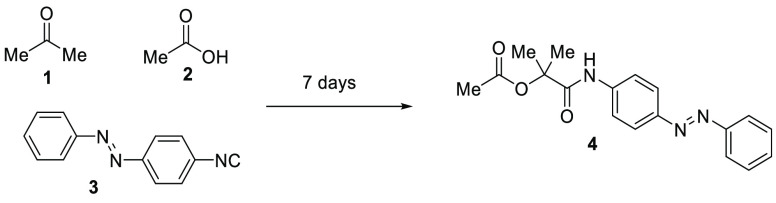
First Passerini product synthesized in 1921 in Florence.

The great merit of Passerini was to deduce the correct molecular formula of the unexpected product and to demonstrate the generality of this reaction in a series of papers published during the period 1921–1927.^3^ He also attempted to propose a possible reaction mechanism, which has been replaced by others over the years.^4^ Apart from some mechanistic works, the reaction fell into oblivion, and only during the 1980s did this transformation regain the interest it deserves thanks to the outburst of combinatorial chemistry and the use of multicomponent reactions.^5^

Although on its own the Passerini reaction appears to be mostly reproducible and tolerant to the presence of many functional groups, over the years novel methods have been reported to improve the yield (use of Lewis acids, microwave heating, ultrasound)^6^ and its environmental sustainability (reactions on water and/or in cationic vesicles),^7^ making this reaction even more attractive for the rapid preparation of libraries of compounds. The circle of sustainability was completed by the recent discovery that the use of either phosphorus-based chiral catalysts^8^ or (−)-4,5-dicarbonyl-3,6-bis(1-naphthyl)-1,2-benzenedisulfonimide supported on silica gel^9^ can give enantioselective syntheses of α-acyloxy carboxamides. With its three points of diversity, this reaction also fulfills the request of heterogeneity for the preparation of libraries of putative biologically active agents. To date, a search of the Sci-Finder database (accessed June 2022) found more than 300 000 α-acyloxy carboxamides, of which less than 4000 were synthesized via a one-pot Passerini reaction. Some of these compounds were synthetic intermediates used for the formation of more complex structures, while others were synthesized and employed as such to find out some biological activity. In detail, pharmacological studies were reported for 8352 α-acyloxy carboxamides. The biological activities of these molecules span different uses, with the antitumoral and the anti-infective activities being the most represented (Figure 2).^10−14^

**Figure 2:**
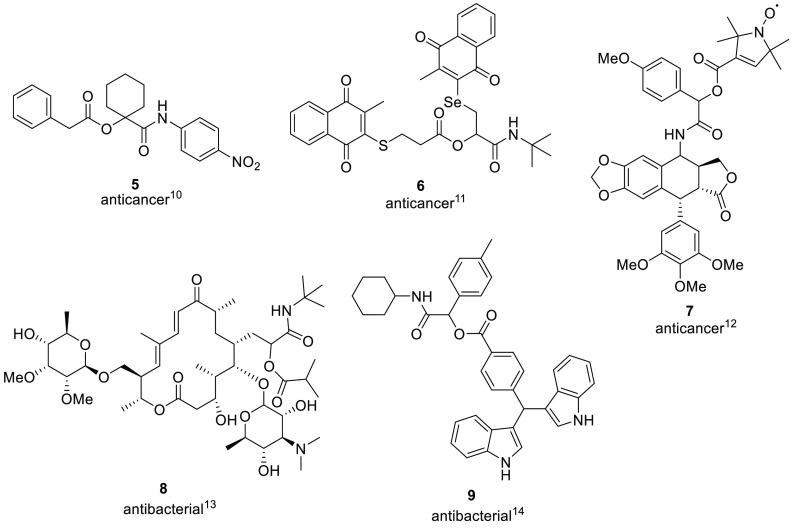
Examples of biologically active α-acyloxy carboxamides obtained with the Passerini reaction.

Despite these interesting facts, medicinal chemists are still reluctant to use the Passerini reaction to prepare putative drugs due to the presence of an ester group (Figure 3). Indeed, ester groups are usually labile functional groups in our body due to the presence of esterases. For this reason, some authors have exploited the Passerini reaction to design both prodrugs (e.g., NO-releasing NSAIDs)^15^ and soft drugs (e.g., a capsaicin agonist that is active only on the skin).^16^

**Figure 3:**
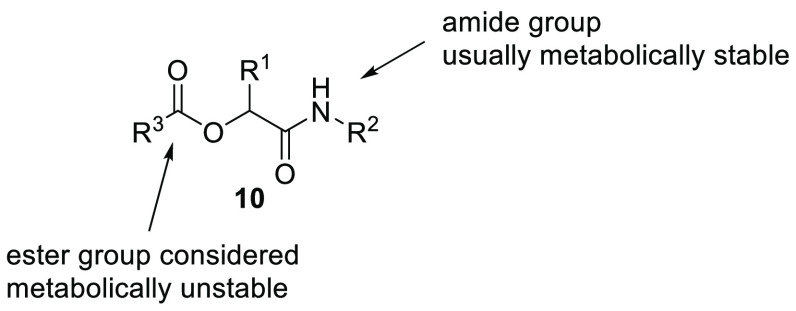
Structure of α-acyloxy carboxamides.

Esterases, which belong to the family of serine hydrolases, are almost ubiquitous enzymes that are found mainly in the liver but are also present in the skin, plasma, kidneys, brain, erythrocytes, and the gastrointestinal tract. The human liver is characterized by the presence of two isoforms of carboxylesterases (hCEs), with the hCE1 isoform more abundant than hCE2. In rodents, only CE1 is expressed in rat liver, whereas both isoforms are present in mice.^17^ The two isoforms hCE1 and hCE2 have orthogonal activities, with the former able to cleave esters that contain a small alcohol group and a bulky acyl group while the latter prefers to hydrolyze esters with a large alcohol group and a small acyl group.^18^ Although CEs are present in the plasma of most rodents, they are not found in the plasma of primates.^19^ Indeed, hydrolytic activity in human plasma is driven by butyrylcholinesterase (BChE), paraoxonase (PON1), and acetylcholinesterase (AChE). Furthermore, human serum albumin (HSA), despite its poor esterase activity, is so abundant that it can significantly contribute to ester hydrolysis.^20,21^

Apart from these distinctions, it has been shown how steric hindrance can reduce or even suppress the hydrolytic function of esterases, while the presence of electron-withdrawing groups on the acyl side can facilitate the enzymatic cleavage.^18^ To this point, recently we experimentally observed the different hydrolytic stabilities of methyl benzoate and methyl 2,6-dimethylbenzoate toward esterases. The former is fully hydrolyzed in liver microsomes, while the latter is completely resistant to esterases in human liver (Figure 4; see the Supporting Information).

**Figure 4:**
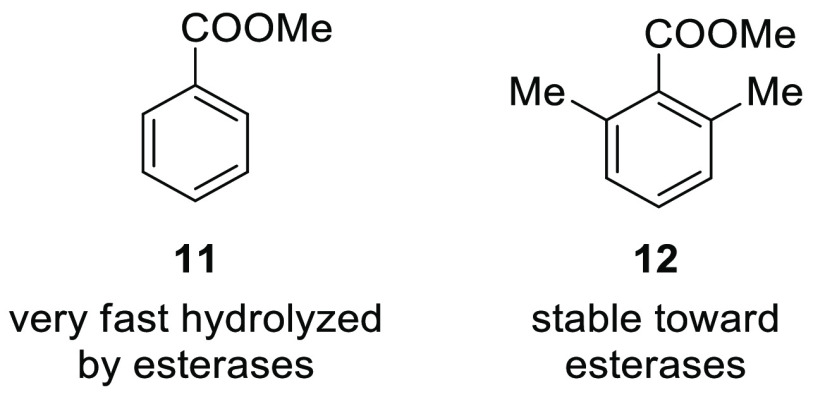
Hydrolytic stabilities of methyl benzoate and methyl 2,6-dimethylbenzoate toward esterases.

Driven by this simple observation, we wondered whether the stability of the ester in the skeleton of the Passerini product could be influenced by the other substructures present, leading to the formation of hydrolytically stable α-acyloxy carboxamides that would be useful in drug discovery as hard drugs.

To achieve this goal, we selected a library of nine aldehydes (**13**–**21**), two isocyanides (**22** and **23**), and 32 carboxylic acids (**24**–**55**) to form 43 α-acyloxy carboxamides (**56**–**98**) (Figure 5). In particular, we focused our attention on the carboxylic acids and aldehydes, choosing different substrate models that span from aliphatic to substituted aromatic compounds. We did not increase the complexity of the isocyanide-derived part of the molecule, as it is too far from the ester to alter its reactivity. All of the products were prepared using the classical Passerini conditions (DCM, rt, 0.5 M), purified by column chromatography, and fully characterized (^1^H, ^13^C, IR, HRMS, mp).

**Figure 5:**
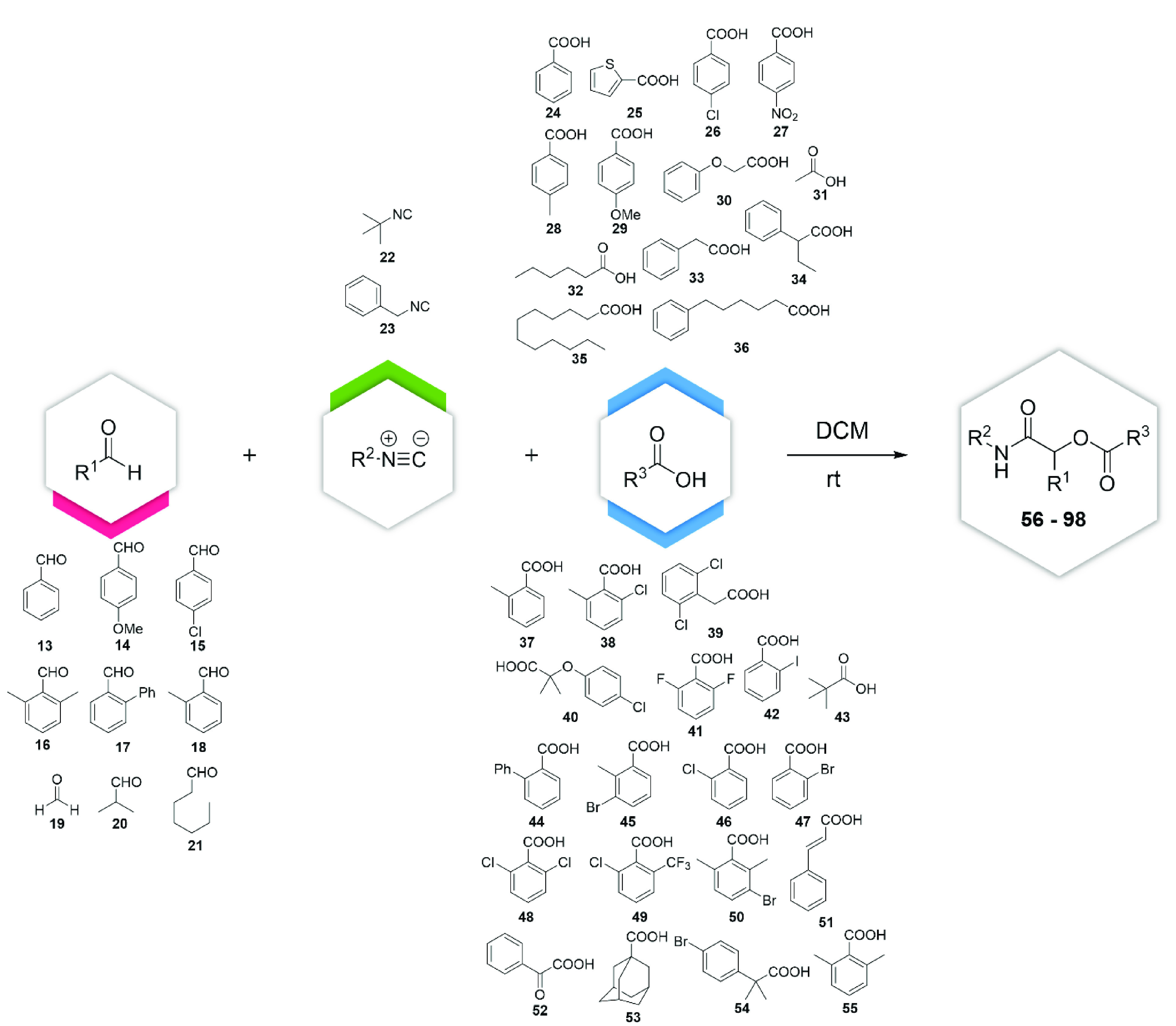
Building blocks used for this project.

For all 43 compounds, a first evaluation of their chemical and metabolic stabilities toward hydrolysis was performed by incubating them in buffer under physiological conditions (50 mM Tris-HCl, pH 7.4) and in mouse liver microsomes (Table 1). This model was chosen because, similar to the human liver, both of the carboxylesterase isoforms are present. The ester group was easily hydrolyzed when the molecules were derived from aliphatic acids (**70**, **71**, **73**, **75**, **77**) or benzylic acids, even when disubstituted at the α and α′ benzylic positions (**74**, **82**, **91**, **92**). The 1-carboxyladamantane product (**97**) was also rapidly metabolized. A more interesting trend was instead possible to observe with benzoic acids. Regardless of the natures of the aldehyde and isocyanide groups, with benzoic acid or thiophene-2-carboxylic acid (**56**–**64**, **72**) or with benzoic acids substituted at the *para* position (**66**–**69**), the ester group was rapidly cleaved by the esterases, whereas the behavior with benzoic acids substituted at the *ortho* position was completely different. With *ortho*-monosubstituted benzoic acids, the hydrolysis of the ester was complete (**81**, **86**, **87**, **89**, **90**), with the sole exception of the molecule obtained from 2-phenylbenzoic acid (**88**) (stability about 80%), and when the aromatic ring was *ortho*,*ortho*′-disubstituted (**78**–**80**, **93**–**96**, **98**), regardless of the electronic nature of the substituents, all of the α-acyloxy carboxamide Passerini products were found to be extremely stable toward metabolism of the ester group. It should be noted that when at the two *ortho* positions the isosteric replacement of H with F was attempted, the resulting compound **85** was completely hydrolyzed. The Passerini adducts derived from cinnamic acid (**65**), 2-oxo-2-phenylacetic acid (**83**), 2-phenoxyacetic acid (**76**), and its α,α′-dimethyl analogue (**84**) were again completely unstable toward metabolism.

**Table 1:**
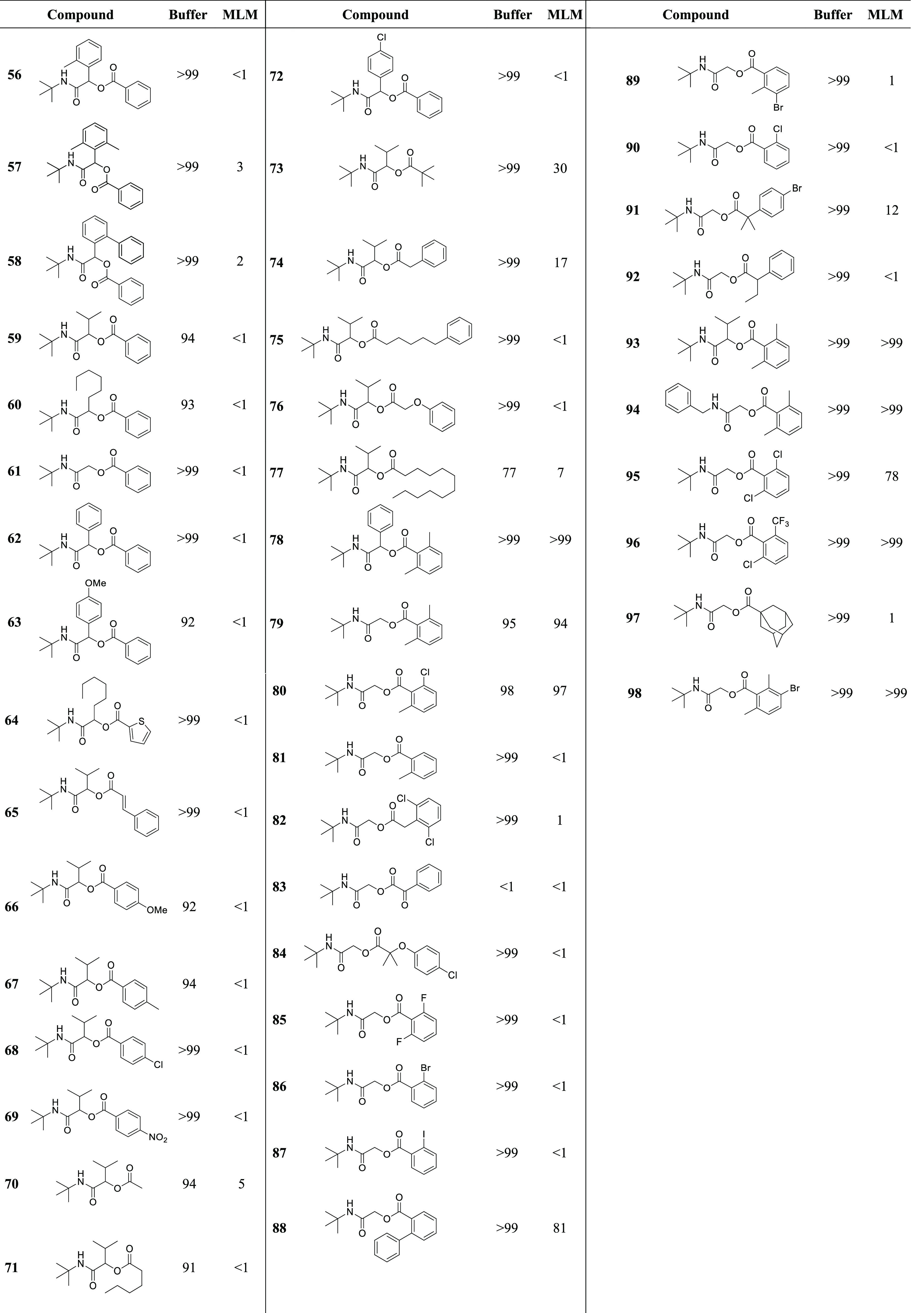
Hydrolytic Stabilities (Shown as Residual Substrate Percentages after Incubation for 30 min) of the Synthesized α-Acyloxy Carboxamides in Tris-HCl Buffer (pH 7.4) and in Mouse Liver Microsomes (MLM)

Then we tested some of the most stable α-acyloxy carboxamides (**78**, **80**, **98**) on human liver microsomes, along with some that had proved to be unstable in mouse (**57**, **58**, **64**, **65**, **73**, **84**, **89**, **97**) to compare the data (Table 2). This was made in light of the fact that the overall metabolizing capability of murine hepatic enzymes, including carboxylesterases, is supposed to be higher compared to the human ones.^17^ The comparison with the data offers interesting clues. The data on the excellent stability with *ortho*,*ortho*′-disubstituted benzoic acids (**78**, **80**, **98**) is confirmed, as well as the easy hydrolysis of the ester group for *ortho*-monosubstituted benzoic acids (**89**). The instability with other types of carboxylic acids is also confirmed (**64**, **65**, **84**, **97**), except when sterically hindered aldehydes are used. In this case the products formed using *ortho*-substituted benzaldeydes (**57**, **58**) appear to be stable to human liver microsomes in comparison to the murine model, in which they were rapidly degraded (for **57**, 73% vs 3% residual substrate; for **58**, >99% vs 2%). This result is consistent with the lower expression of hCE2 in the human liver compared to the mouse. hCE2 is indeed mainly involved in the hydrolysis of esters with large alcoholic groups. Finally, the compound containing pivalic acid (**73**) is moderately more stable in HLM (52% vs 30%)

**Table 2:** Hydrolytic Stabilities (Shown as Residual Substrate Percentages after Incubation for 30 min) of Some Synthesized α-Acyloxy Carboxamides in Human Liver Microsomes (HLM) and Human Plasma

compound	HLM	plasma
**57**	73	90
**58**	>99	90
**64**	<1	nd^a^
**65**	3	nd
**73**	52	nd
**78**	>99	>99
**80**	>99	>99
**84**	2	nd
**89**	<1	nd
**97**	2	nd
**98**	>99	>99

a nd: not determined.

As a final point, for some of the compounds characterized by the best hepatic stability, we also evaluated them in human plasma (Table 2) and under acidic and alkaline conditions (see the Supporting Information), the latter to ascertain the stability of α-acyloxy carboxamides in the gastrointestinal tract. All of the tested compounds showed excellent stability, suggesting that no hydrolytic activity different from that of carboxylesterases is involved in α-acyloxy carboxamide degradation. Once again, the *ortho*,*ortho*′-disubstitution of the aromatic ring is the one that gives the best metabolic stability.

After having demonstrated that it is possible to obtain metabolically stable α-acyloxy carboxamides that are potentially useful in medicinal chemistry, we tried to apply this strategy in a real case. Recently an article presented the synthesis of 1,3-phenylenebis(2-(cyclohexylamino)-2-oxoethane-1,1-diyl) dibenzoate (**99**) (Figure 6), obtained with a double Passerini reaction starting from 1,3-benzenedicarbaldehyde. It has been reported that this compound (as a mixture of two enantiomers and a *meso* form) were characterized by good antibacterial activity against the Gram-negative bacteria *Pseudomonas aeruginosa*.^22^ According to our studies, compounds **99** is expected to suffer from metabolic instability, whereas the new analogue **100** designed by us (Figure 6), containing methyl groups at the two *ortho* positions in each benzoate group, should be metabolically stable. We then synthesized the two compounds and evaluated their antibacterial activities on *P. aeruginosa* and their hydrolytic stabilities. The *in vitro* antibacterial activities of **99** and **100** were evaluated against the *P. aeruginosa* ATCC 9027 strain. In general, both of the compounds were able to inhibit the growth of the strain, showing moderate antibacterial activities with inhibition zones of 10.2 ± 1.2 mm (**99**) and 10.4 ± 1.3 mm (**100**) compared to gentamycin (22.3 ± 1.7 mm). No inhibition halos were observed around the wells containing DMSO alone.

**Figure 6:**
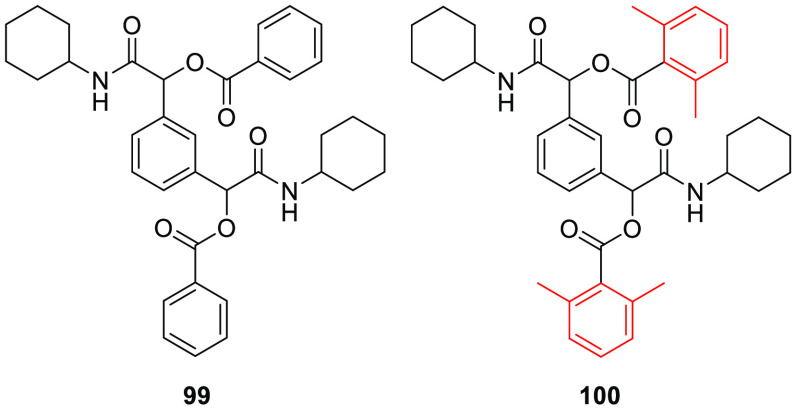
1,1-(Aryl)bis(2-(cyclohexylamino)-2-oxoethane-1,1-diyl) dibenzoates endowed with antibacterial activity against *Pseudomonas aeruginosa*.

Compound **99** showed high instability in mouse liver microsomes (Table 3 and Figure 7), whereas its hydrolytic stability increased in human liver microsomes. This is consistent with what we observed for compounds **57** and **58**, confirming the fact that α-acyloxy carboxamides bearing a large alcoholic group at the ester function have increased metabolic stability toward human esterases. To our pleasure, compound **100** was completely stable in both mouse and human liver, confirming that the presence of *ortho*,*ortho*′-disubstitution on the acyl phenyl ring fully stabilizes the α-acyloxy carboxamides toward hydrolysis in human liver.

**Table 3:** Hydrolytic Stabilities (Shown as Residual Substrate Percentages after Incubation for 30 min) of 1,1-(Aryl)bis(2-(cyclohexylamino)-2-oxoethane-1,1-diyl) Dibenzoates (**99**, **100**) in Mouse and Human Liver Microsomes and Human Plasma

compound	buffer	MLM	HLM	plasma
**99**	>99	5	47	93
**100**	>99	95	>99	84

**Figure 7:**
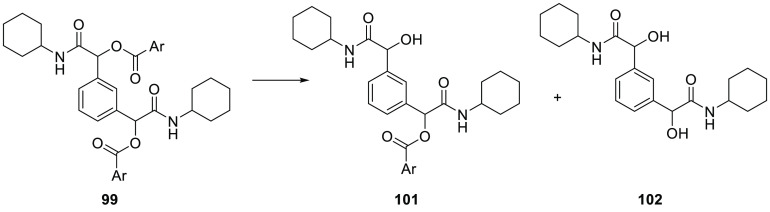
Hydrolytic metabolism of 1,3-phenylenebis(2-(cyclohexylamino)-2-oxoethane-1,1-diyl) dibenzoate (**99**).

In conclusion, the ester moiety present in the α-acyloxy carboxamide is usually considered a labile functional group due to the presence of hydrolytic enzymes widespread in the human body. For this reason, in the field of medicinal chemistry, the Passerini reaction has received scant attention because the ester group was always seen as a soft spot. In this work we demonstrated for the first time that it is possible to obtain metabolically stable α-acyloxy carboxamides that are potentially useful in medicinal chemistry. Our findings confirm that α-acyloxy carboxamides in which the group directly attached to the ester moiety is an *ortho*,*ortho*′-disubstituted aromatic ring have complete hydrolytic stability independent of the chemical nature of the R group of the aldehyde. On the other hand, when the R group of the aldehyde is a bulky substituent, we observed increased hydrolytic stability only in human microsomes, even with unsubstituted aromatic rings. The final aim of this work is therefore to raise awareness of medicinal chemists about the use of α-acyloxy carboxamides prepared via the Passerini reaction. Indeed, as demonstrated in the example of the antibacterial activity of 1,1-(aryl)bis(2-(cyclohexylamino)-2-oxoethane-1,1-diyl) dibenzoates, the judicious use of substituents on the acid and carbonyl components can give rise to either hard drugs or soft and prodrugs.

## Supporting Information Available

Experimental procedures, LC-HRMS methods and data, hydrolytic stability of compounds **11** and **12**, compound characterization data, copies of ^1^H and ^13^C spectra, and purities of selected compounds (PDF)

## Abbreviations

hCE: human carboxyl esterase
LC-HRMS: liquid chromatography coupled to high-resolution mass spectrometry
HLM: human liver microsomes
MLM: mouse liver microsomes
Tris-HCl: tris(hydroxymethyl)aminomethane hydrochloride
