# Supplementary material for: Expanding the Chemical Space of Drug-like Passerini Compounds: Can α-Acyloxy Carboxamides Be Considered Hard Drugs?
Source: ACS Med Chem Lett. 2022 Nov 3;13(12):1898–904. doi: 10.1021/acsmedchemlett.2c00420 (PMC9743426; doi:10.1021/acsmedchemlett.2c00420)
Supplement: Supplementary file 1 — ml2c00420_si_001.pdf [file ml2c00420_si_001.pdf]

Expanding the chemical space of drug-like Passerini compounds: can α-acyloxy carboxamides be considered hard drugs?

Francesca Brunelli,[a] Chiara Ceresa,[a] Letizia Fracchia,[a] Gian Cesare Tron[a] and Silvio Aprile*[a]

a) *Department of Pharmaceutical Sciences, Università degli Studi del Piemonte Orientale "A. Avogadro"*, Largo Donegani 2, 28100 Novara, Italy.

Contents

Experimental procedures S2
General procedure for the synthesis of compounds **56-60**, **62-78**, **93**....................S3
General procedure for the synthesis of compounds **61**, **79-92**, 9**4-98**....................S3
General procedure for the synthesis of compounds **99-100**....................S3
Hydrolytic stability....................S3
Hydrolytic stability in MLM and HLM....................S4
Hydrolytic stability in human plasma....................S4
Hydrolytic stability in acidic and alkaline conditions....................S4
Antibacterial activity....................S5
LC-HRMS methods and data....................S6
Hydrolytic stability of compounds **11** and **12**....................S9
Compounds characterization data....................S10
Copies of ^{1}H and ^{13}C spectra....................S18
Purity of selected compounds....................S62

Experimental procedures

Solvents and Reagents. Commercially available reagents and solvents were used without further purification.

Chromatography. Flash column chromatography was performed using Biotage Isolera One on silica gel 60 (Merck Kieselgel 230-400 mesh ASTM) using the indicated eluents. Thin layer chromatography (TLC) was carried out on 5 x 20 cm plates with a layer thickness of 0.25 mm (Merck Silica gel 60 F254). When necessary they were developed with $KMnO_4$.

Spectra. Infrared spectra were recorded on a FT-IR Bruker Alpha II spectrometer with absorption maxima (ν_{max}) recorded in wavenumbers (cm^{-1}). NMR spectra were recorded using a Bruker Avance Neo 400 MHz spectrometer. Chemical shifts (δ) are quoted in parts per million referenced to the residual solvent peak. The multiplicity of each signal is designated using the following abbreviations: s, singlet; d, doublet; t, triplet; p, quintet; m, multiplet; br s, broad singlet; dd, doublet of doublets; dt, doublet of triplets; td, triplet of doublets; heptd, heptet of doublets. Coupling constants (J) are reported in Hertz (Hz). High-resolution ESI-MS spectra were performed on a Hybrid quadrupole-orbitrap, Thermo Scientific Q-exactive Plus mass spectrometer. The spectra were recorded by infusion into the ESI source using methanol as the solvent.

Melting points. Melting points were determined in open glass capillary with a Buchi melting point M-560.

No unexpected or unusually high safety hazards were encountered

General procedure for the synthesis of compounds 56-60, 62-78, 93

The carboxylic acid derivative (0.5 mmol, 1 equiv.) was dissolved in dichloromethane (1 mL). The aldehyde (1 equiv.) and the isocyanide (1 equiv.) were added. The reaction mixture was stirred at 40°C for 2 days. Then, the mixture was washed with saturated hydrogen carbonate, brine and dried over sodium sulfate. After concentration under reduced pressure, the crude material was purified by flash column chromatography using the indicated eluents.

General procedure for the synthesis of compounds 61, 79-92, 94-98

The carboxylic acid derivative (0.5 mmol, 1 equiv.) was dissolved in dichloromethane (1 mL). 37% aqueous formaldehyde solution (4 equiv.) and the isocyanide (1 equiv.) were added. The reaction mixture was stirred at 40°C for 2 days. Then, the mixture was washed with saturated hydrogen carbonate, brine and dried over sodium sulfate. After concentration under reduced pressure, the crude material was purified by flash column chromatography using the indicated eluents.

General procedure for the synthesis of compounds 99-100

The compounds were synthesized according to literature procedure.[22] Compounds **99** was *crystallized* form ethanol. Compound **100** was purified dissolving it in DCM and then adding the resulting solution to petroleum ether; a white precipitate was formed. Spectra of compound **99** corresponded to those reported in literature.

Hydrolytic stability

Mouse liver microsomes (MLM), (protein concentration: 20 mg/mL, total CYP: 650 pmol/mg protein), human liver microsomes (HLM), (pooled mixed sex, fifty individual donors, protein concentration: 20 mg/mL, total CYP: 360 pmol/mg protein) were purchased from Corning B.V. Life Sciences (Amsterdam, The Netherlands). Human plasma: human recovered plasma pooled (088SER-PLP-K3EDTA) was purchase from Tebu-bio (Magenta, Italy).

Hydrolytic stability in MLM and HLM

The standard incubation mixture (200 µL final volume) was carried out in a 50 mM TRIS-HCl buffer (pH 7.4) containing 150 mM KCl, tween 80 as cosolvent (0.8 mg/mg protein), and the substrate (10 µM). After pre-equilibration of the mixture, an appropriate volume of MLM or HLM suspension was added to give a final protein concentration of 1.0 mg/mL. The mixture was shaken for 30 minutes at 37 °C. Control incubations were carried out without the presence or microsomes or in pure acetonitrile. Each incubation was stopped by the addition of 200 µL ice-cold acetonitrile, vortexed and centrifuged at 13000 rpm for 5 min. The supernatants were analyzed by LC-HRMS.

Hydrolytic stability in human plasma

The standard incubation mixture (100 µL final volume) was carried out by dissolving the tested substrate compounds (10 µM) in DMSO (5% final volume) in preincubated plasma at 37 °C. The mixture was shaken for 30 min at 37 °C. Control incubations were carried out without substrate. Each incubation was stopped by addition of 200 µL of ice-cold acetonitrile, vortexed, and centrifuged at 13000 rpm for 10 min. The supernatants were analyzed by LC-HRMS.

Hydrolytic stability in acidic and alkaline conditions

The hydrolytic stability of compounds **58** and **79** was evaluated in acidic and alkaline conditions according to the "recommendations on dissolution testing" European Pharmacopoeia 11.0 monograph: pH=1.2 in HCl/NaCl solution; pH=8.0 in potassium dihydrogen phosphate buffer. The tested compounds were dissolved in the aqueous media at the concentration of 100 µg/mL by adding the minimum amount of DMSO to obtain clear solutions. Samples were immediately analyzed by HPLC (see S62 for methods) and the analytes peak area measured. To check for stability, the same samples were stored in autosampler at room temperature overnight and then reanalyzed.

Antibacterial activity

The *in vitro* antibacterial activity of the compounds was assayed against the Gram-negative P. aeruginosa ATCC 9027 by the agar well-diffusion method. Compounds **99**, **100**, and gentamycin were dissolved in DMSO (Scharlab, Barcelona, ES) and tested at the concentration of 1 mg/mL. For comparison, gentamycin was used as a negative control of growth and DMSO as positive control of growth. The strain, obtained from the American Type Culture Collection (ATCC, Manassas, VA, United States) and stored at −80 °C in Brain Heart Infusion (Scharlab, Barcelona, ES) with 25% glycerol (Scharlab, Barcelona, ES), was cultured onto Tryptic Soy Agar (Scharlab, Barcelona, ES) at 37°C for 16 h.

A bacterial suspension (ca. 1.5×108 Colony Forming Unit per ml) was prepared in isotonic saline solution (NaCl 0,9%) and spread onto Muller-Hinton agar (Scharlab, Barcelona, ES) surfaces using a sterile swab. Wells (6 mm diameter) were punched using a sterile punch cutter and filled with 55 µL of each sample. Plates were left at room temperature for 1h to allow each sample to diffuse into the agar and then incubated at 37 °C for 24 h. Finally, the inhibition zone diameters were measured.

The assay was carried out in triplicate and repeated twice. Results were expressed as average values ± standard deviation.

LC-HRMS methods and data

Instrumentation:	Hybrid quadrupole-orbitrap, Thermo Scientific Q-exactive *Plus*, equipped with a Vanquish UHPLC system.

The operating conditions of the HESI were as follows:

Parameter	Value
sheath gas flow rate (N_2)	45 Auxiliary Units
auxiliary gas flow rate	5 Auxiliary Units
sweep gas flow rate	0 Auxiliary Units
spray voltage	3.20 kV
capillary temperature	300 °C
auxiliary gas heater temperature	280 °C

Column:	Kinetex C18 (100 × 2 mm, 2.6 µm d_p) (Phenomenex).
Column temperature:	40 °C
Sample temperature:	15 °C

Compounds for all the compounds except **11**

Mobile Phase	Phase A: 0.1% formic acid in water UHPLC grade.<br>Phase B: 0.1% formic acid in acetonitrile UHPLC grade.
Analysis mode:	Gradient of concentration.

Time (min)	% B
0.00	30
6.50	95
9.50	95
10.00	30
15.00	30

Flow rate:	0.300 mL/min
Injected volume:	5 µL

Compound **11**

Mobile Phase — Phase A: 0.1% formic acid in water UHPLC grade.

Phase B: methanol UHPLC grade.

Analysis mode: Gradient of concentration.

Time (min)	% B
0.00	30
4.00	40
6.50	95
9.50	95
10.00	30
15.00	30

Flow rate: 0.300 mL/min

Injected volume: 5 µL

Compound	Retention time (min)
**56**	5.58
**57**	6.04
**58**	6.32
**59**	4.96
**60**	6.51
**61**	3.60
**62**	5.14
**63**	5.08
**64**	6.30
**65**	5.43
**66**	5.01
**67**	5.43
**68**	5.62
**69**	5.00
**70**	3.03
**71**	5.65
**72**	5.74
**73**	5.15
**74**	5.06
**75**	6.54
**76**	4.92
**77**	8.30
**78**	5.76
**79**	4.48
**80**	4.64
**81**	4.24
**82**	4.77
**83**	3.99
**84**	5.30
**85**	3.85

**86**	4.28
**87**	4.48
**88**	5.12
**89**	5.07
**90**	4.20
**91**	5.54
**92**	4.92
**93**	5.58
**94**	5.43
**95**	4.59
**96**	4.83
**97**	5.63
**98**	5.28
**99**	6.43
**100**	7.23

LC-HRMS chromatograms of compound **99** and its metabolites originated from hydrolysis.

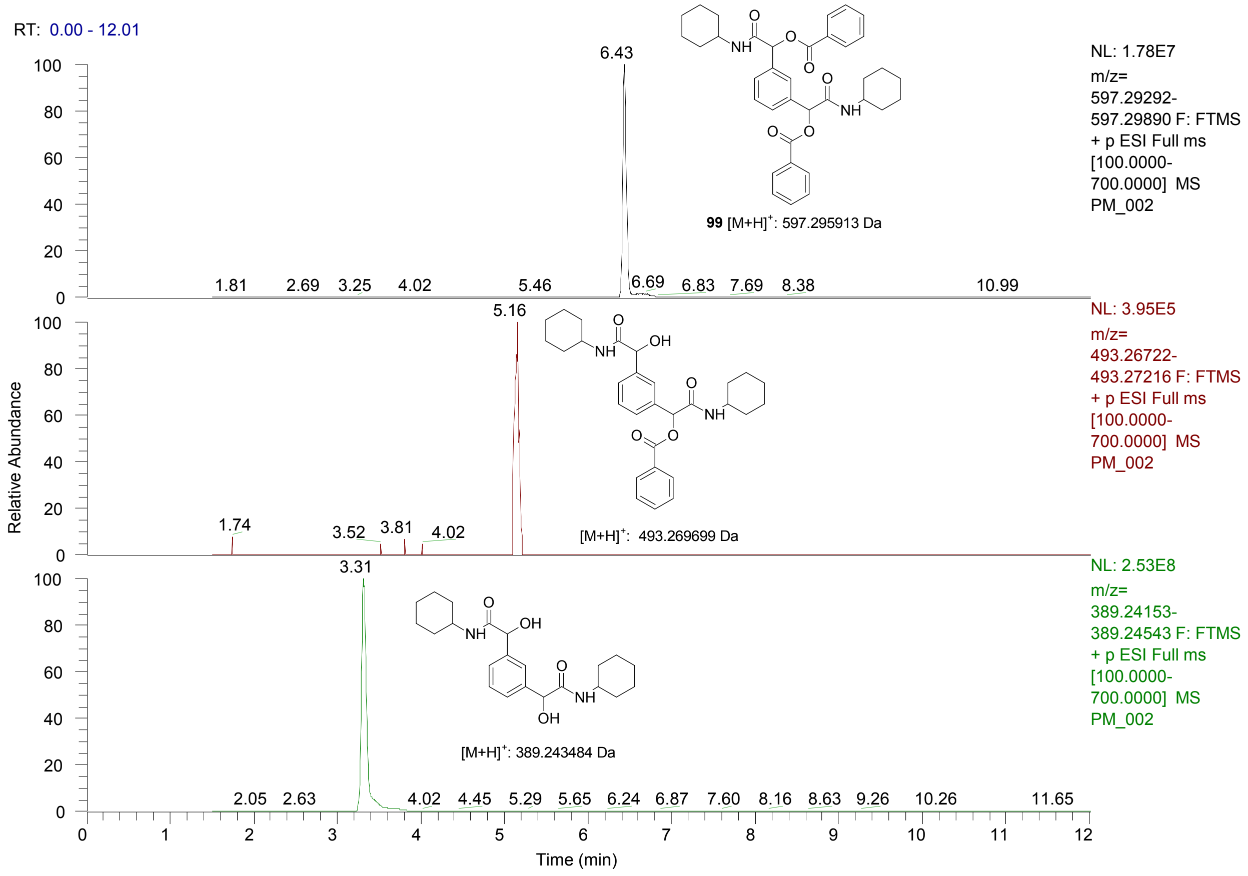

Hydrolytic stability of compounds 11 and 12

Compound	Buffer	MLM	HLM
**11**	>99	<1	<1
**12**	>99	77	>99

MLM: mouse liver microsomes

HLM: human liver microsomes

Residual percentage after 30 min incubation.

Compounds characterization data

2-(*tert*-butylamino)-2-oxo-1-(*o*-tolyl)ethyl benzoate 56
Eluent: Pet/EtOAc 7:3. White powder, yield 57 %, mp: 106.9-107.8 °C
^{1}H NMR (400 MHz, $CDCl_3$) δ = 8.13 – 8.07 (m, 2H), 7.64 – 7.59 (m, 1H), 7.53 – 7.44 (m, 3H), 7.31 – 7.21 (m, 3H), 6.49 (s, 1H), 6.03 (br s, 1H), 2.56 (s, 3H), 1.41 (s, 9H) ppm.
^{13}C NMR (101 MHz, $CDCl_3$) δ = 167.7, 165.0, 137.4, 134.6, 133.6, 131.0, 129.8, 129.4, 129.0, 128.7, 128.0, 126.3, 73.8, 51.6, 28.7, 19.6 ppm.
IR (neat) ν 3324, 1715, 1667, 1539, 1246, 1092, 1067, 765, 707 cm^{-1}
HRMS (ESI): *m/z*: calcd for $C_{20}H_{24}NO_3^+$ $[M+H]^+$: 326.17507, Found: 326.17462

2-(*tert*-butylamino)-1-(2,6-dimethylphenyl)-2-oxoethyl benzoate 57
Eluent: Pet/EtOAc 7:3. White powder, yield 42 % mp: 97.4-97.8 °C
^{1}H NMR (400 MHz, $CDCl_3$) δ = 8.10 (m, 2H), 7.61 (m, 1H), 7.49 (t, *J*=7.7 Hz, 2H), 7.16 (m, 1H), 7.07 (d, *J*=7.6 Hz, 2H), 6.87 (s, 1H), 5.88 (br s, 1H), 2.59 (s, 6H), 1.41 (s, 9H) ppm.
^{13}C NMR (101 MHz, $CDCl_3$) δ = 167.8, 165.0, 138.2, 133.5, 132.8, 129.8, 129.6, 129.1, 128.9, 128.7, 71.8, 51.7, 28.7, 20.4 ppm.
IR (neat) ν 3444, 1723, 1683, 1511, 1253, 1093, 714, 494 cm^{-1}
HRMS (ESI): *m/z*: calcd for $C_{21}H_{26}NO_3^+$ $[M+H]^+$: 340.19072, Found: 340.19077

1-([1,1'-biphenyl]-2-yl)-2-(*tert*-butylamino)-2-oxoethyl benzoate 58
Eluent: Pet/EtOAc 7:3. Colorless oil, yield 53 %
^{1}H NMR (400 MHz, $CDCl_3$) δ = 8.10 – 8.04 (m, 2H), 7.78 – 7.73 (m, 1H), 7.63 – 7.57 (m, 1H), 7.55 – 7.42 (m, 9H), 7.36 – 7.32 (m, 1H), 6.33 (s, 1H), 5.68 (br s, 1H), 1.34 (s, 9H) ppm.
^{13}C NMR (101 MHz, $CDCl_3$) δ = 167.5, 164.9, 142.2, 140.4, 133.8, 133.5, 130.4, 129.8, 129.7, 129.5, 128.7, 128.5, 128.5, 128.0, 127.6, 73.4, 51.5, 28.7 ppm.
IR (neat) ν 3437, 1723, 1683, 1513, 1254, 1092, 1068, 704 cm^{-1}
HRMS (ESI): *m/z*: calcd for $C_{25}H_{26}NO_3^+$ $[M+H]^+$: 388.19072, Found: 388.19064

1-(*tert*-butylamino)-3-methyl-1-oxobutan-2-yl benzoate 59
Eluent: Pet/EtOAc 9:1. White powder, yield 87 %, mp: 128.9-129.9 °C
^{1}H NMR (400 MHz, $CDCl_3$) δ = 8.12 – 8.07 (m, 2H), 7.67 – 7.61 (m, 1H), 7.55 – 7.48 (m, 2H), 5.86 (br s, 1H), 5.21 (d, *J*=4.2 Hz, 1H), 2.51 – 2.42 (m, 1H), 1.36 (s, 9H), 1.06 (d, *J*=6.8 Hz, 3H), 1.03 (d, *J*=6.9 Hz, 3H) ppm.
^{13}C NMR (101 MHz, $CDCl_3$) δ = 168.4, 165.4, 133.6, 129.6, 129.5, 128.7, 78.7, 51.3, 30.8, 28.7, 18.9, 16.9 ppm.
IR (neat) ν 3318, 1723, 1659, 1548, 1267, 1221, 1114, 708 cm^{-1}
HRMS (ESI): *m/z*: calcd for $C_{16}H_{24}NO_3^+$ $[M+H]^+$: 278.17507, Found: 278.17525

1-(*tert*-butylamino)-1-oxooctan-2-yl benzoate 60
Eluent: Pet/EtOAc 9:1. White powder, yield 83 %, mp: 96.9-97.8 °C
^{1}H NMR (400 MHz, $CDCl_3$) δ = 8.10 – 8.06 (m, 2H), 7.66 – 7.60 (m, 1H), 7.54 – 7.47 (m, 2H), 5.93 (br s, 1H), 5.32 (dd, *J*=6.5, 5.3, 1H), 2.02 – 1.94 (m, 2H), 1.44 – 1.27 (m, 17H), 0.90 – 0.84 (m, 3H) ppm.
^{13}C NMR (101 MHz, $CDCl_3$) δ = 169.0, 165.3, 133.5, 129.6, 129.6, 128.7, 74.8, 51.2, 31.9, 31.6, 29.0, 28.7, 24.8, 22.5, 14.0 ppm.
IR (neat) ν 3307, 1720, 1659, 1555, 1262, 1220, 1109, 1069, 714 cm^{-1}
HRMS (ESI): *m/z*: calcd for $C_{19}H_{30}NO_3^+$ $[M+H]^+$: 320.22202, Found: 320.22165

2-(*tert*-butylamino)-2-oxoethyl benzoate 61
Eluent: Pet/EtOAc 6:4. White powder, yield 90 %, mp: 113.8-114.5 °C
^{1}H NMR (400 MHz, $CDCl_3$) δ = 8.08 – 8.00 (m, 2H), 7.63 – 7.56 (m, 1H), 7.51 – 7.42 (m, 2H), 6.06 (br s, 1H), 4.69 (s, 2H), 1.38 (s, 9H) ppm.
^{13}C NMR (101 MHz, $CDCl_3$) δ = 166.2, 165.2, 133.6, 129.7, 129.1, 128.7, 63.6, 51.4, 28.7 ppm.
IR (neat) ν 3308, 1723, 1662, 1556, 1262, 1253, 1118, 704 cm^{-1}

HRMS (ESI): *m/z*: calcd for $C_{13}H_{18}NO_3^+$ $[M+H]^+$: 236.12812, Found: 236.12794

2-(*tert*-butylamino)-2-oxo-1-phenylethyl benzoate 62
Eluent: Pet/EtOAc 7:3. Yellow powder, yield 66 %, mp: 149.3-150.0 °C
^{1}H NMR (400 MHz, $CDCl_3$) δ = 8.12 (d, *J*=7.8 Hz, 2H), 7.63 – 7.54 (m, 3H), 7.48 (t, *J*=7.7 Hz, 2H), 7.45 – 7.35 (m, 3H), 6.27 (s, 1H), 6.14 (br s, 1H), 1.39 (s, 9H) ppm.
^{13}C NMR (101 MHz, $CDCl_3$) δ = 167.5, 165.0, 136.0, 133.6, 129.8, 129.4, 128.9, 128.8, 128.7, 127.5, 76.1, 51.6, 28.7 ppm.
IR (neat) ν 3289, 1721, 1653, 1556, 1265, 1115, 1069, 705 cm^{-1}
HRMS (ESI): *m/z*: calcd for $C_{19}H_{22}NO_3^+$ $[M+H]^+$: 312.15942, Found: 312.15891

2-(*tert*-butylamino)-1-(4-methoxyphenyl)-2-oxoethyl benzoate 63
Eluent: Pet/EtOAc 9:1. White powder, yield 39 %, mp: 144.9-145.9 °C
^{1}H NMR (400 MHz, $CDCl_3$) δ = 8.13 – 8.07 (m, 2H), 7.61 (t, *J*=7.3 Hz, 1H), 7.52 – 7.44 (m, 4H), 6.96 – 6.90 (m, 2H), 6.21 (s, 1H), 6.05 (br s, 1H), 3.81 (s, 3H), 1.39 (s, 9H) ppm.
^{13}C NMR (101 MHz, $CDCl_3$) δ = 167.7, 165.0, 160.1, 133.5, 129.7, 129.5, 129.1, 128.6, 128.1, 114.2, 75.8, 55.3, 51.6, 28.7 ppm.
IR (neat) ν 3291, 1722, 1656, 1514, 1265, 1249, 1116, 1038, 705, 540 cm^{-1}
HRMS (ESI): *m/z*: calcd for $C_{20}H_{23}NNaO_4^+$ $[M+Na]^+$: 364.15193, Found: 364.15140

1-(*tert*-butylamino)-1-oxooctan-2-yl thiophene-2-carboxylate 64
Eluent: Pet/EtOAc 9:1. White powder, yield 87 %, mp: 97.5-97.9 °C
^{1}H NMR (400 MHz, $CDCl_3$) δ = 7.88 (dd, *J*=3.8, 1.3 Hz, 1H), 7.64 (dd, *J*=5.0, 1.3 Hz, 1H), 7.17 (dd, *J*=5.0, 3.8 Hz, 1H), 5.97 (br s, 1H), 5.28 (dd, *J*=6.6, 5.0 Hz, 1H), 2.00 – 1.91 (m, 2H), 1.40 – 1.26 (m, 17H), 0.90 – 0.84 (m, 3H) ppm.
^{13}C NMR (101 MHz, $CDCl_3$) δ = 168.7, 160.7, 134.1, 133.0, 132.8, 128.2, 74.9, 51.3, 31.9, 31.6, 28.9, 28.7, 24.6, 22.5, 14.0 ppm.
IR (neat) ν 3303, 1709, 1658, 1558, 1257, 1226, 1097, 719 cm^{-1}
HRMS (ESI): *m/z*: calcd for $C_{17}H_{28}NO_3S^+$ $[M+H]^+$: 326.17844, Found: 326.17798

1-(*tert*-butylamino)-3-methyl-1-oxobutan-2-yl cinnamate 65
Eluent: Pet/EtOAc 8:2. White powder, yield 73 %, mp: 106.0-106.8 °C
^{1}H NMR (400 MHz, $CDCl_3$) δ = 7.76 (d, *J*=15.9 Hz, 1H), 7.58 – 7.51 (m, 2H), 7.42 – 7.37 (m, 3H), 6.51 (d, *J*=16.0 Hz, 1H), 5.88 (br s, 1H), 5.06 (d, *J*=4.5 Hz, 1H), 2.41 – 2.30 (m, 1H), 1.36 (s, 9H), 0.99 (d, *J*=4.6 Hz, 3H), 0.98 (d, *J*=4.2 Hz, 3H) ppm.
^{13}C NMR (101 MHz, $CDCl_3$) δ = 168.5, 165.8, 146.2, 134.0, 130.7, 129.0, 128.3, 117.0, 78.3, 51.3, 30.6, 28.7, 18.8, 17.0 ppm.
IR (neat) ν 3315, 1670, 1659, 1627, 1544, 1244, 1205, 1047, 993, cm^{-1}
HRMS (ESI): *m/z*: calcd for $C_{18}H_{26}NO_3^+$ $[M+H]^+$: 304.19072, Found: 304.19042

1-(*tert*-butylamino)-3-methyl-1-oxobutan-2-yl 4-methoxybenzoate 66
Eluent: Pet/EtOAc 8:2. White powder, yield 65 %, mp: 139.2-139.8 °C
^{1}H NMR (400 MHz, $CDCl_3$) δ = 8.09 – 8.01 (m, 2H), 7.02 – 6.94 (m, 2H), 5.86 (br s, 1H), 5.19 (d, *J*=4.2 Hz, 1H), 3.90 (s, 3H), 2.45 (m, 1H), 1.36 (s, 9H), 1.04 (d, *J*=6.8 Hz, 3H), 1.01 (d, *J*=6.9 Hz, 3H) ppm.
^{13}C NMR (101 MHz, $CDCl_3$) δ = 168.6, 165.1, 163.9, 131.7, 121.8, 114.0, 78.3, 55.5, 51.2, 30.7, 28.7, 19.0, 16.9 ppm.
IR (neat) ν 3297, 1714, 1657, 1554, 1230, 1250, 1171, 1103, 1032 cm^{-1}
HRMS (ESI): *m/z*: calcd for $C_{17}H_{26}NO_4^+$ $[M+H]^+$: 308.18563, Found: 308.18521

1-(*tert*-butylamino)-3-methyl-1-oxobutan-2-yl 4-methylbenzoate 67
Eluent: Pet/EtOAc 9:1. White powder, yield 86 %, mp: 120.5-121.0 °C
^{1}H NMR (400 MHz, $CDCl_3$) δ = 8.00 – 7.97 (m, 2H), 7.33 – 7.29 (m, 2H), 5.87 (br s, 1H), 5.22 (d, *J*=4.1 Hz, 1H), 2.50 – 2.41 (m, 4H), 1.36 (s, 9H), 1.05 (d, *J*=6.8 Hz, 3H), 1.02 (d, *J*=7.0 Hz, 3H) ppm.

^{13}C NMR (101 MHz, $CDCl_3$) δ = 168.5, 165.4, 144.4, 129.7, 129.4, 126.8, 78.4, 51.3, 30.7, 28.7, 21.7, 19.0, 16.9 ppm.
IR (neat) ν 3303, 1720, 1659, 1556, 1254, 1126, 1112, 753 cm^{-1}
HRMS (ESI): *m/z*: calcd for $C_{17}H_{26}NO_3^+$ $[M+H]^+$: 292.19072, Found: 292.19055

1-(*tert*-butylamino)-3-methyl-1-oxobutan-2-yl 4-chlorobenzoate 68
Eluent: Pet/EtOAc 8:2. White powder, yield 77 %, mp: 148.6-149.6 °C
^1H NMR (400 MHz, $CDCl_3$) δ (referred to the major rotamer) = 8.05 – 8.00 (m, 2H), 7.50 – 7.45 (m, 2H), 5.77 (br s, 1H), 5.14 (d, *J*=4.5 Hz, 1H), 2.51 – 2.37 (m, 1H), 1.36 (s, 9H), 1.05 (d, *J*=6.9 Hz, 3H), 1.02 (d, *J*=6.9 Hz, 3H) ppm.
^{13}C NMR (101 MHz, $CDCl_3$) δ (referred to the major rotamer) = 168.1, 164.6, 140.1, 131.0, 129.1, 128.0, 79.1, 51.4, 30.7, 28.7, 18.9, 17.0 ppm.
IR (neat) ν 3230, 1720, 1659, 1557, 1259, 1122, 1089, 765 cm^{-1}
HRMS (ESI): *m/z*: calcd for $C_{16}H_{23}ClNO_3^+$ $[M+H]^+$: 312.13610, Found: 312.13569

1-(*tert*-butylamino)-3-methyl-1-oxobutan-2-yl 4-nitrobenzoate 69
Eluent: Pet/EtOAc 9:1. White powder, yield 87 %, mp: 144.2-144.6 °C
^1H NMR (400 MHz, $CDCl_3$) δ = 8.36 – 8.32 (m, 2H), 8.28 – 8.24 (m, 2H), 5.71 (br s, 1H), 5.09 (d, *J*=5.0 Hz, 1H), 2.49 – 2.38 (m, 1H), 1.37 (s, 9H), 1.08 (d, *J*=6.8 Hz, 3H), 1.05 (d, *J*=6.9 Hz, 3H) ppm.
^{13}C NMR (101 MHz, $CDCl_3$) δ = 167.6, 163.8, 150.8, 134.9, 130.8, 123.8, 80.0, 51.6, 30.7, 28.7, 18.9, 17.3 ppm.
IR (neat) ν 3302, 1724, 1659, 1558, 1532, 1299, 1259, 1244, 1126, 718 cm^{-1}
HRMS (ESI): *m/z*: calcd for $C_{16}H_{23}N_2O_5^+$ $[M+H]^+$: 323.16015, Found: 323.15972

1-(*tert*-butylamino)-3-methyl-1-oxobutan-2-yl acetate 70
Eluent: Pet/EtOAc 8:2. White powder, yield 74 %, mp: 67.8-68.5 °C
^1H NMR (400 MHz, $CDCl_3$) δ = 5.76 (br s, 1H), 4.86 (d, *J*=4.6 Hz, 1H), 2.29 – 2.17 (m, 1H), 2.12 (s, 3H), 1.32 (s, 9H), 0.90 (d, *J*=7.0 Hz, 3H), 0.90 (d, *J*=6.8 Hz, 3H) ppm.
^{13}C NMR (101 MHz, $CDCl_3$) δ = 169.8, 168.4, 78.3, 51.2, 30.5, 28.6, 20.9, 18.6, 16.9 ppm.
IR (neat) ν 3294, 1735, 1657, 1551, 1365, 1239, 1221, 1036 cm^{-1}
HRMS (ESI): *m/z*: calcd for $C_{11}H_{22}NO_3^+$ $[M+H]^+$: 216.15942, Found: 216.15924

1-(*tert*-butylamino)-3-methyl-1-oxobutan-2-yl hexanoate 71
Eluent: Pet/EtOAc 9:1. White powder, yield 81 %, mp: 35.1-35.5 °C
^1H NMR (400 MHz, $CDCl_3$) δ = 5.76 (br s, 1H), 4.90 (d, *J*=4.5 Hz, 1H), 2.37 (t, *J*=7.5 Hz, 2H), 2.29 – 2.19 (m, 1H), 1.70 – 1.58 (m, 2H), 1.36 – 1.27 (m, 13H), 0.93 – 0.85 (m, 9H) ppm.
^{13}C NMR (101 MHz, $CDCl_3$) δ = 172.5, 168.5, 78.0, 51.2, 34.3, 31.2, 30.4, 28.6, 24.7, 22.2, 18.7, 16.8, 13.8 ppm.
IR (neat) ν 3312, 1745, 1658, 1556, 1227, 1171, 1119, 991 cm^{-1}
HRMS (ESI): *m/z*: calcd for $C_{15}H_{30}NO_3^+$ $[M+H]^+$: 272.22202, Found: 272.22187

2-(*tert*-butylamino)-1-(4-chlorophenyl)-2-oxoethyl benzoate 72
Eluent: Pet/EtOAc 6:4. White powder, yield 66%, mp: 193.8-194.8 °C
^1H NMR (400 MHz, $CDCl_3$) δ (referred to the major rotamer) = 8.14 – 8.07 (m, 2H), 7.67 – 7.60 (m, 1H), 7.55 – 7.45 (m, 4H), 7.42 – 7.36 (m, 2H), 6.21 (s, 1H), 6.09 (br s, 1H), 1.39 (s, 9H) ppm.
^{13}C NMR (101 MHz, $CDCl_3$) δ (referred to the major rotamer) = 167.0, 164.8, 134.9, 134.5, 133.8, 129.7, 129.1, 129.0, 128.8, 128.7, 75.3, 51.7, 28.7 ppm.
IR (neat) ν 3279, 1719, 1653, 1556, 1257, 1116, 1090, 704 cm^{-1}
HRMS (ESI): *m/z*: calcd for $C_{19}H_{21}ClNO_3^+$ $[M+H]^+$: 346.12045, Found: 346.12012

1-(*tert*-butylamino)-3-methyl-1-oxobutan-2-yl pivalate 73
Eluent: Pet/EtOAc 95:5. White powder, yield 76 %, mp: 61.4-62.3 °C

^1H NMR (400 MHz, $CDCl_3$) δ = 5.76 (br s, 1H), 4.97 (d, *J*=3.9 Hz, 1H), 2.38 – 2.27 (m, 1H), 1.33 (s, 9H), 1.27 (s, 9H), 0.92 (d, *J*=7.0 Hz, 3H), 0.92 (d, *J*=6.8 Hz, 3H) ppm.
^{13}C NMR (101 MHz, $CDCl_3$) δ = 176.7, 168.6, 77.5, 51.1, 38.9, 30.5, 28.6, 27.1, 18.9, 16.5 ppm.
IR (neat) ν 3334, 1740, 1662, 1538, 1217, 1157, 1131, 1008 cm^{-1}
HRMS (ESI): *m/z*: calcd for $C_{14}H_{28}NO_3^+$ $[M+H]^+$: 258.20637, Found: 258.20612

1-(*tert*-butylamino)-3-methyl-1-oxobutan-2-yl 2-phenylacetate 74
Eluent: Pet/EtOAc 8:2. White powder, yield 87 %, mp: 49.9-50.7 °C
^1H NMR (400 MHz, $CDCl_3$) δ = 7.38 – 7.26 (m, 5H), 5.41 (br s, 1H), 4.97 (d, *J*=4.0 Hz, 1H), 3.70 (s, 2H), 2.32 – 2.22 (m, 1H), 1.15 (s, 9H), 0.90 (d, *J*=7.0 Hz, 3H), 0.86 (d, *J*=6.8 Hz, 3H) ppm.
^{13}C NMR (101 MHz, $CDCl_3$) δ = 169.8, 168.2, 133.7, 129.2, 129.0, 127.5, 78.2, 51.0, 41.7, 30.5, 28.5, 18.7, 16.6 ppm.
IR (neat) ν 3314, 1726, 1654, 1551, 1251, 1147, 1020, 696 cm^{-1}
HRMS (ESI): *m/z*: calcd for $C_{17}H_{26}NO_3^+$ $[M+H]^+$: 292.19072, Found: 292.18996

1-(*tert*-butylamino)-3-methyl-1-oxobutan-2-yl 6-phenylhexanoate 75
Eluent: Pet/EtOAc 7:3. Colorless oil, yield 50 %
^1H NMR (400 MHz, $CDCl_3$) δ (referred to the major rotamer) = 7.32 – 7.26 (m, 2H), 7.22 – 7.16 (m, 3H), 5.77 (br s, 1H), 4.94 (d, *J*=4.5 Hz, 1H), 2.63 (t, *J*=7.6 Hz, 2H), 2.42 (t, *J*=7.5 Hz, 2H), 2.33 – 2.24 (m, 1H), 1.76 – 1.63 (m, 4H), 1.46 – 1.39 (m, 2H), 1.36 (s, 9H), 0.96 – 0.91 (m, 6H) ppm.
^{13}C NMR (101 MHz, $CDCl_3$) δ = 172.4, 168.5, 142.3, 128.4, 128.3, 125.8, 78.1, 51.2, 35.7, 34.3, 31.1, 30.5, 28.8, 28.7, 25.0, 18.8, 16.9 ppm.
IR (neat) ν 3307, 1738, 1664, 1519, 1224, 1179, 1124, 698 cm^{-1}
HRMS (ESI): *m/z*: calcd for $C_{21}H_{34}NO_3^+$ $[M+H]^+$: 348.25332, Found: 348.25253

1-(*tert*-butylamino)-3-methyl-1-oxobutan-2-yl 2-phenoxyacetate 76
Eluent: Pet/EtOAc 8:2. Colorless oil, yield 92%
^1H NMR (400 MHz, $CDCl_3$) δ = 7.34 – 7.27 (m, 2H), 7.04 – 6.98 (m, 1H), 6.94 – 6.89 (m, 2H), 5.76 (br s, 1H), 5.05 (d, *J*=4.4 Hz, 1H), 4.76 (d, *J*=16.2 Hz, 1H), 4.69 (d, *J*=16.2 Hz, 1H), 2.29 (heptd, *J*=6.9, 4.4 Hz, 1H), 1.27 (s, 9H), 0.92 (d, *J*=6.9 Hz, 3H), 0.89 (d, *J*=6.9 Hz, 3H) ppm.
^{13}C NMR (101 MHz, $CDCl_3$) δ = 168.1, 167.7, 157.7, 129.8, 122.0, 114.4, 79.0, 65.3, 51.3, 30.7, 28.5, 18.6, 16.8 ppm.
IR (neat) ν 3324, 1752, 1664, 1523, 1251, 1188, 1173, 1086, 752 cm^{-1}
HRMS (ESI): *m/z*: calcd for $C_{17}H_{26}NO_4^+$ $[M+H]^+$: 308.18563, Found: 308.18494

1-(*tert*-butylamino)-3-methyl-1-oxobutan-2-yl dodecanoate 77
Eluent: Pet/EtOAc 9:1. White powder, yield 71%, mp: 40.7-41.1 °C
^1H NMR (400 MHz, $CDCl_3$) δ = 5.76 (br s, 1H), 4.91 (d, *J*=4.5 Hz, 1H), 2.37 (t, *J*=7.5 Hz, 2H), 2.29 – 2.20 (m, 1H), 1.64 (p, *J*=7.2 Hz, 2H), 1.37 – 1.19 (m, 25H), 0.90 (d, *J*=7.0 Hz, 3H), 0.90 (d, *J*=6.8 Hz, 3H) 0.85 (t, *J*=6.7, 3H) ppm.
^{13}C NMR (101 MHz, $CDCl_3$) δ = 172.4, 168.5, 78.0, 51.1, 34.3, 31.8, 30.4, 29.5, 29.4, 29.3, 29.2, 29.1, 28.6, 25.1, 22.6, 18.7, 16.9, 14.0 ppm.
IR (neat) ν 3312, 1744, 1664, 1554, 1267, 1225, 1163, 1014 cm^{-1}
HRMS (ESI): *m/z*: calcd for $C_{21}H_{42}NO_3^+$ $[M+H]^+$: 356.31592, Found: 356.31573

2-(*tert*-butylamino)-2-oxo-1-phenylethyl 2,6-dimethylbenzoate 78
Eluent: Pet/EtOAc 8:2. White powder, yield 58%, mp: 126.2-127.1 °C
^1H NMR (400 MHz, $CDCl_3$) δ = 7.54 – 7.49 (m, 2H), 7.44 – 7.36 (m, 3H), 7.25 (t, *J*=7.6 Hz, 1H), 7.07 (d, *J*=7.7 Hz, 2H), 6.29 (s, 1H), 6.10 (br s, 1H), 2.29 (s, 6H), 1.40 (s, 9H) ppm.
^{13}C NMR (101 MHz, $CDCl_3$) δ = 168.2, 167.3, 135.8, 135.1, 132.9, 129.8, 129.0, 128.7, 127.7, 127.7, 76.3, 51.7, 28.7, 19.7 ppm.
IR (neat) ν 3326, 1724, 1657, 1545, 1247, 1111, 1072, 771, 730 cm^{-1}
HRMS (ESI): *m/z*: calcd for $C_{21}H_{26}NO_3^+$ $[M+H]^+$: 340.19072, Found: 340.19012

2-(*tert*-butylamino)-2-oxoethyl 2,6-dimethylbenzoate 79
Eluent: Pet/EtOAc 8:2. Colorless oil, yield 66%
^{1}H NMR (400 MHz, $CDCl_3$) δ = 7.24 (t, *J*=7.6 Hz, 1H), 7.08 (d, *J*=7.7 Hz, 2H), 6.02 (br s, 1H), 4.70 (s, 2H), 2.36 (s, 6H), 1.39 (s, 9H) ppm.
^{13}C NMR (101 MHz, $CDCl_3$) δ = 168.4, 165.9, 135.2, 132.7, 129.9, 127.8, 63.7, 51.5, 28.8, 19.9 ppm.
IR (neat) ν 3311, 1732, 1664, 1541, 1243, 1115, 1077, 772 cm^{-1}
HRMS (ESI): *m/z*: calcd for $C_{15}H_{22}NO_3^+$ $[M+H]^+$: 264.15942, Found: 264.15933

2-(*tert*-butylamino)-2-oxoethyl 2-chloro-6-methylbenzoate 80
Eluent: Pet/EtOAc 6:4. White powder, yield 63%, mp: 64.2-65.2 °C
^{1}H NMR (400 MHz, $CDCl_3$) δ (referred to the major rotamer) = 7.34 – 7.28 (m, 2H), 7.21 – 7.17 (m, 1H), 6.20 (br s, 1H), 4.76 (s, 2H), 2.37 (s, 3H), 1.40 (s, 9H) ppm.
^{13}C NMR (101 MHz, $CDCl_3$) δ (referred to the major rotamer) = 165.7, 165.6, 137.6, 132.5, 130.8, 130.4, 128.9, 126.9, 63.8, 51.6, 28.7, 19.7 ppm.
IR (neat) ν 3309, 1736, 1670, 1553, 1258, 1220, 1110, 782 cm^{-1}
HRMS (ESI): *m/z*: calcd for $C_{14}H_{19}ClNO_3^+$ $[M+H]^+$: 284.10480, Found: 284.10434

2-(*tert*-butylamino)-2-oxoethyl 2-methylbenzoate 81
Eluent: Pet/EtOAc 7:3. White powder, yield 74%, mp: 119.5-120.4 °C
^{1}H NMR (400 MHz, $CDCl_3$) δ = 7.93 (d, *J*=7.9 Hz, 1H), 7.46 (t, *J*=7.4 Hz, 1H), 7.34 – 7.24 (m, 2H), 6.03 (br s, 1H), 4.68 (s, 2H), 2.63 (s, 3H), 1.40 (s, 9H) ppm.
^{13}C NMR (101 MHz, $CDCl_3$) δ = 166.3, 165.9, 140.6, 132.6, 132.0, 130.5, 128.5, 126.0, 63.6, 51.4, 28.8, 21.8 ppm.
IR (neat) ν 3306, 1723, 1662, 1555, 1247, 1222, 1098, 737 cm^{-1}
HRMS (ESI): *m/z*: calcd for $C_{14}H_{20}NO_3^+$ $[M+H]^+$: 250.14377, Found: 250.14343

2-(*tert*-butylamino)-2-oxoethyl 2-(2,6-dichlorophenyl)acetate 82
Eluent: Pet/EtOAc 7:3. White powder, yield 97%, mp: 80.0-80.8 °C
^{1}H NMR (400 MHz, $CDCl_3$) δ = 7.39 (br d, 2H), 7.24 (m, 1H), 5.76 (br s, 1H), 4.51 (s, 2H), 4.14 (s, 2H), 1.33 (s, 9H) ppm.
^{13}C NMR (101 MHz, $CDCl_3$) δ = 167.5, 165.7, 135.9, 130.6, 129.4, 128.3, 63.7, 51.4, 36.8, 28.7 ppm.
IR (neat) ν 3271, 1749, 1660, 1562, 1214, 1161, 933, 777 cm^{-1}
HRMS (ESI): *m/z*: calcd for $C_{14}H_{18}Cl_2NO_3^+$ $[M+H]^+$: 318.06583, Found: 318.06555

2-(*tert*-butylamino)-2-oxoethyl 2-oxo-2-phenylacetate 83
Eluent: Pet/EtOAc 7:3. Colorless oil, yield 59%
^{1}H NMR (400 MHz, $CDCl_3$) δ = 8.11 – 8.06 (m, 2H), 7.74 – 7.67 (m, 1H), 7.59 – 7.51 (m, 2H), 6.12 (br s, 1H), 4.74 (s, 2H), 1.40 (s, 9H) ppm.
^{13}C NMR (101 MHz, $CDCl_3$) δ (referred to the major rotamer) = 185.3, 165.0, 161.9, 135.5, 132.0, 130.3, 129.1, 63.6, 51.8, 28.7 ppm.
IR (neat) ν 3309, 1745, 1681, 1538, 1192, 1172, 1043, 680 cm^{-1}
HRMS (ESI): *m/z*: calcd for $C_{14}H_{18}NO_4^+$ $[M+H]^+$: 264.12303, Found: 264.12266

2-(*tert*-butylamino)-2-oxoethyl 2-(4-chlorophenoxy)-2-methylpropanoate 84
Eluent: Pet/EtOAc 8:2. Colorless oil, yield 39%
^{1}H NMR (400 MHz, $CDCl_3$) δ = 7.27 – 7.20 (m, 2H), 6.82 – 6.75 (m, 2H), 5.69 (br s, 1H), 4.54 (s, 2H), 1.64 (s, 6H), 1.22 (s, 9H) ppm.
^{13}C NMR (101 MHz, $CDCl_3$) δ = 172.5, 165.4, 153.6, 129.5, 127.6, 119.9, 79.2, 63.5, 51.3, 28.5, 25.1 ppm.
IR (neat) ν 3319, 1744, 1667, 1532, 1488, 1234, 1134, 1092, 828 cm^{-1}
HRMS (ESI): *m/z*: calcd for $C_{16}H_{23}ClNO_4^+$ $[M+H]^+$: 328.13101, Found: 328.13049

2-(*tert*-butylamino)-2-oxoethyl 2,6-difluorobenzoate 85
Eluent: Pet/EtOAc 75:25. White powder, yield 55%, mp: 59.0-59.8 °C

^1H NMR (400 MHz, $CDCl_3$) δ (referred to the major rotamer) = 7.56 – 7.48 (m, 1H), 7.08 – 7.00 (m, 2H), 6.36 (br s, 1H), 4.75 (s, 2H), 1.41 (s, 9H) ppm.
^{13}C NMR (101 MHz, $CDCl_3$) δ (referred to the major rotamer) = 165.7, 161.1 (dd, *J*=258.2, 5.6 Hz), 159.4 (t, *J*=1.6 Hz), 134.0 (t, *J*=11 Hz), 112.5 (dd, *J*= 22.9, 3.2 Hz), 109.6 (t, *J*=16.4 Hz), 63.7, 51.5, 28.6 ppm.
IR (neat) ν 3364, 1720, 1674, 1537, 1470, 1257, 1126, 1003 cm^{-1}
HRMS (ESI): *m/z*: calcd for $C_{13}H_{16}F_2NO_3^+$ $[M+H]^+$: 272.10928, Found: 272.10861

2-(*tert*-butylamino)-2-oxoethyl 2-bromobenzoate 86
Eluent: Pet/EtOAc 6:4. White powder, yield 68%, mp: 114.9-115.5 °C
^1H NMR (400 MHz, $CDCl_3$) δ = 7.90 – 7.85 (m, 1H), 7.71 (dd, *J*=7.6, 1.4 Hz, 1H), 7.48 – 7.38 (m, 2H), 6.37 (br s, 1H), 4.74 (s, 2H), 1.43 (s, 9H) ppm.
^{13}C NMR (101 MHz, $CDCl_3$) δ = 165.7, 164.9, 134.4, 133.3, 132.2, 131.4, 127.6, 121.1, 64.4, 51.6, 28.8 ppm.
IR (neat) ν 3281, 1733, 1659, 1562, 1243, 1220, 1109, 749 cm^{-1}
HRMS (ESI): *m/z*: calcd for $C_{13}H_{17}BrNO_3^+$ $[M+H]^+$: 314.03920 – 316.03715 , Found: 314.03811 – 316.03602

2-(*tert*-butylamino)-2-oxoethyl 2-iodobenzoate 87
Eluent: Pet/EtOAc 8:2. White powder, yield 62%, mp: 128.3-129.0 °C
^1H NMR (400 MHz, $CDCl_3$) δ = 8.00 (d, *J*=7.9 Hz, 1H), 7.81 (d, *J*=7.7 Hz, 1H), 7.45 (t, *J*=7.6 Hz, 1H), 7.20 (td, *J*=7.7, 1.7 Hz, 1H), 6.25 (br s, 1H), 4.70 (s, 2H), 1.40 (s, 9H) ppm.
^{13}C NMR (101 MHz, $CDCl_3$) δ = 165.7, 165.4, 141.3, 134.8, 133.2, 131.4, 128.2, 93.6, 64.4, 51.7, 28.8 ppm.
IR (neat) ν 3277, 1730, 1658, 1561, 1242, 1219, 1102, 745 cm^{-1}
HRMS (ESI): *m/z*: calcd for $C_{13}H_{17}INO_3^+$ $[M+H]^+$: 362.02476, Found: 362.02435

2-(*tert*-butylamino)-2-oxoethyl [1,1'-biphenyl]-2-carboxylate 88
Eluent: Pet/EtOAc 7:3. Light yellow oil, yield 76%
^1H NMR (400 MHz, $CDCl_3$) δ = 7.87 (dd, *J*=7.7, 1.4 Hz, 1H), 7.60 (td, *J*=7.6, 1.4 Hz, 1H), 7.49 – 7.38 (m, 5H), 7.38 – 7.33 (m, 2H), 5.36 (br s, 1H), 4.38 (s, 2H), 1.23 (s, 9H) ppm.
^{13}C NMR (101 MHz, $CDCl_3$) δ = 167.6, 165.5, 142.1, 141.1, 132.0, 130.8, 130.3, 130.0, 128.7, 128.1, 127.7, 127.5, 63.9, 51.1, 28.5 ppm.
IR (neat) ν 3309, 1727, 1668, 1530, 1240, 1123, 1099, 746 cm^{-1}
HRMS (ESI): *m/z*: calcd for $C_{19}H_{22}NO_3^+$ $[M+H]^+$: 312.15942, Found: 312.15848

2-(*tert*-butylamino)-2-oxoethyl 3-bromo-2-methylbenzoate 89
Eluent: Pet/EtOAc 7:3. White powder, yield 63%, mp: 95.0-95.7 °C
^1H NMR (400 MHz, $CDCl_3$) δ = 7.78 – 7.72 (m, 2H), 7.14 (t, *J*=7.9 Hz, 1H), 5.95 (br s, 1H), 4.67 (s, 2H), 2.65 (s, 3H), 1.39 (s, 9H) ppm.
^{13}C NMR (101 MHz, $CDCl_3$) δ = 165.9, 165.8, 138.9, 136.4, 131.6, 129.1, 127.3, 127.0, 63.9, 51.6, 28.8, 20.7 ppm.
IR (neat) ν 3275, 1732, 1660, 1564, 1209, 1152, 1103, 753 cm^{-1}
HRMS (ESI): *m/z*: calcd for $C_{14}H_{19}BrNO_3^+$ $[M+H]^+$: 328.05485 - 330.05280, Found: 328.05342 - 330.05124

2-(*tert*-butylamino)-2-oxoethyl 2-chlorobenzoate 90
Eluent: Pet/EtOAc 8:2. White powder, yield 61%, mp: 100.6-101.3 °C
^1H NMR (400 MHz, $CDCl_3$) δ = 7.97 – 7.91 (m, 1H), 7.54 – 7.50 (m, 2H), 7.45 – 7.38 (m, 1H), 6.43 (br s, 1H), 4.75 (s, 2H), 1.43 (s, 9H) ppm.
^{13}C NMR (101 MHz, $CDCl_3$) δ = 164.4, 133.4, 133.1, 132.4, 131.2, 129.1, 127.1, 64.3, 51.6, 28.7 ppm.
IR (neat) ν 3282, 1733, 1658, 1562, 1244, 1118, 1059, 939, 751 cm^{-1}
HRMS (ESI): *m/z*: calcd for $C_{13}H_{17}ClNO_3^+$ $[M+H]^+$: 270.08915, Found: 270.08841

2-(*tert*-butylamino)-2-oxoethyl 2-(4-bromophenyl)-2-methylpropanoate 91
Eluent: Pet/EtOAc 8:2. Colorless oil, yield 65%

^1H NMR (400 MHz, $CDCl_3$) δ = 7.54 – 7.47 (m, 2H), 7.30 – 7.23 (m, 2H), 5.26 (br s, 1H), 4.44 (s, 2H), 1.63 (s, 6H), 1.20 (s, 9H) ppm.
^{13}C NMR (101 MHz, $CDCl_3$) δ (referred to the major rotamer) = 174.5, 166.0, 143.0, 131.8, 127.5, 121.2, 63.1, 51.2, 46.2, 28.5, 26.1 ppm.
IR (neat) ν 3324, 1738, 1668, 1526, 1225, 1138, 1098, 1008 cm^{-1}
HRMS (ESI): *m/z*: calcd for $C_{16}H_{23}BrNO_3^+$ $[M+H]^+$: 356.08615 - 358.08410 , Found: 356.08487 - 358.08254

2-(*tert*-butylamino)-2-oxoethyl 2-phenylbutanoate 92
Eluent: Pet/EtOAc 7:3. White powder, yield 60%, mp: 90.2-90.8 °C
^1H NMR (400 MHz, $CDCl_3$) δ = 7.41 – 7.29 (m, 5H), 5.39 (br s, 1H), 4.63 (d, *J*=15.3 Hz, 1H), 4.32 (d, *J*=15.3 Hz, 1H), 3.54 (t, *J*=7.7 Hz, 1H), 2.24 – 2.11 (m, 1H), 1.93 – 1.80 (m, 1H), 1.18 (s, 9H), 0.93 (t, *J*=7.4 Hz, 3H) ppm.
^{13}C NMR (101 MHz, $CDCl_3$) δ = 172.2, 166.1, 138.6, 129.0, 127.9, 127.7, 62.9, 53.2, 51.1, 28.5, 26.1, 12.1 ppm.
IR (neat) ν 3272, 1734, 1659, 1566, 1263, 1196, 1157, 700 cm^{-1}
HRMS (ESI): *m/z*: calcd for $C_{16}H_{24}NO_3^+$ $[M+H]^+$: 278.17507, Found: 278.17459

1-(*tert*-butylamino)-3-methyl-1-oxobutan-2-yl 2,6-dimethylbenzoate 93
Eluent: Pet/EtOAc 9:1. White powder, yield 58%, mp: 101.3-102.2 °C
^1H NMR (400 MHz, $CDCl_3$) δ (referred to the major rotamer) = 7.23 (t, *J*=7.6 Hz, 1H), 7.07 (d, *J*=7.7 Hz, 2H), 5.88 (br s, 1H), 5.13 (d, *J*=5.0 Hz, 1H), 2.37 (s, 6H), 2.36 – 2.30 (m, 1H), 1.38 (s, 9H), 1.07 (d, *J*=7.0 Hz, 3H), 1.03 (d, *J*=6.9 Hz, 3H) ppm.
^{13}C NMR (101 MHz, $CDCl_3$) δ (referred to the major rotamer) = 169.1, 168.2, 134.7, 133.5, 129.6, 127.7, 79.7, 51.5, 30.7, 28.7, 20.0, 18.9, 17.5 ppm.
IR (neat) ν 3311, 1730, 1652, 1545, 1244, 1111, 981, 769 cm^{-1}
HRMS (ESI): *m/z*: calcd for $C_{18}H_{28}NO_3^+$ $[M+H]^+$: 306.20637, Found: 306.20575

2-(benzylamino)-2-oxoethyl 2,6-dimethylbenzoate 94
Eluent: Pet/EtOAc 7:3. Colorless oil, yield 39%
^1H NMR (400 MHz, $CDCl_3$) δ = 7.39 – 7.27 (m, 5H), 7.25 (t, *J*=7.6 Hz, 1H), 7.07 (d, *J*=7.7 Hz, 2H), 6.52 (br s, 1H), 4.87 (s, 2H), 4.53 (d, *J*=5.8 Hz, 2H), 2.31 (s, 6H) ppm.
^{13}C NMR (101 MHz, $CDCl_3$) δ (referred to the major rotamer) = 168.3, 166.7, 137.5, 135.2, 132.6, 129.9, 128.8, 127.9, 127.8 (2C), 63.3, 43.2, 19.8 ppm.
IR (neat) ν 3295, 1737, 1661, 1537, 1258, 1074, 1013, 793, cm^{-1}
HRMS (ESI): *m/z*: calcd for $C_{18}H_{20}NO_3^+$ $[M+H]^+$: 298.14377, Found: 298.14334

2-(*tert*-butylamino)-2-oxoethyl 2,6-dichlorobenzoate 95
Eluent: Pet/EtOAc 8:2. White powder, yield 70%, mp: 80.1-81.1 °C
^1H NMR (400 MHz, $CDCl_3$) δ = 7.39 – 7.34 (m, 3H), 6.14 (br s, 1H), 4.77 (s, 2H), 1.38 (s, 9H) ppm.
^{13}C NMR (101 MHz, $CDCl_3$) δ = 165.3, 163.1, 132.5, 131.8, 131.6, 128.1, 64.1, 51.7, 28.7 ppm.
IR (neat) ν 3309, 1747, 1669, 1555, 1433, 1258, 1143, 785 cm^{-1}
HRMS (ESI): *m/z*: calcd for $C_{13}H_{16}Cl_2NO_3^+$ $[M+H]^+$: 304.05018 , Found: 304.04968

2-(*tert*-butylamino)-2-oxoethyl 2-chloro-6-(trifluoromethyl)benzoate 96
Eluent: Pet/EtOAc 8:2. White powder, yield 21%, mp: 91.5-92.5 °C
^1H NMR (400 MHz, $CDCl_3$) δ = 7.69 (m, 2H), 7.58 (t, *J*=8.0 Hz, 1H), 6.06 (br s, 1H), 4.78 (s, 2H), 1.39 (s, 9H) ppm.
^{13}C NMR (101 MHz, $CDCl_3$) δ = 165.1, 163.3, 133.4, 132.4, 131.2, 130.8 (br q), 129.2 (q, *J*=33.0 Hz), 124.8 (q, *J*=4.6 Hz), 122.7 (q, *J*=275.0 Hz), 64.5, 51.6, 28.6 ppm.
IR (neat) ν 3315, 1748, 1670, 1553, 1314, 1258, 1129, 805 cm^{-1}
HRMS (ESI): *m/z*: calcd for $C_{14}H_{16}ClF_3NO_3^+$ $[M+H]^+$: 338.07653, Found: 338.07588

2-(*tert*-butylamino)-2-oxoethyl (1*S*,3*s*)-adamantane-1-carboxylate 97

Eluent: Pet/EtOAc 8:2. White powder, yield 86%, mp: 147.8-148.5 °C
^{1}H NMR (400 MHz, $CDCl_3$) δ = 5.84 (br s, 1H), 4.42 (s, 2H), 2.08 – 2.00 (m, 3H), 1.95 – 1.88 (m, 6H), 1.79 – 1.66 (m, 6H), 1.36 (s, 9H) ppm.
^{13}C NMR (101 MHz, $CDCl_3$) δ = 175.8, 166.4, 62.8, 51.2, 40.7, 38.8, 36.3, 28.7, 27.8 ppm.
IR (neat) ν 3278, 1737, 1675, 1561, 1216, 1183, 1092, 580 cm^{-1}
HRMS (ESI): *m/z*: calcd for $C_{17}H_{28}NO_3^+$ $[M+H]^+$: 294.20637, Found: 294.20582

2-(*tert*-butylamino)-2-oxoethyl 3-bromo-2,6-dimethylbenzoate 98
Eluent: Pet/EtOAc 8:2. Colorless oil, yield 77%
^{1}H NMR (400 MHz, $CDCl_3$) δ (referred to the major rotamer) = 7.55 – 7.48 (m, 1H), 6.99 – 6.92 (m, 1H), 5.92 (br s, 1H), 4.70 (s, 2H), 2.38 (s, 3H), 2.29 (s, 3H), 1.39 (s, 9H) ppm.
^{13}C NMR (101 MHz, $CDCl_3$) δ (referred to the major rotamer) = 167.7, 165.5, 134.7, 134.3, 134.1, 133.7, 129.2, 122.8, 63.8, 51.6, 28.8, 20.5, 19.4 ppm.
IR (neat) ν 3314, 1735, 1665, 1542, 1454, 1241, 1147, 1099 cm^{-1}
HRMS (ESI): *m/z*: calcd for $C_{15}H_{21}BrNO_3^+$ $[M+H]^+$: 342.07050 - 344.06845, Found: 342.06934 - 344.06705

1,3-phenylenebis(2-(cyclohexylamino)-2-oxoethane-1,1-diyl) bis(2,6-dimethylbenzoate) 100
White powder, yield 62%, mp: 174.6-175.3 °C
^{1}H NMR (400 MHz, $CDCl_3$) δ (as mixture of the enantiomers plus *meso* form) = 7.63 (dt, *J*=8.1, 1.9 Hz, 2H), 7.57 – 7.51 (m, 4H), 7.47 – 7.40 (m, 2H), 7.24 (t, *J*=7.6 Hz, 4H), 7.05 (dd, *J*=7.7, 2.6 Hz, 8H), 6.35 (s, 2H), 6.34 (s, 2H), 6.13 – 6.05 (m, 4H), 3.88 – 3.76 (m, 4H), 2.25 (s, 12H), 2.24 (s, 12H), 1.98 – 1.85 (m, 8H), 1.78 – 1.65 (m, 12H), 1.42 – 1.29 (m, 8H), 1.21 – 1.07 (m, 12H) ppm.
^{13}C NMR (101 MHz, CDCl3) δ (as mixture of the enantiomers plus *meso* form) = 168.1, 168.0, 166.8, 166.7, 136.3, 136.2, 135.1, 132.8, 132.7, 129.8, 129.8, 129.3, 129.2, 128.5, 127.7, 127.7, 126.8, 126.5, 75.6, 75.6, 48.4, 33.0, 25.4, 24.8, 24.8, 19.7, 19.7 ppm.
IR (neat) ν 3246, 1729, 1651, 1263, 1241, 1112, 1071, 768 cm^{-1}
HRMS (ESI): *m/z*: calcd for $C_{40}H_{49}N_2O_6^+$ $[M+H]^+$: 653.35851, Found: 653.35773

Copies of ^{1}H and ^{13}C spectra

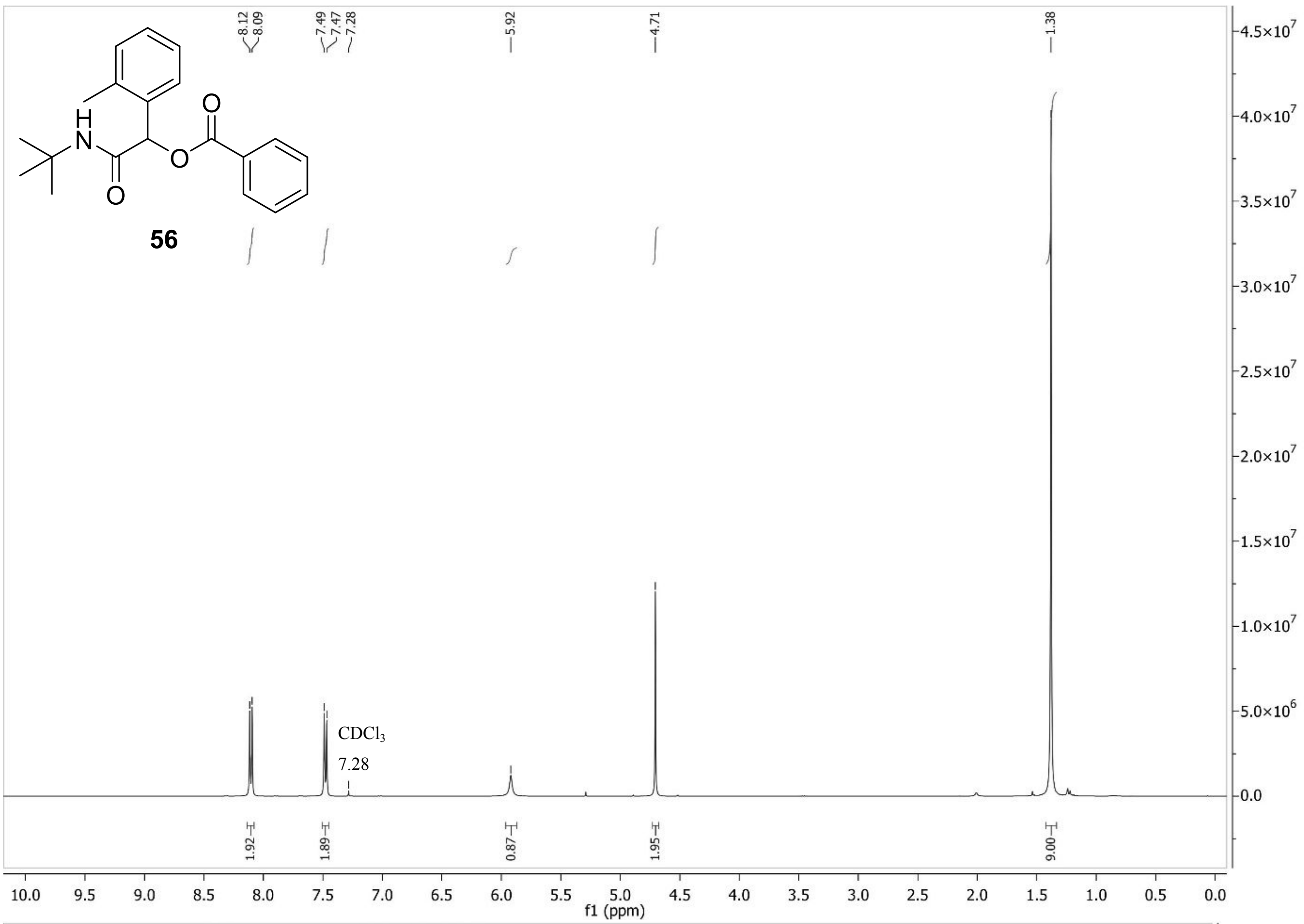

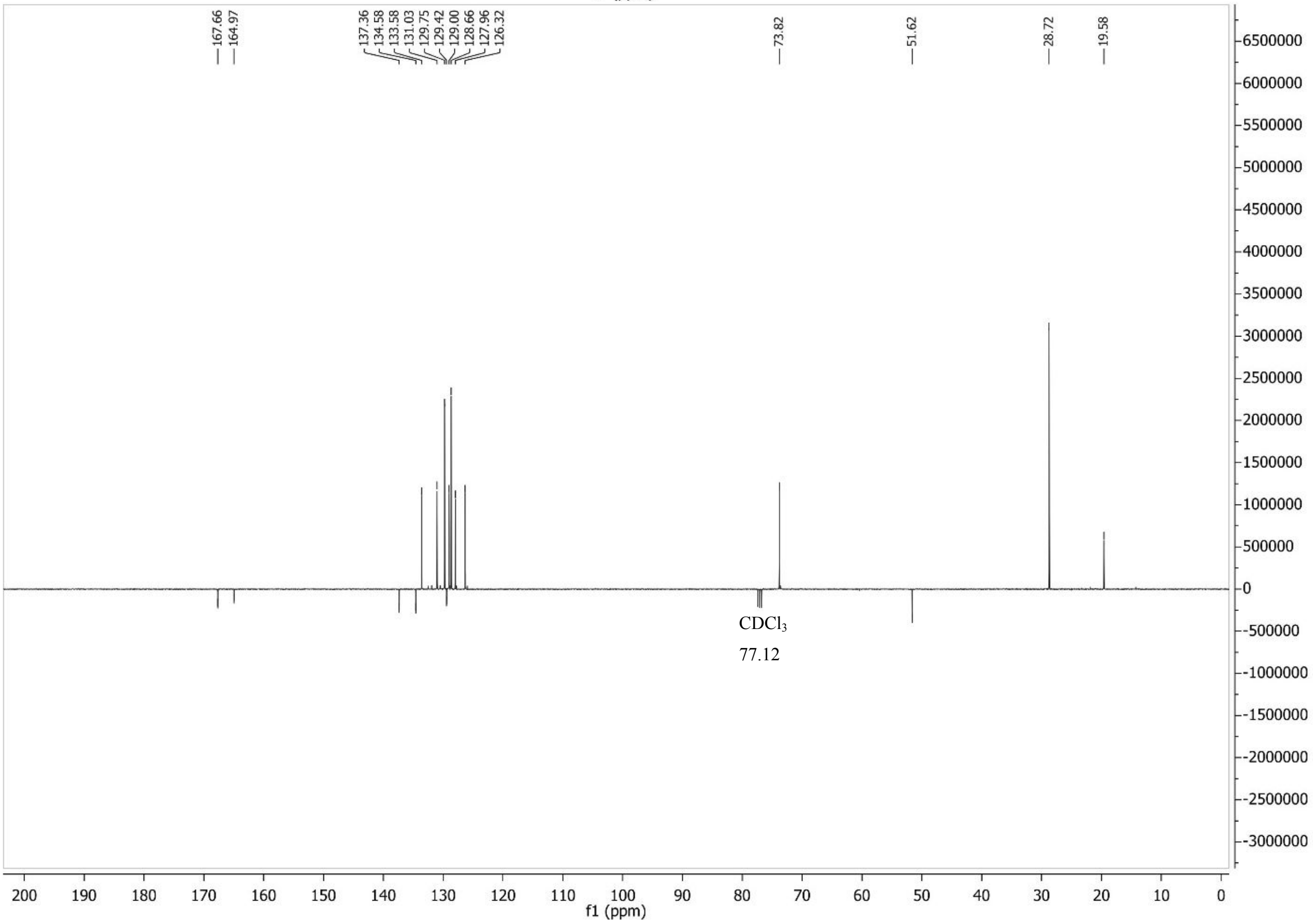

^{1}H and ^{13}C NMR spectra of compound **56**

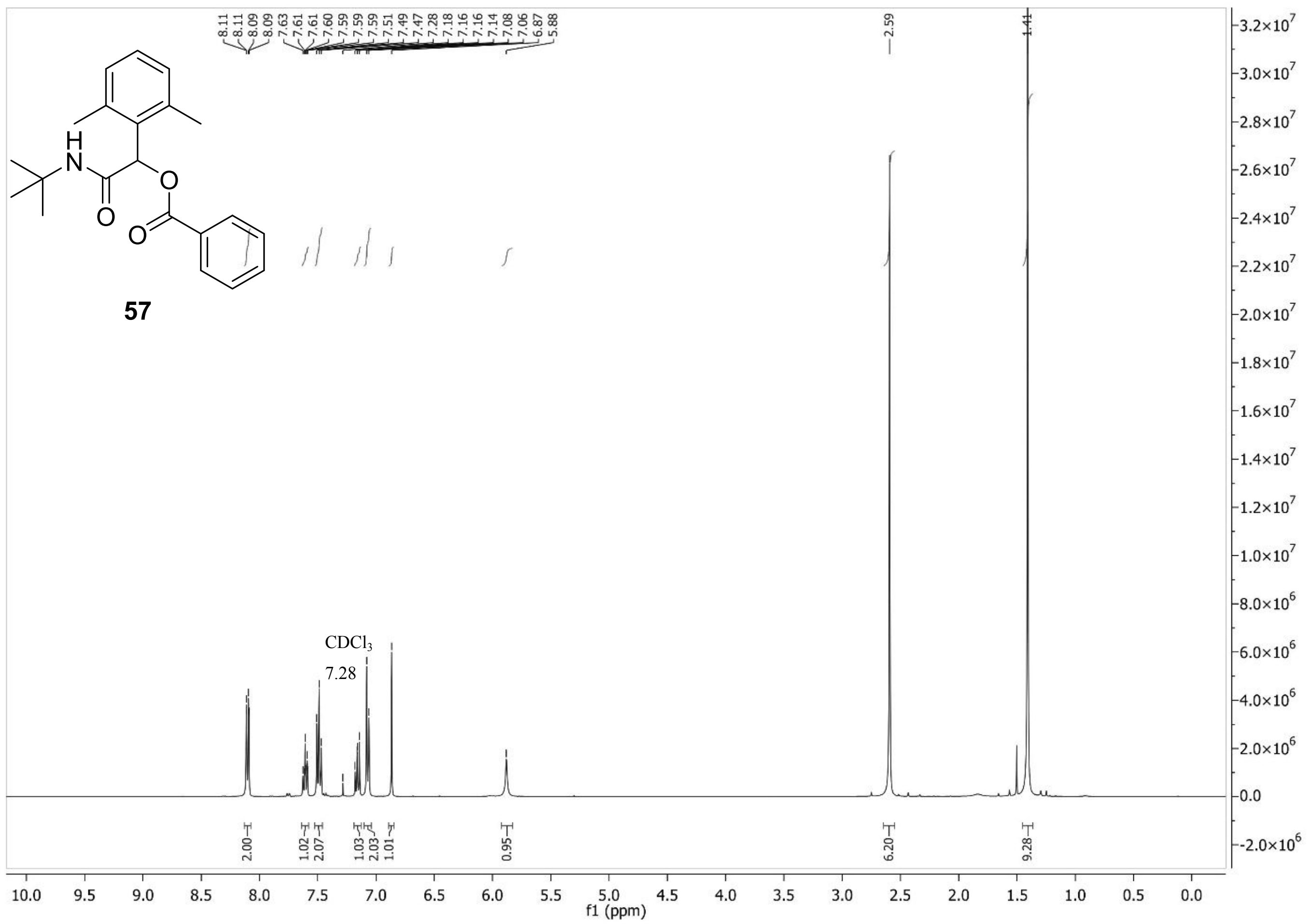

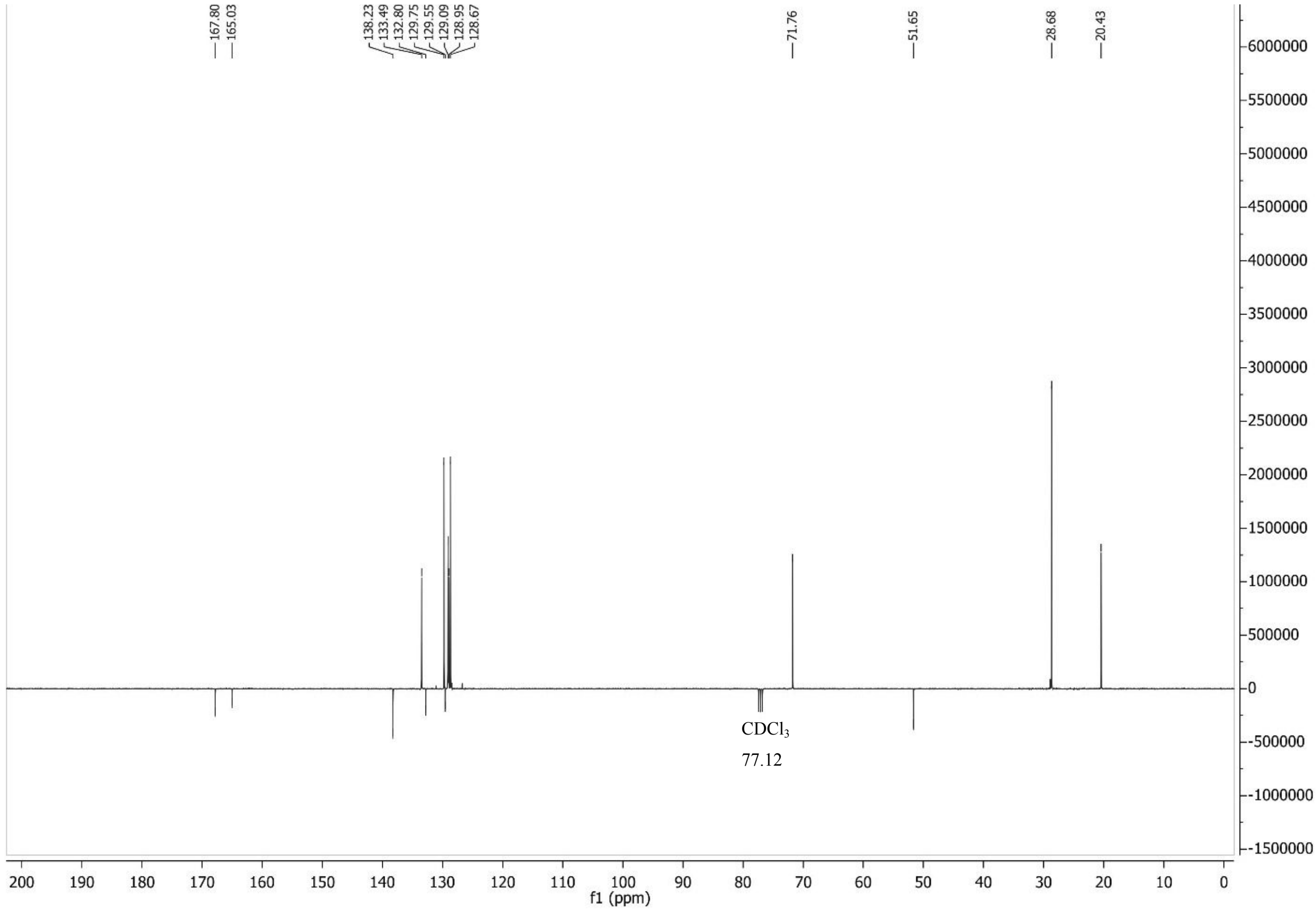

^{1}H and ^{13}C NMR spectra of compound **57**

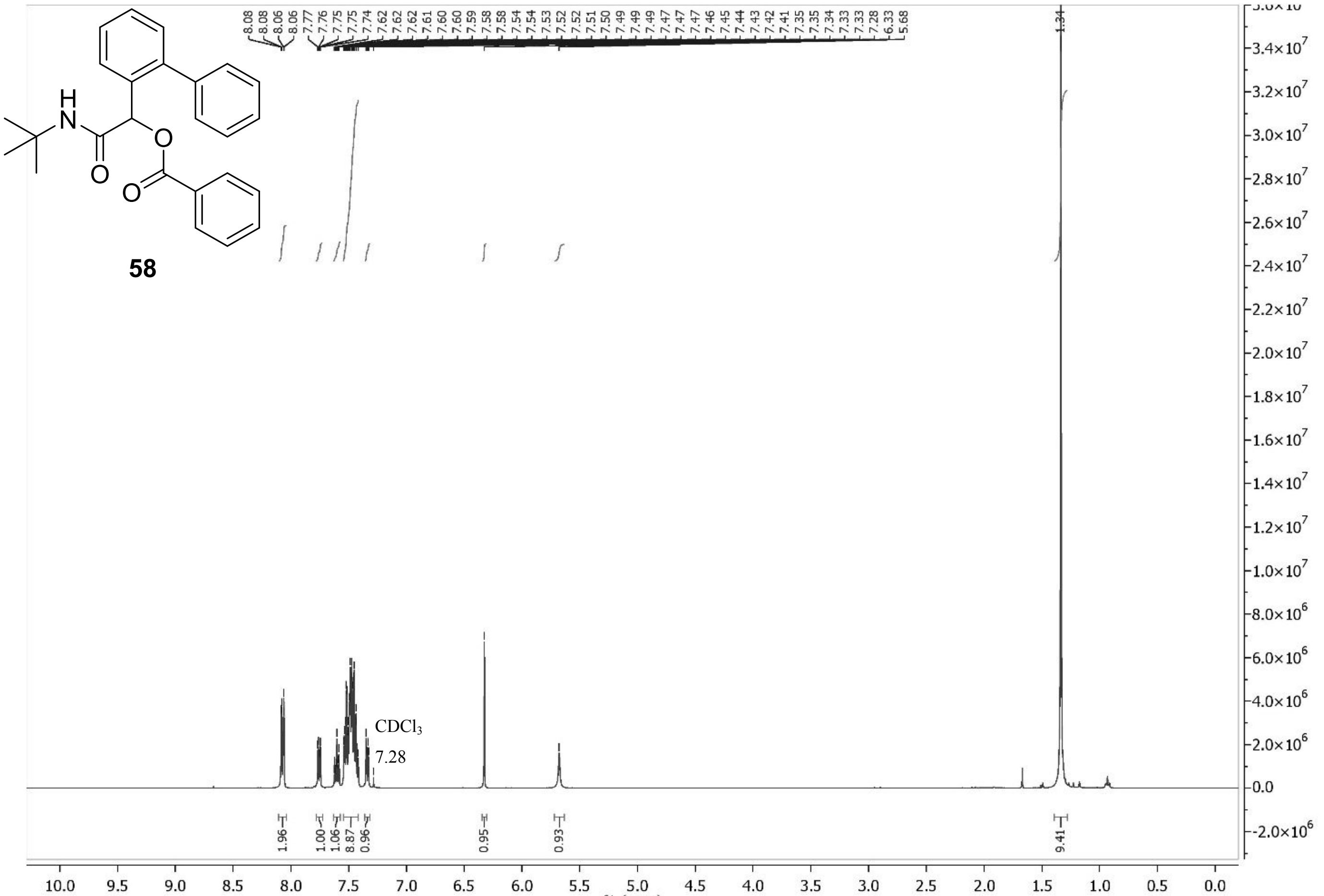

58

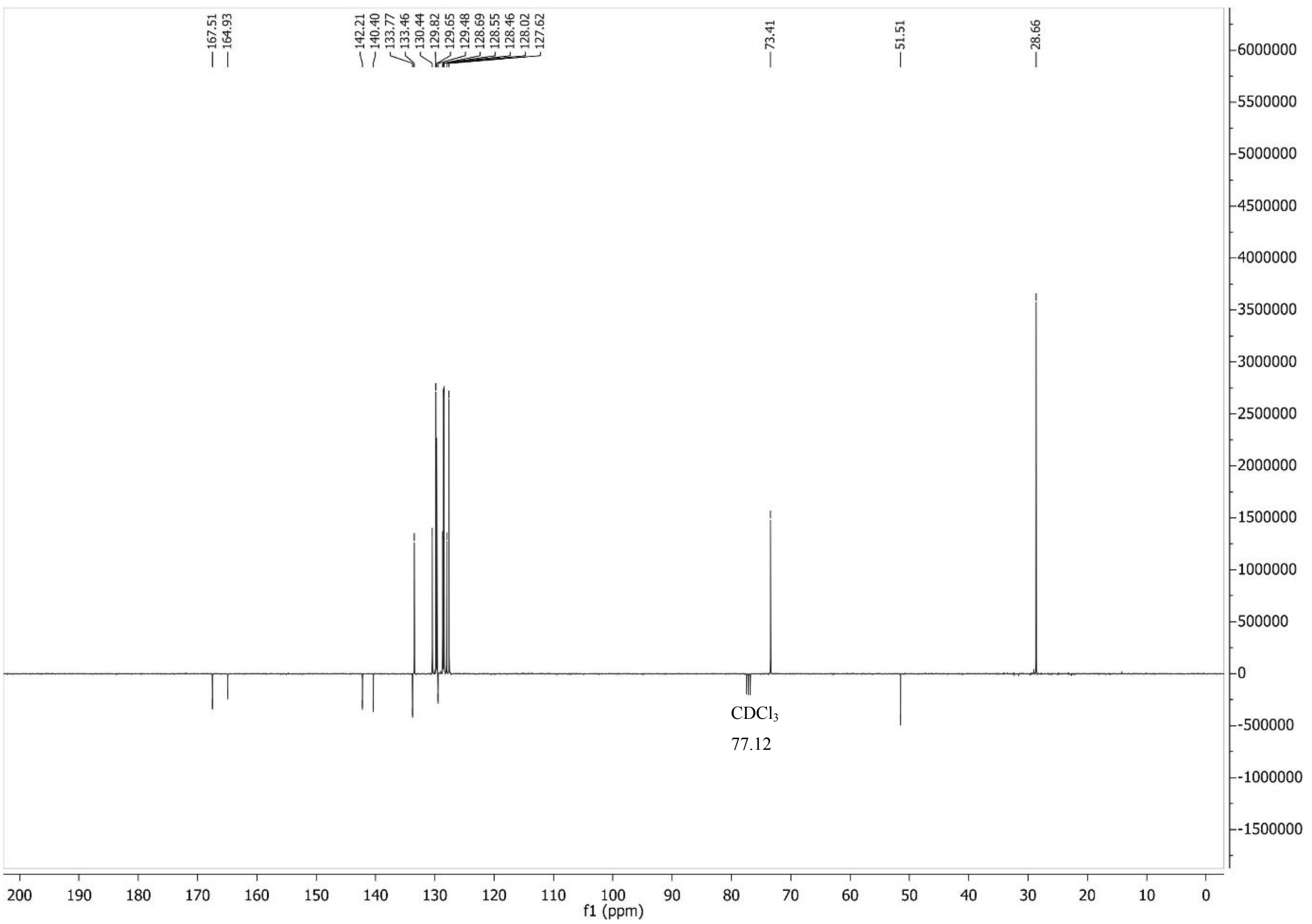

^1H and ^{13}C NMR spectra of compound **58**

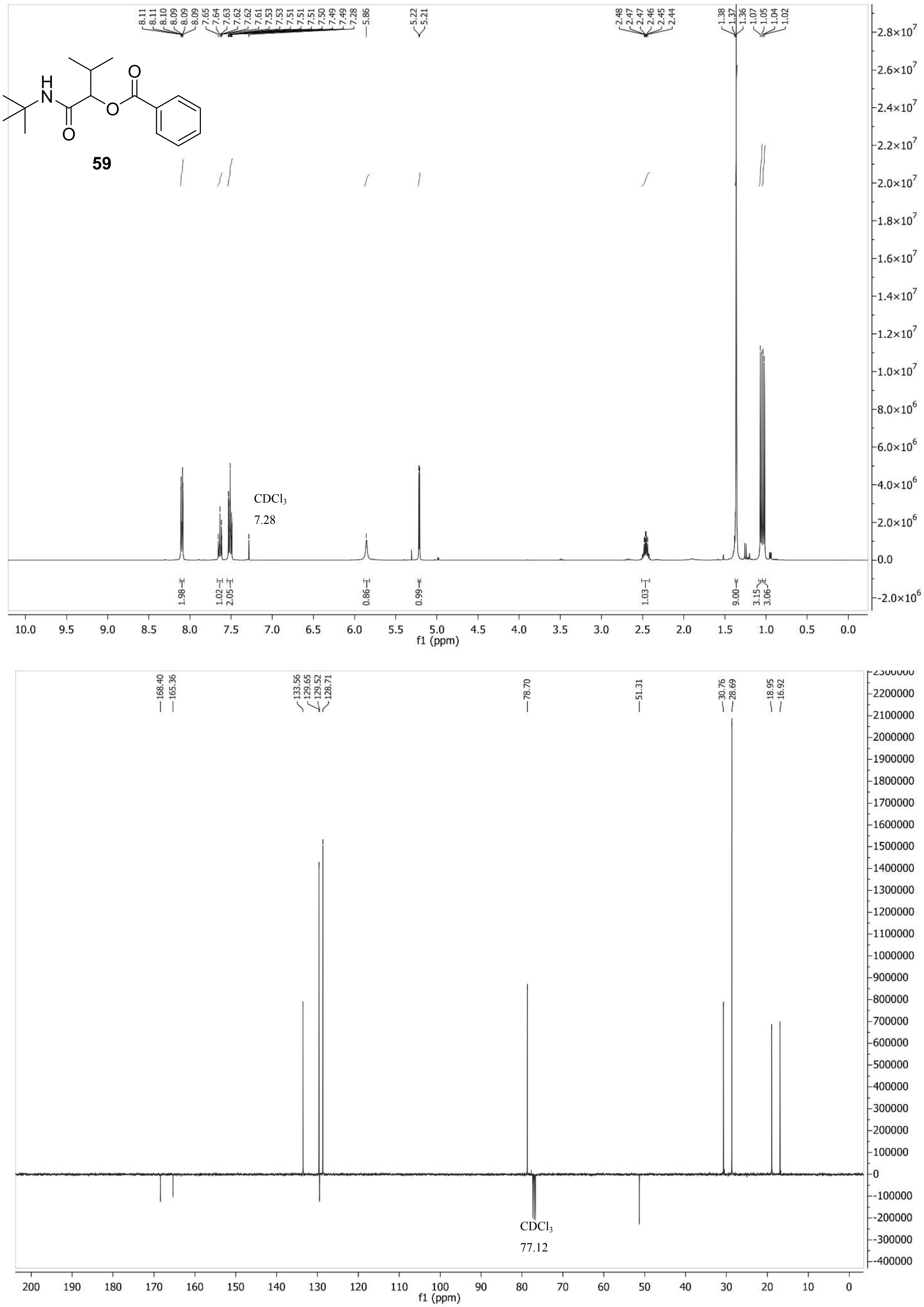

^{1}H and ^{13}C NMR spectra of compound **59**

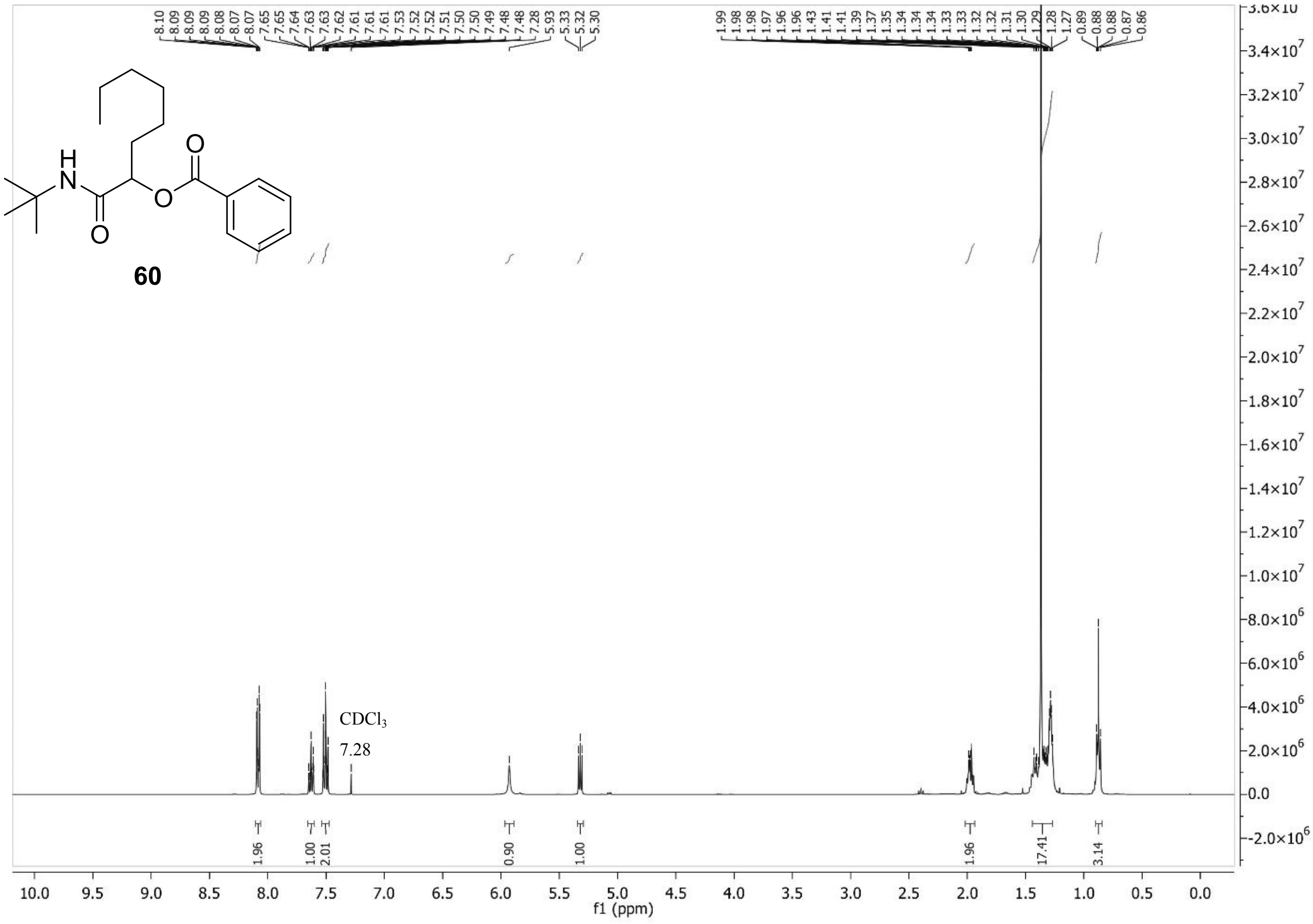

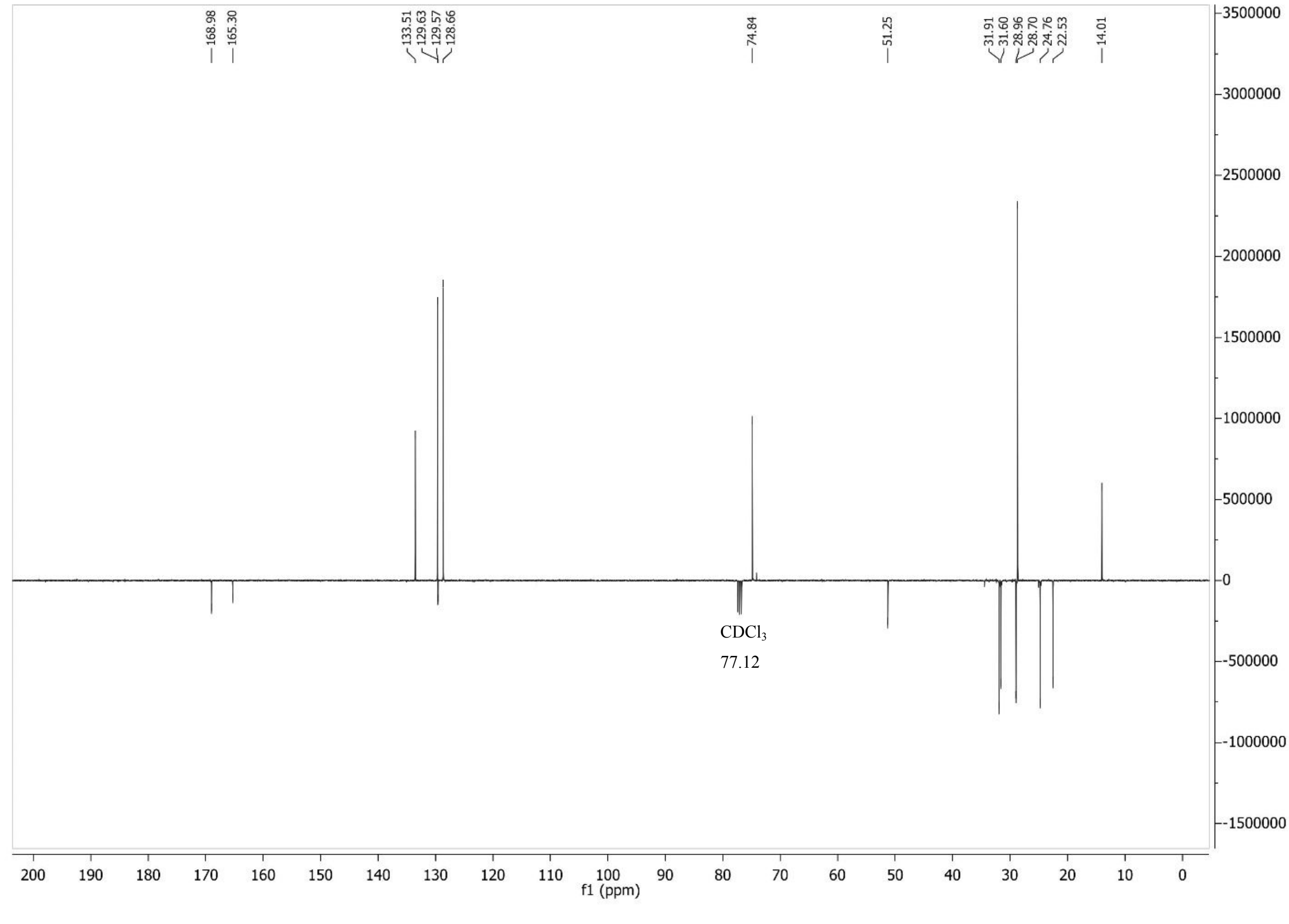

^{1}H and ^{13}C NMR spectra of compound **60**

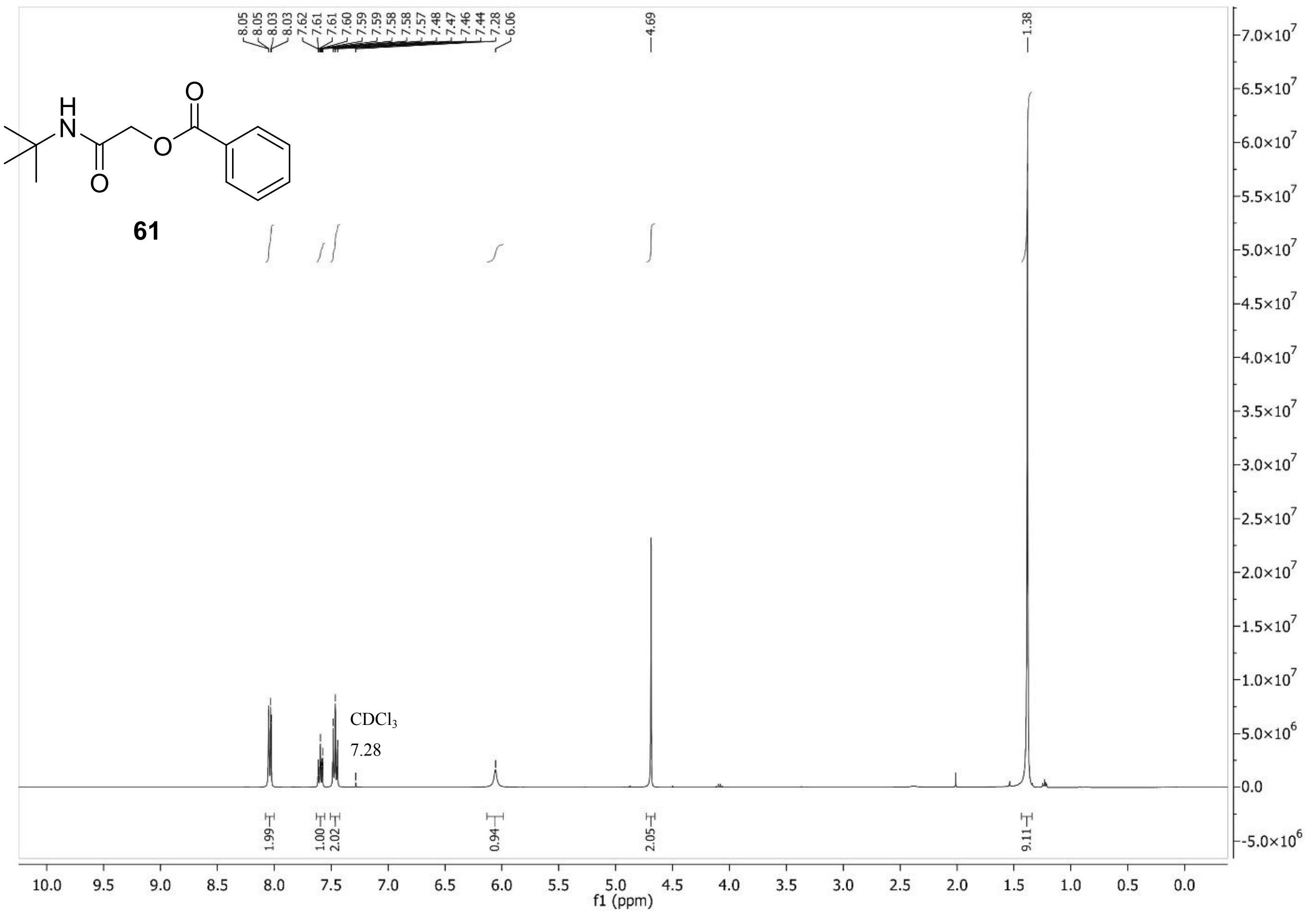

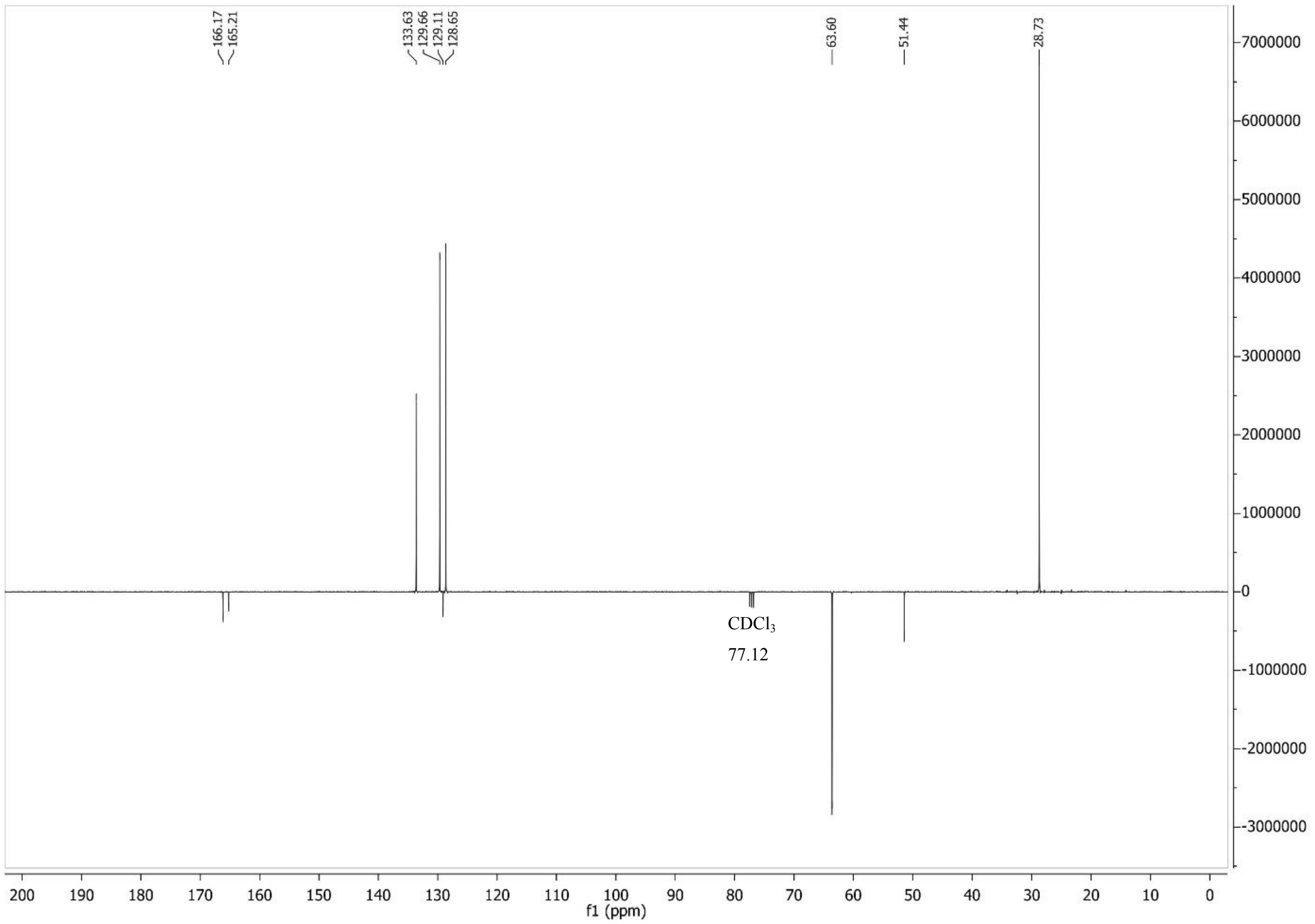

^{1}H and ^{13}C NMR spectra of compound **61**

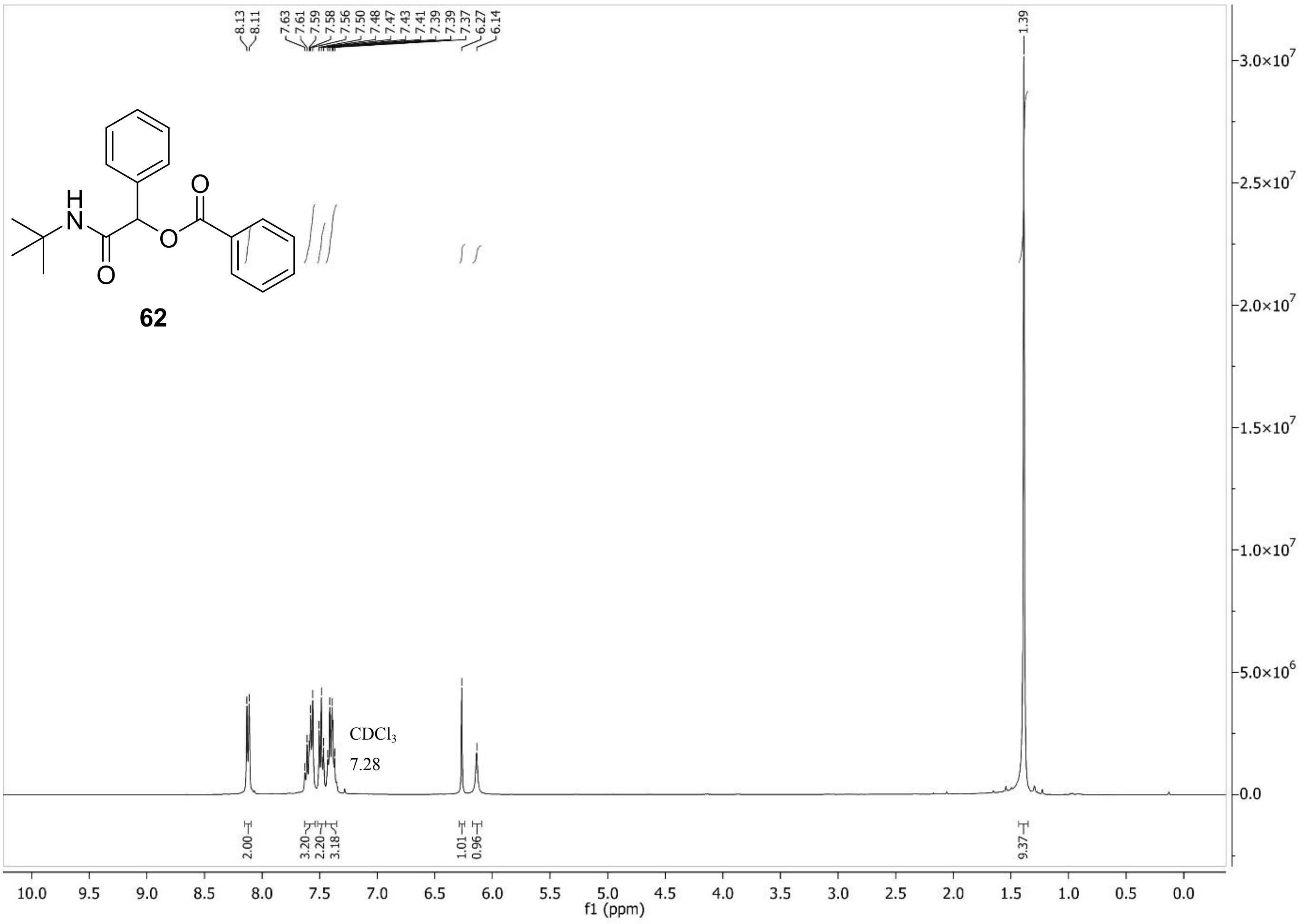

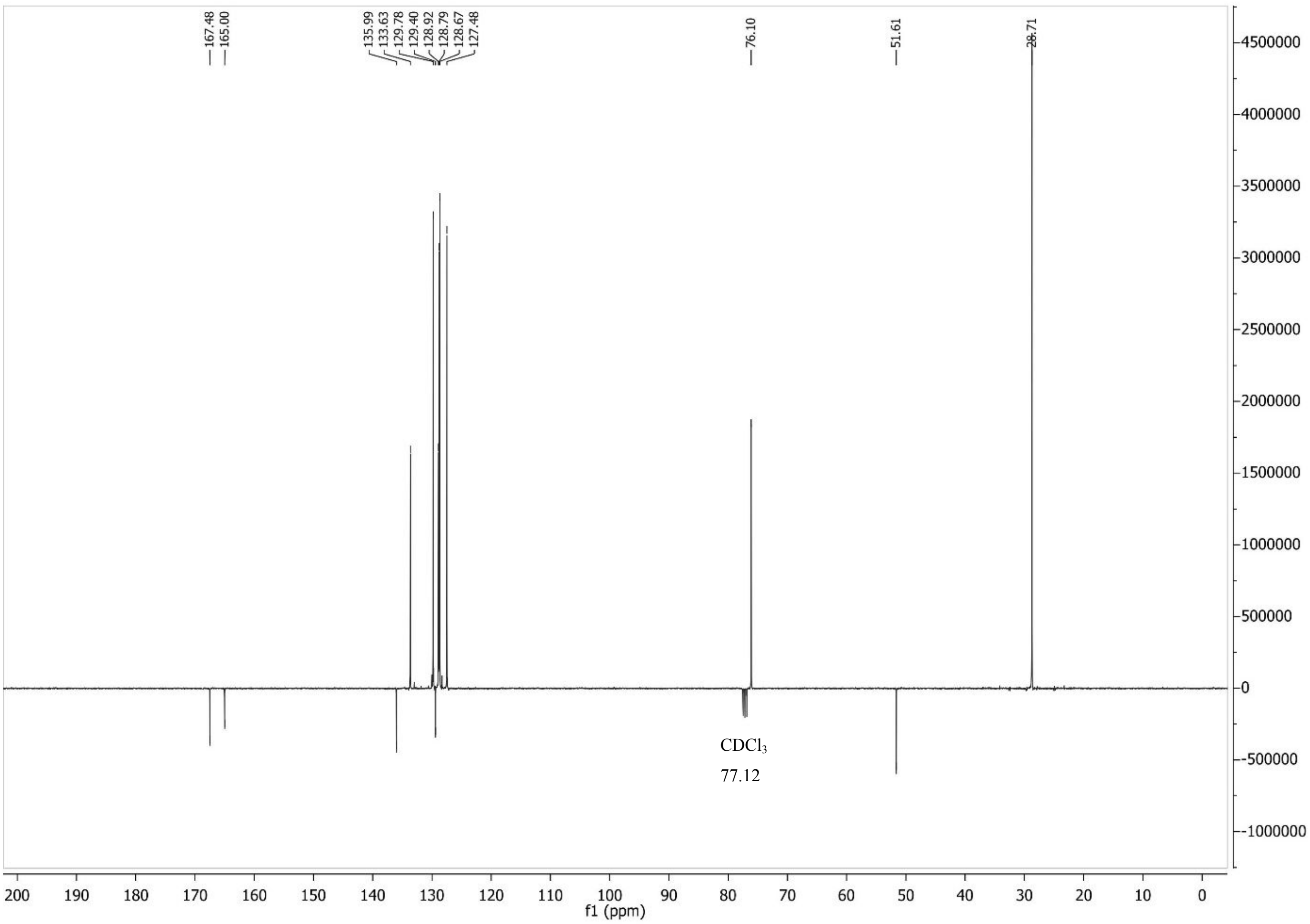

^{1}H and ^{13}C NMR spectra of compound **62**

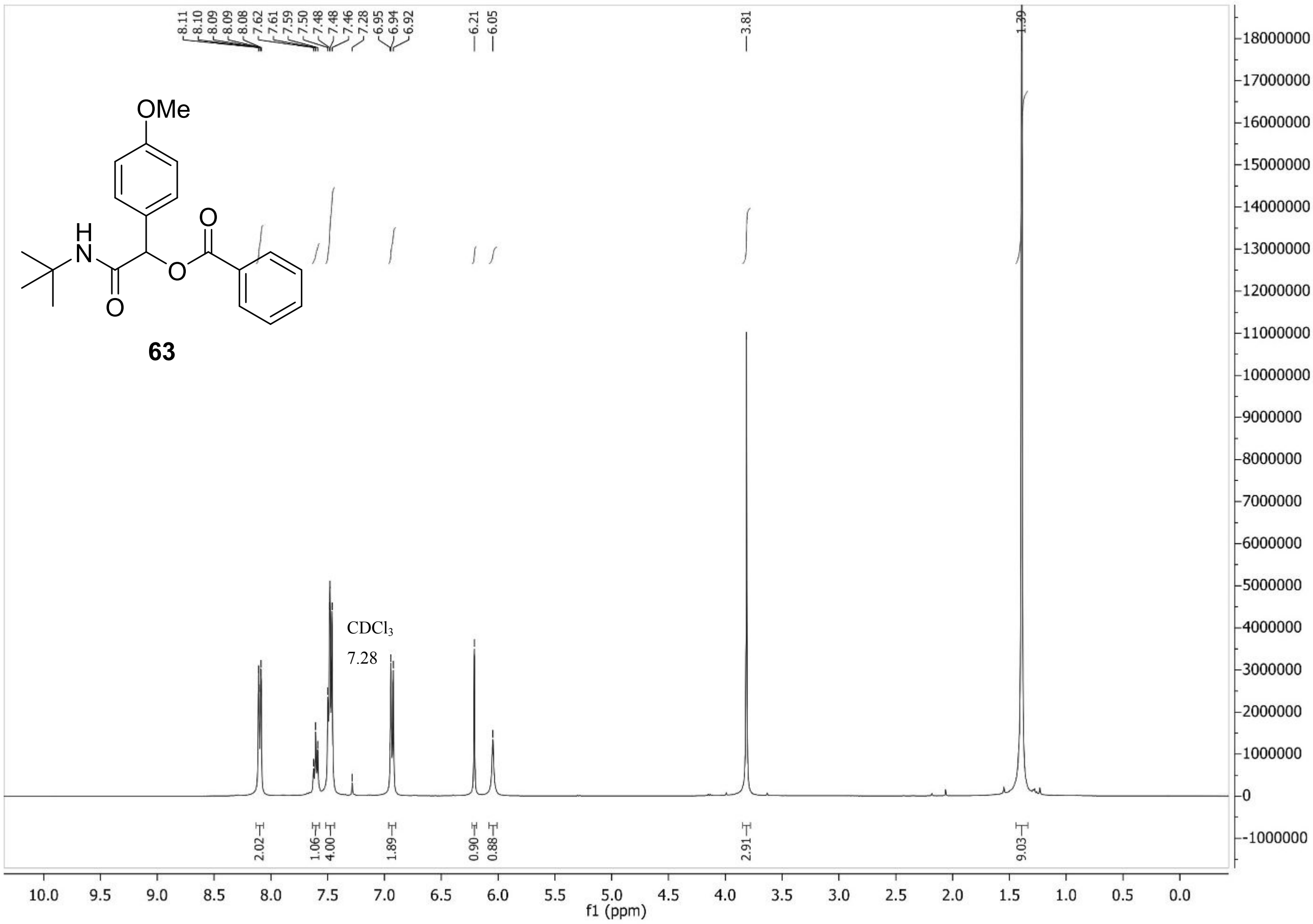

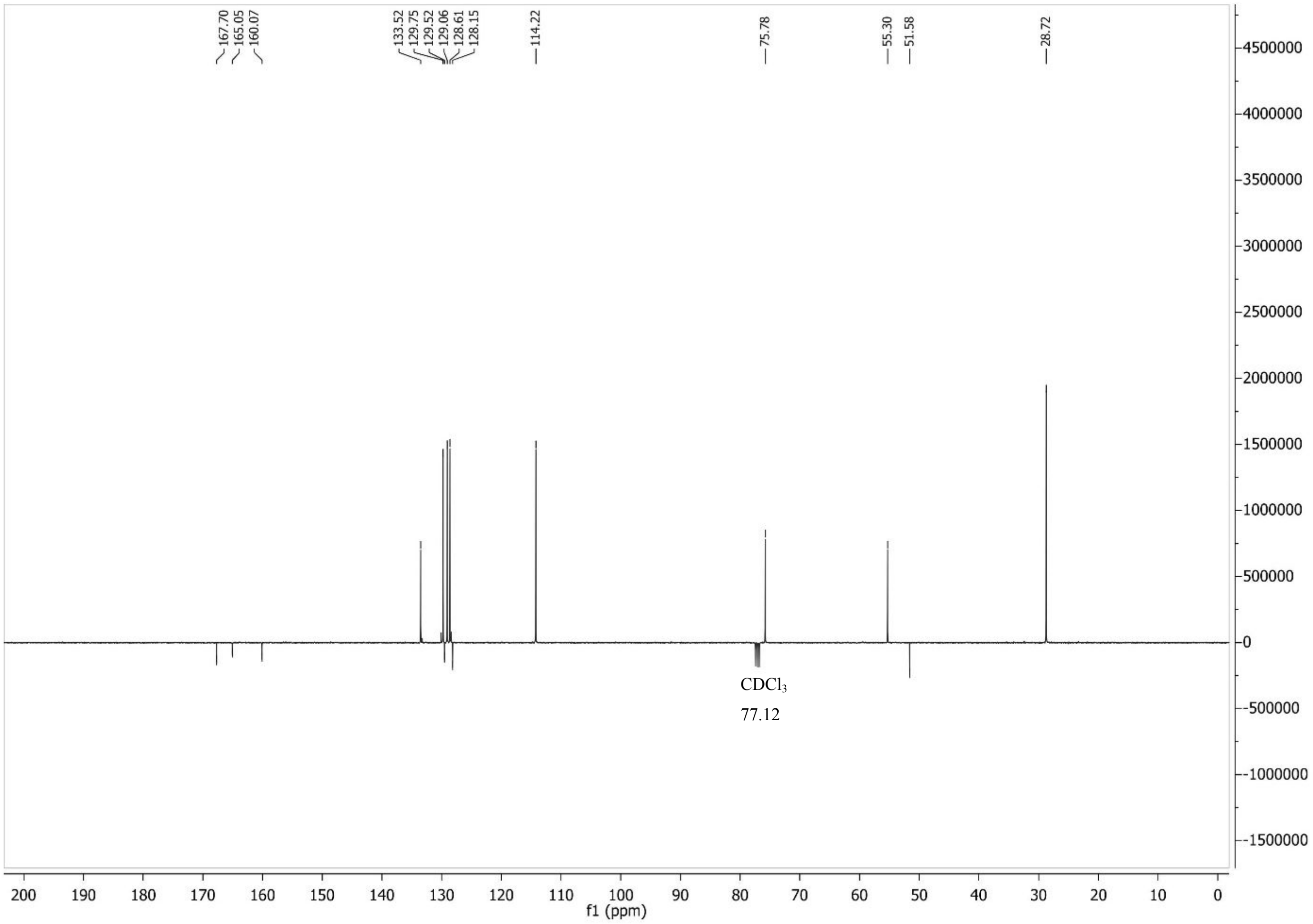

^{1}H and ^{13}C NMR spectra of compound **63**

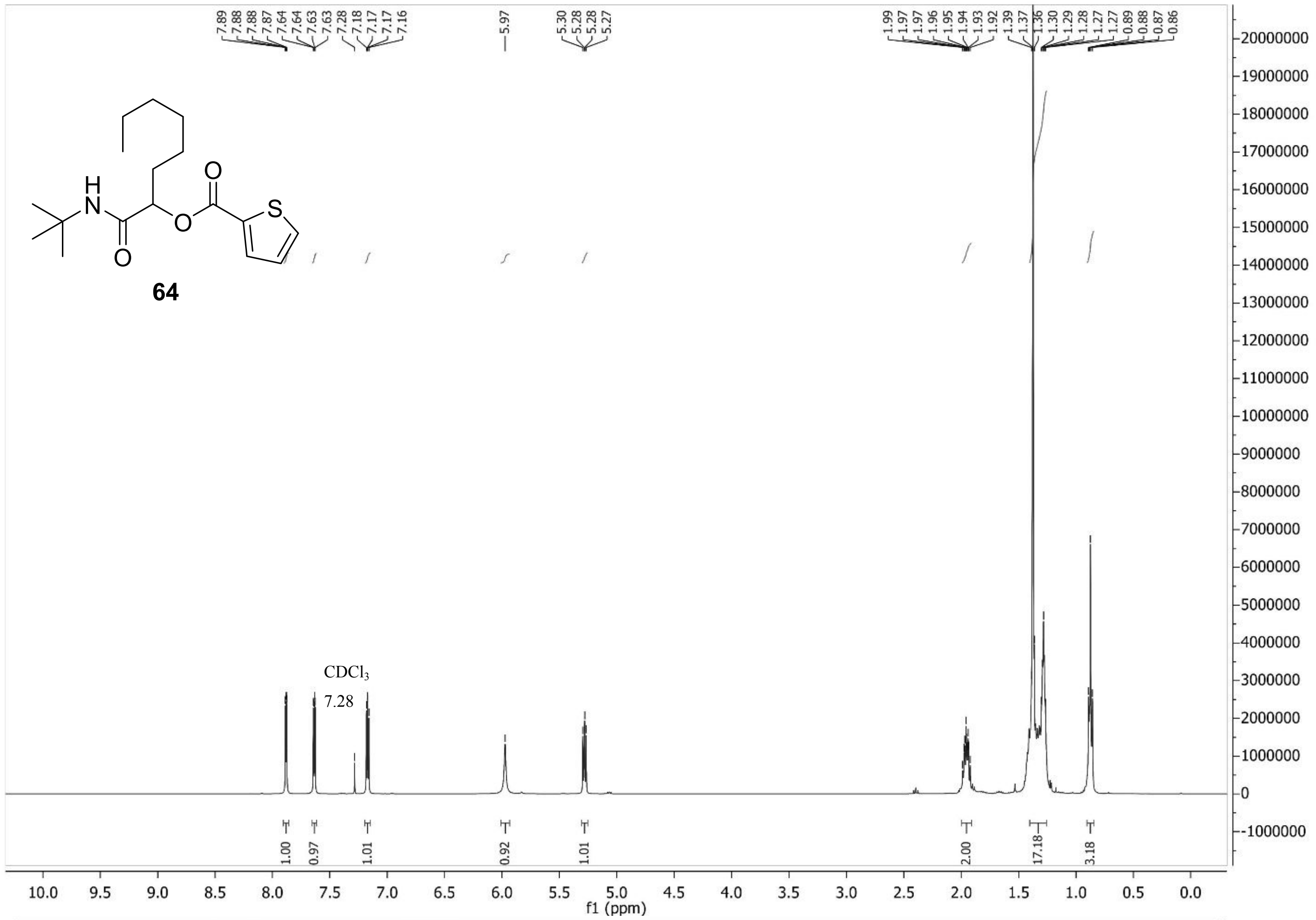

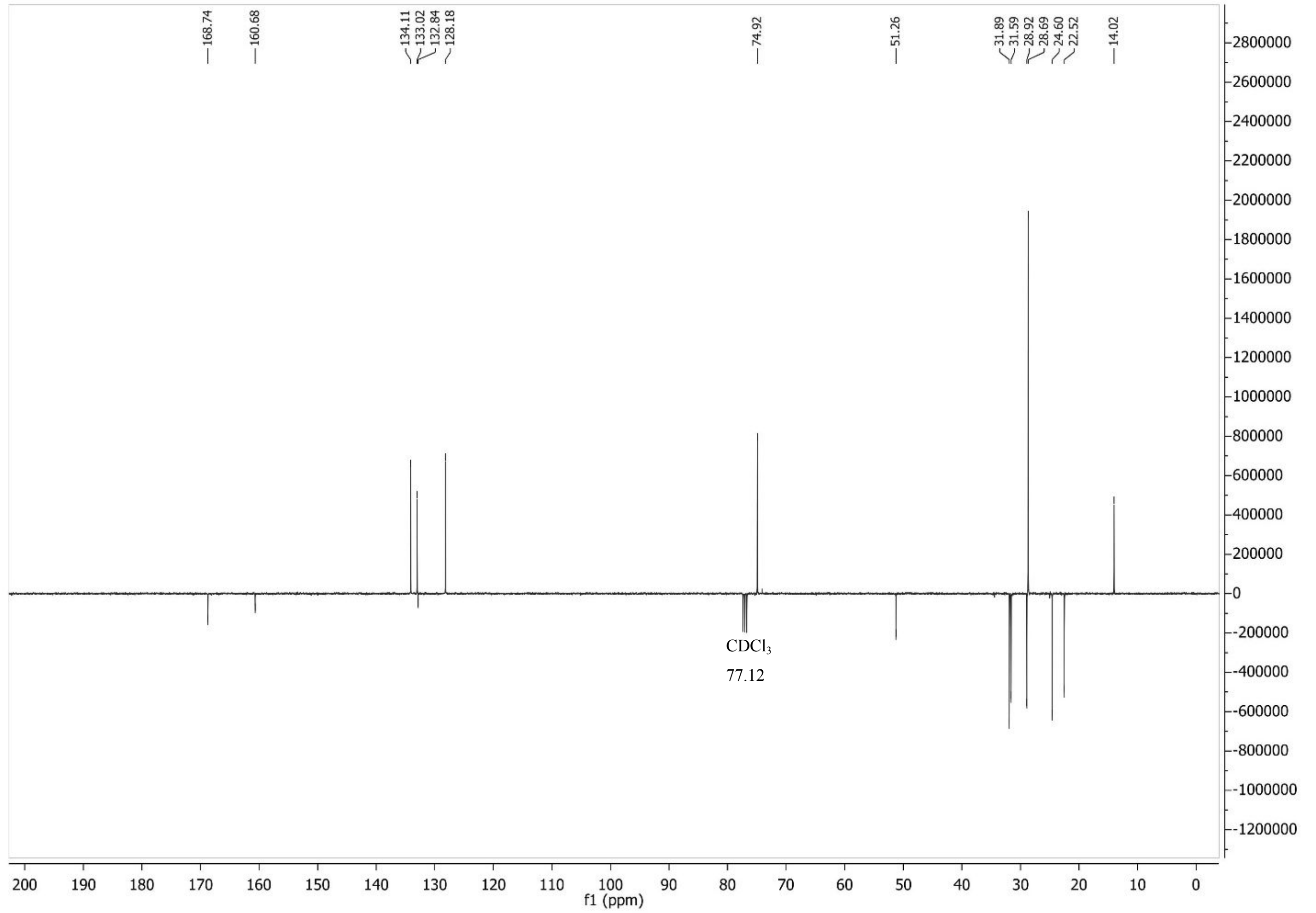

^{1}H and ^{13}C NMR spectra of compound **64**

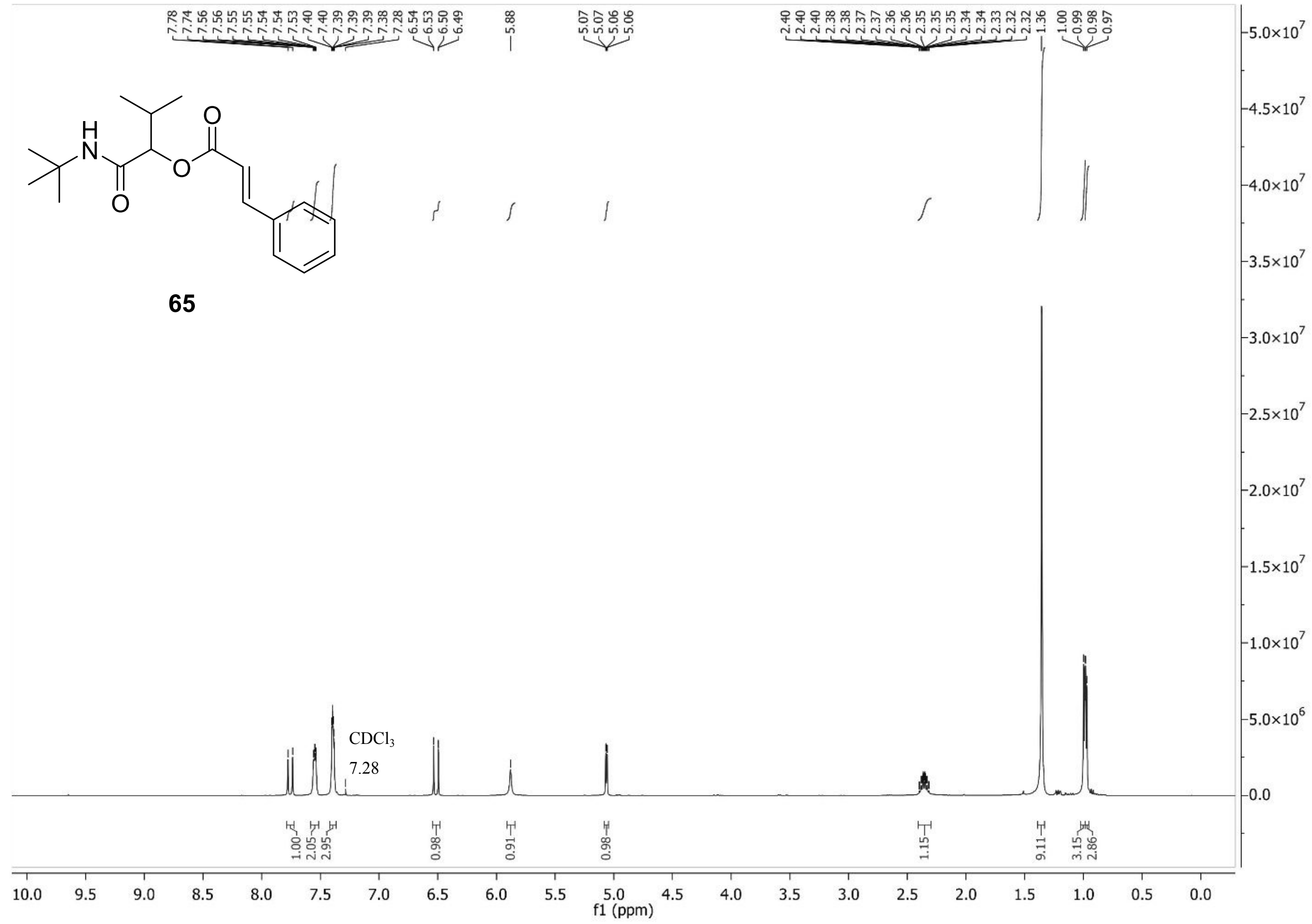

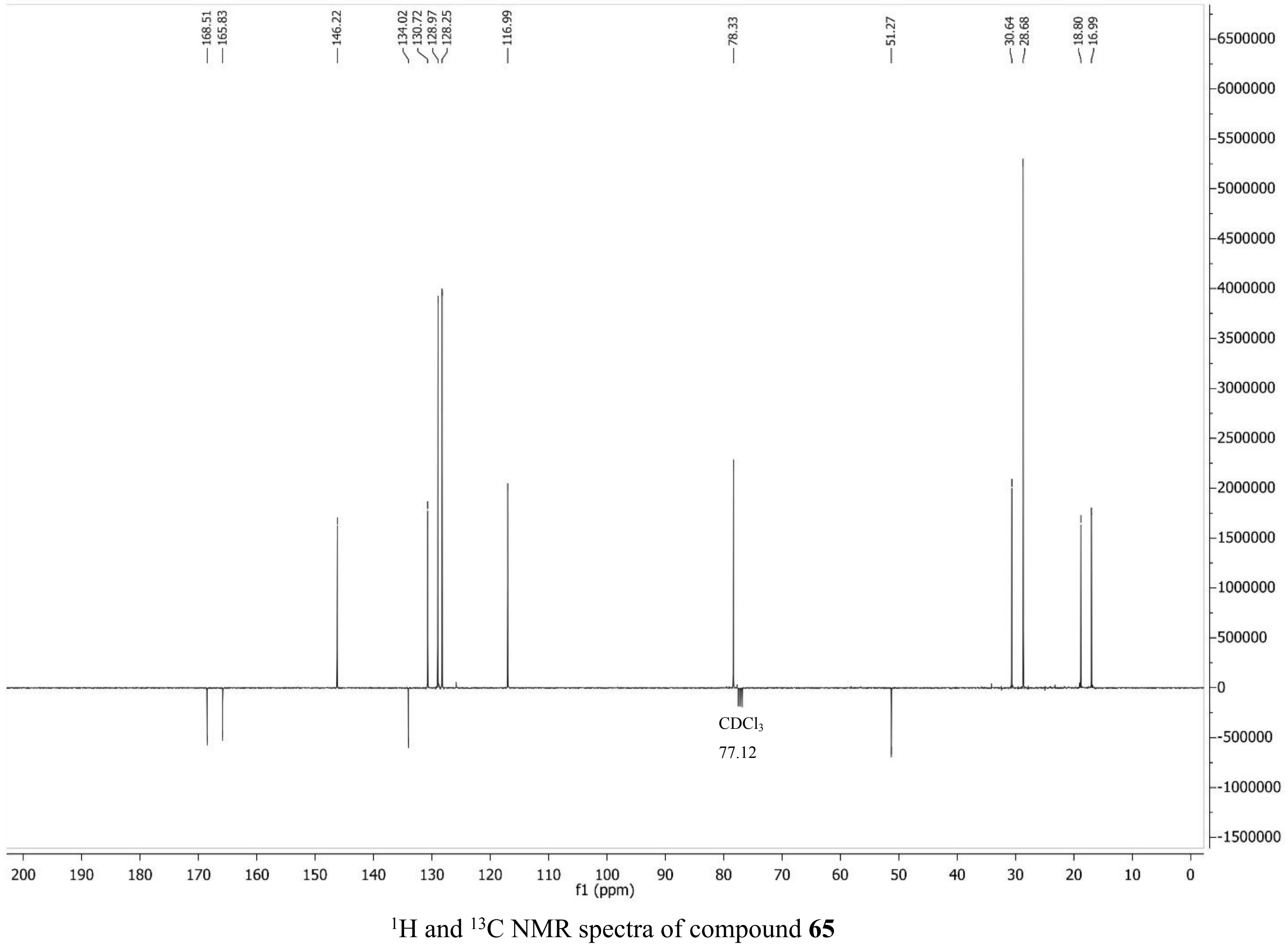

^{1}H and ^{13}C NMR spectra of compound **65**

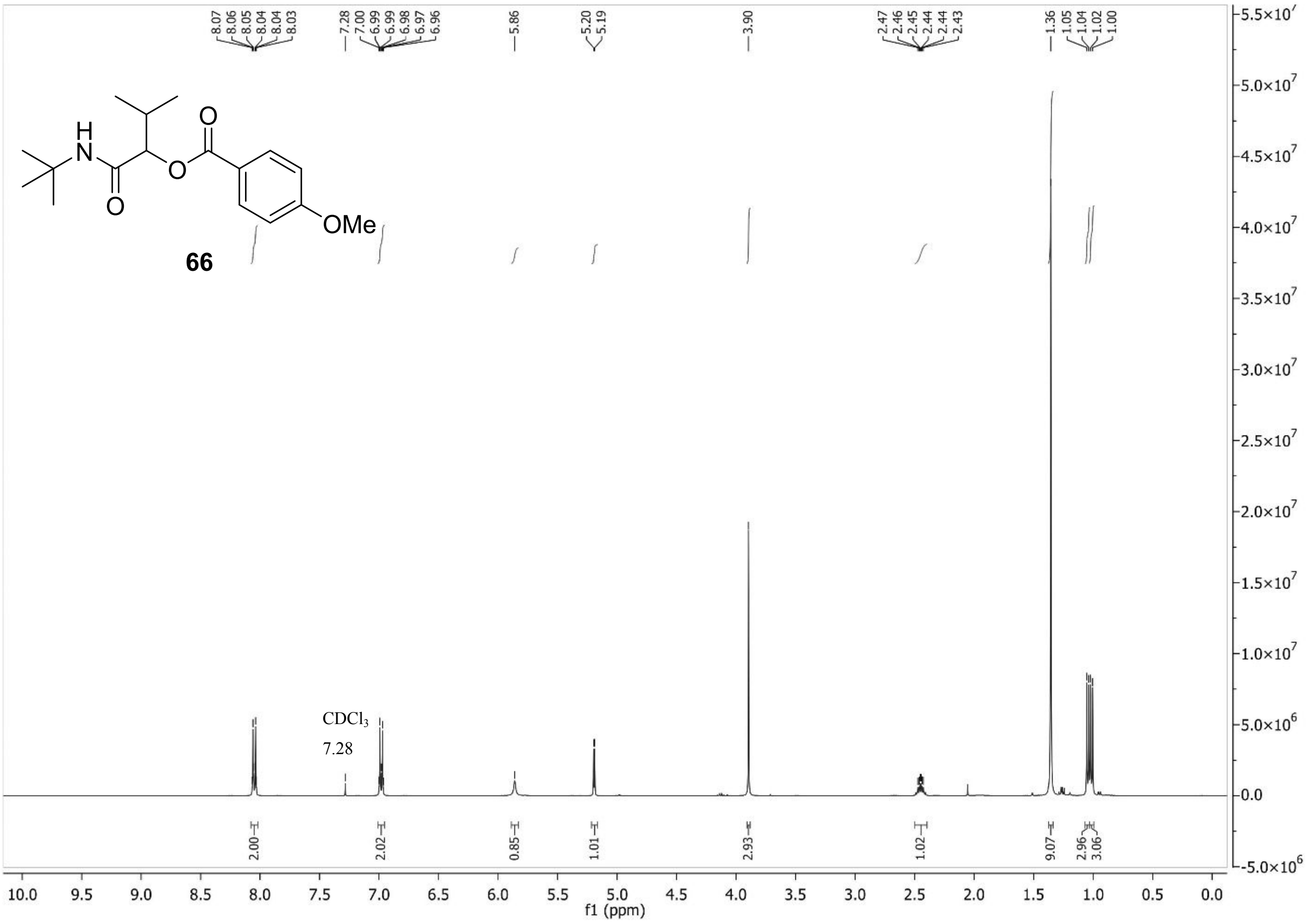

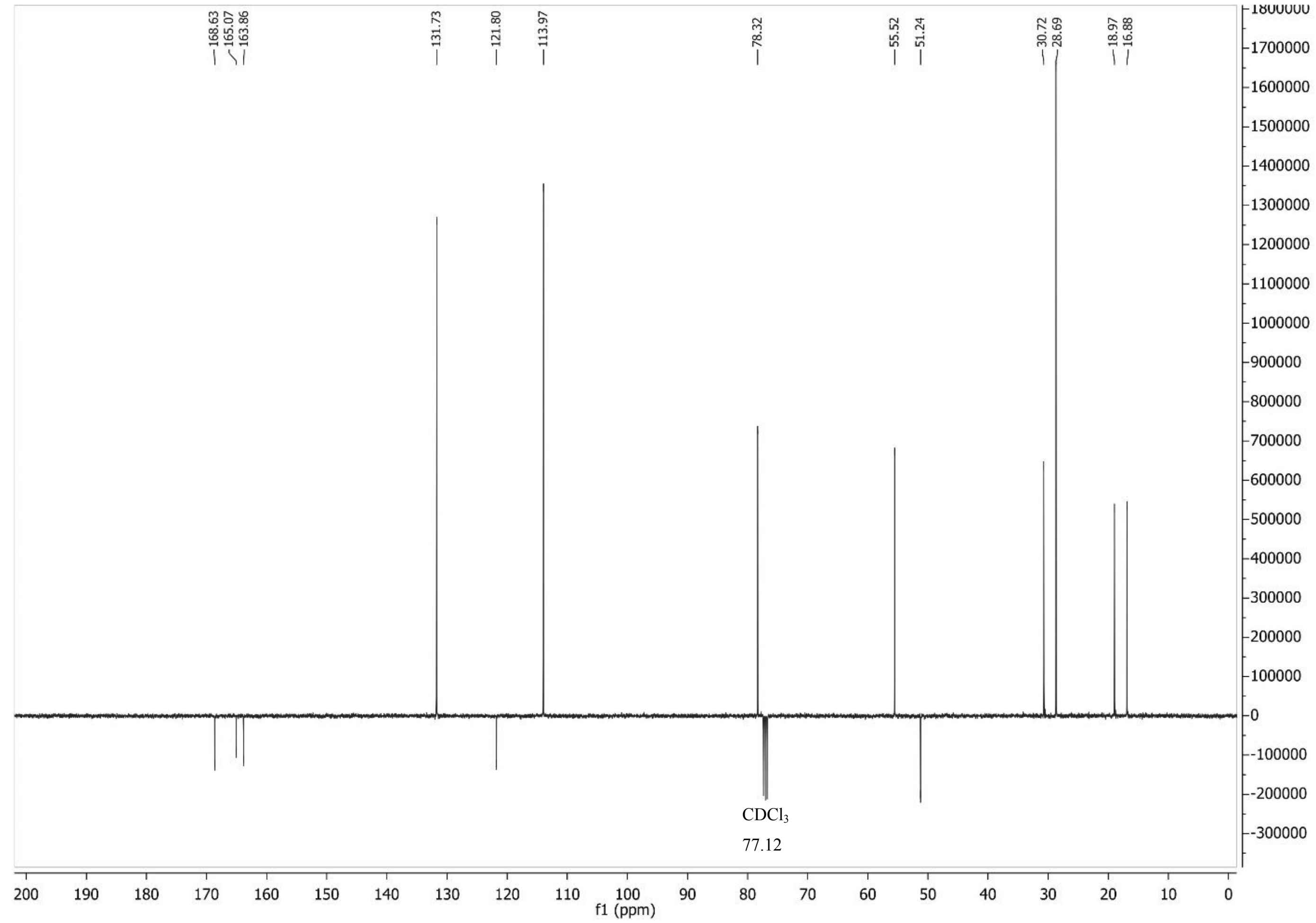

^{1}H and ^{13}C NMR spectra of compound **66**

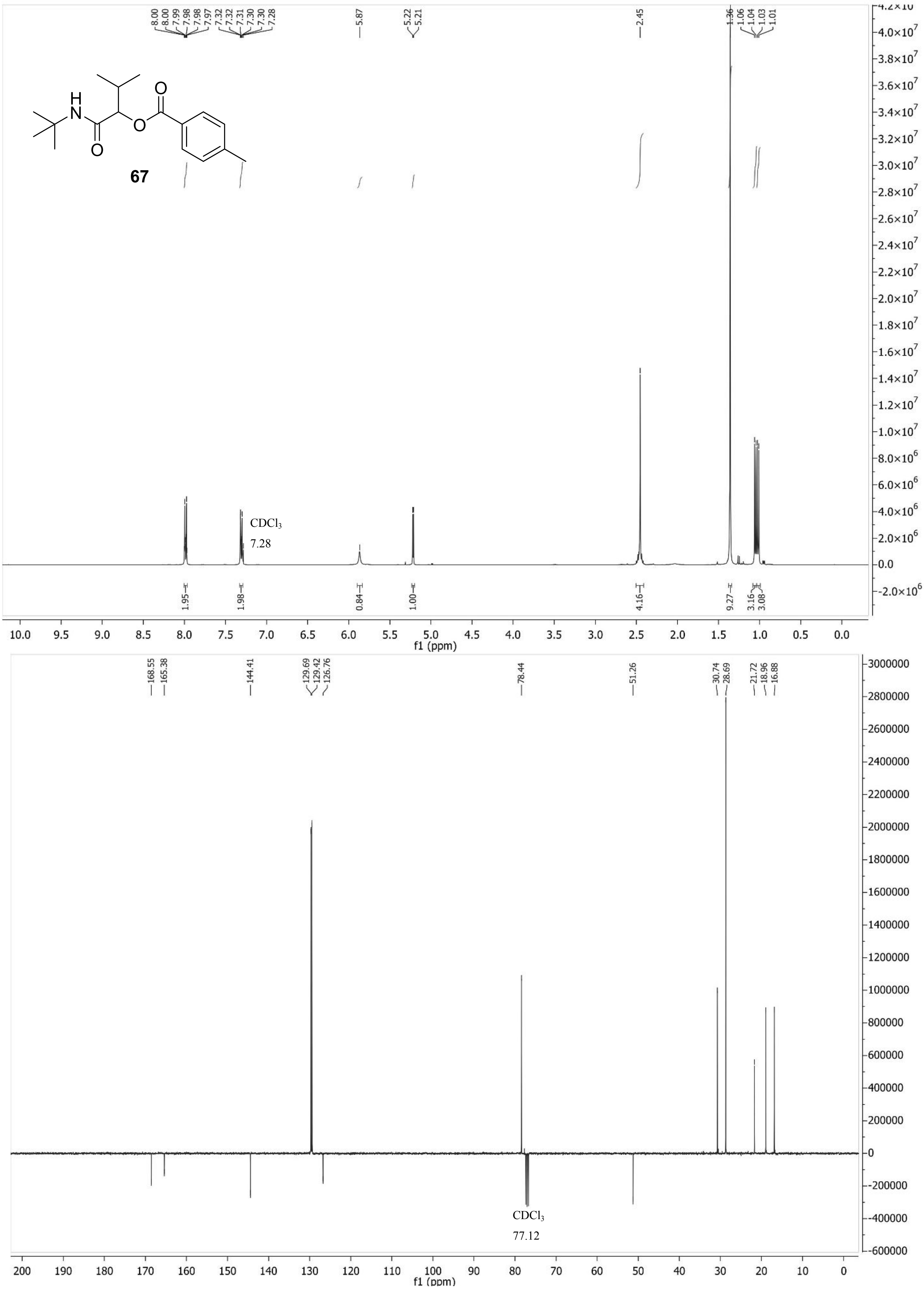

^{1}H and ^{13}C NMR spectra of compound **67**

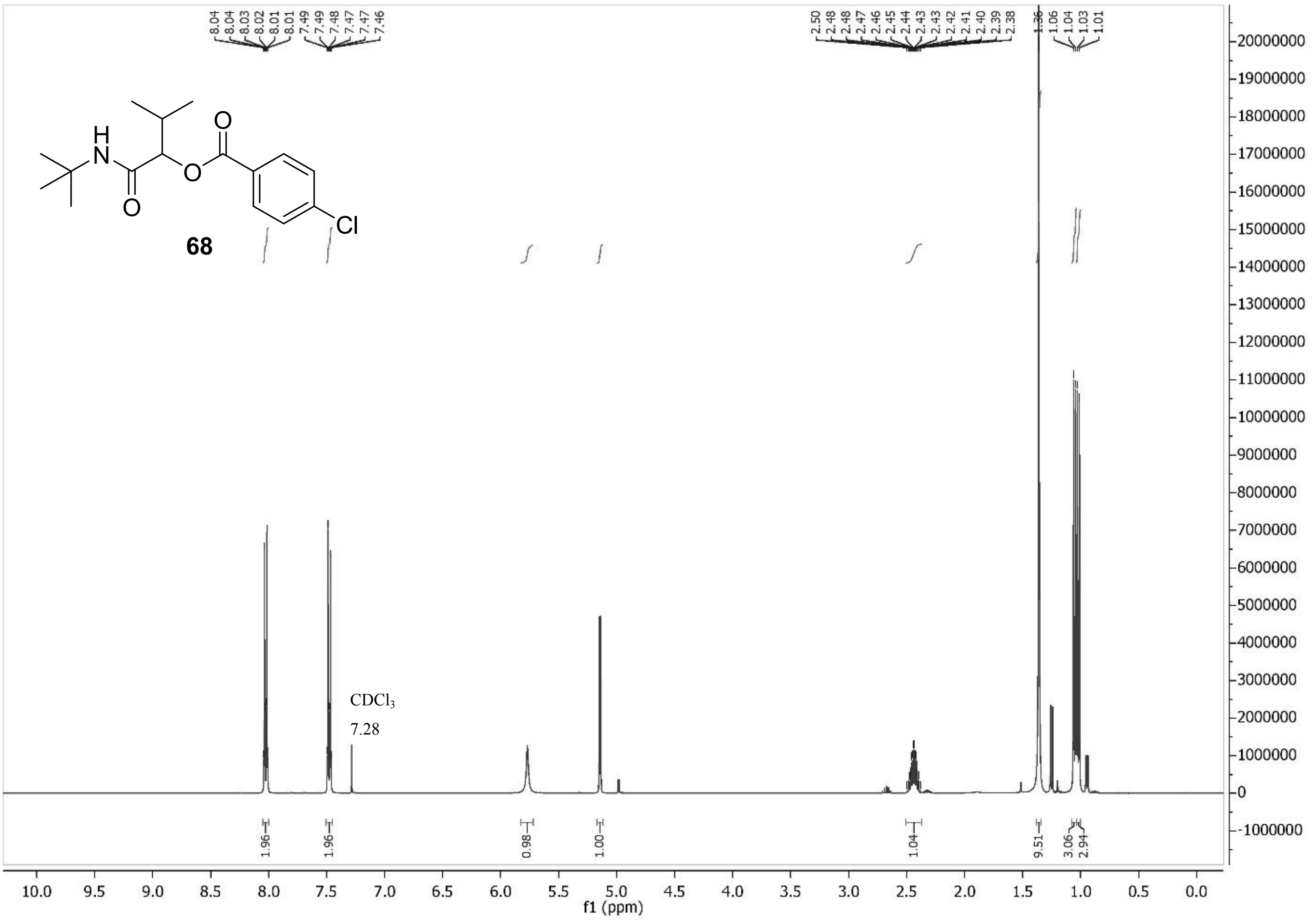

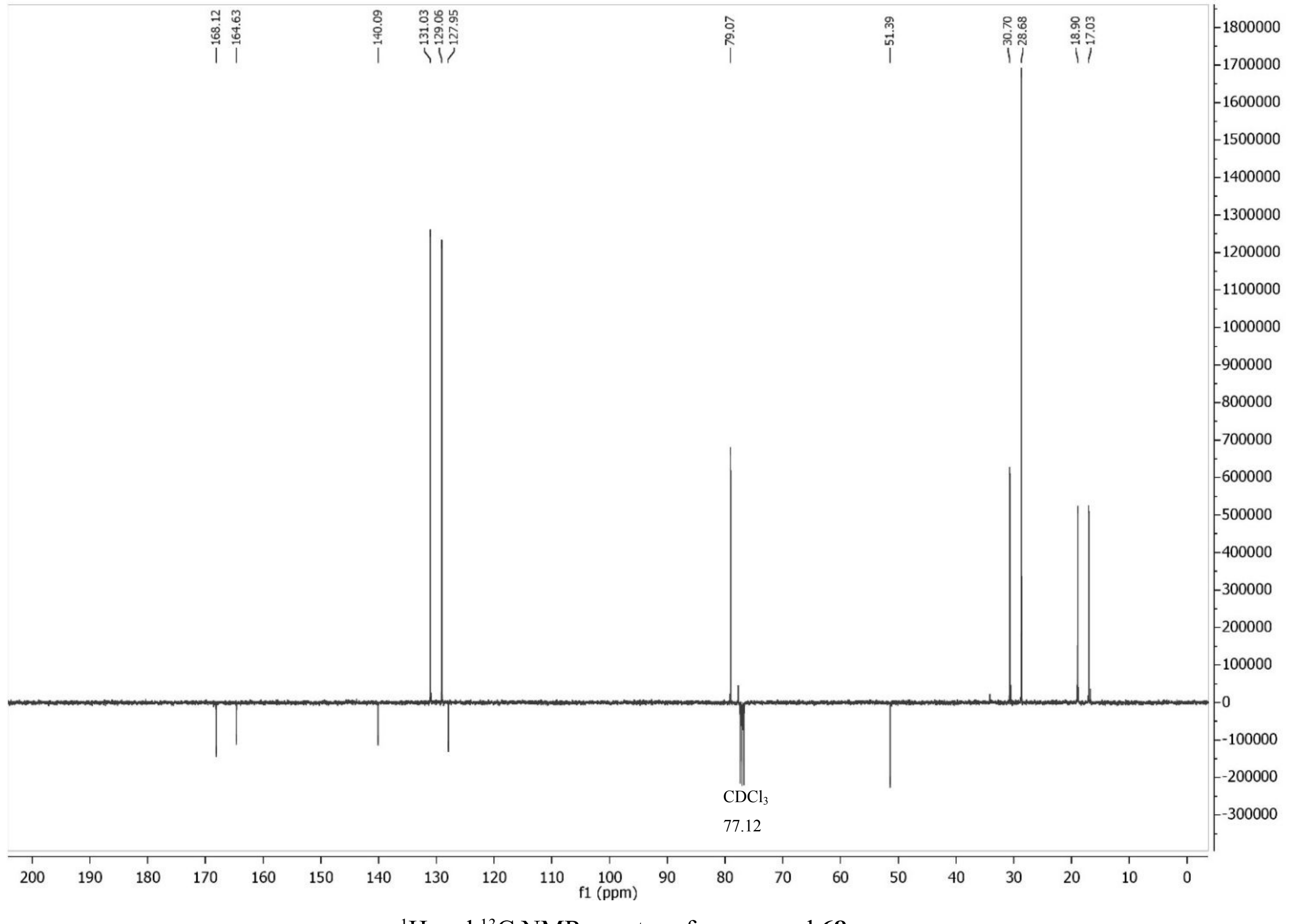

^{1}H and ^{13}C NMR spectra of compound **68**

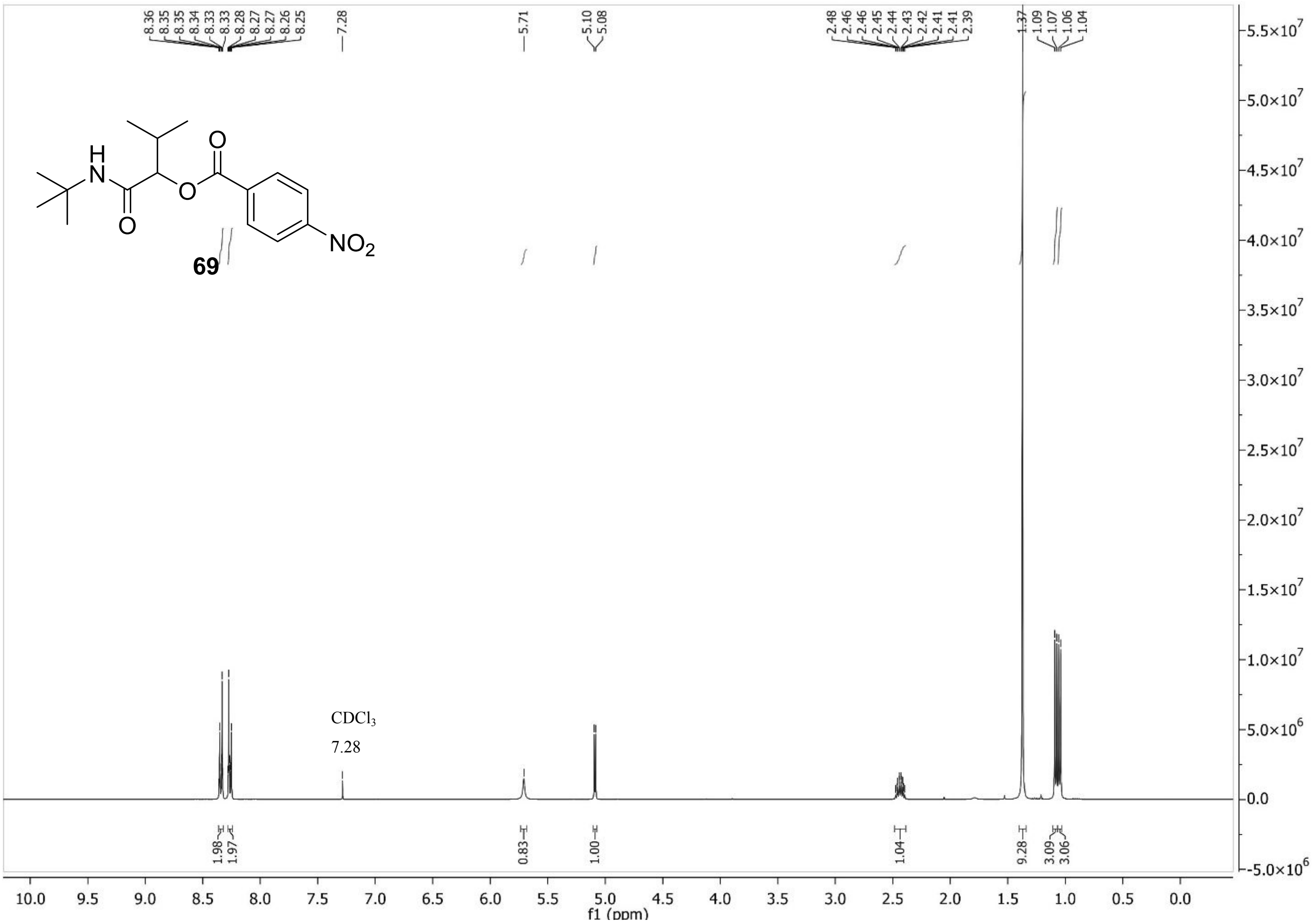

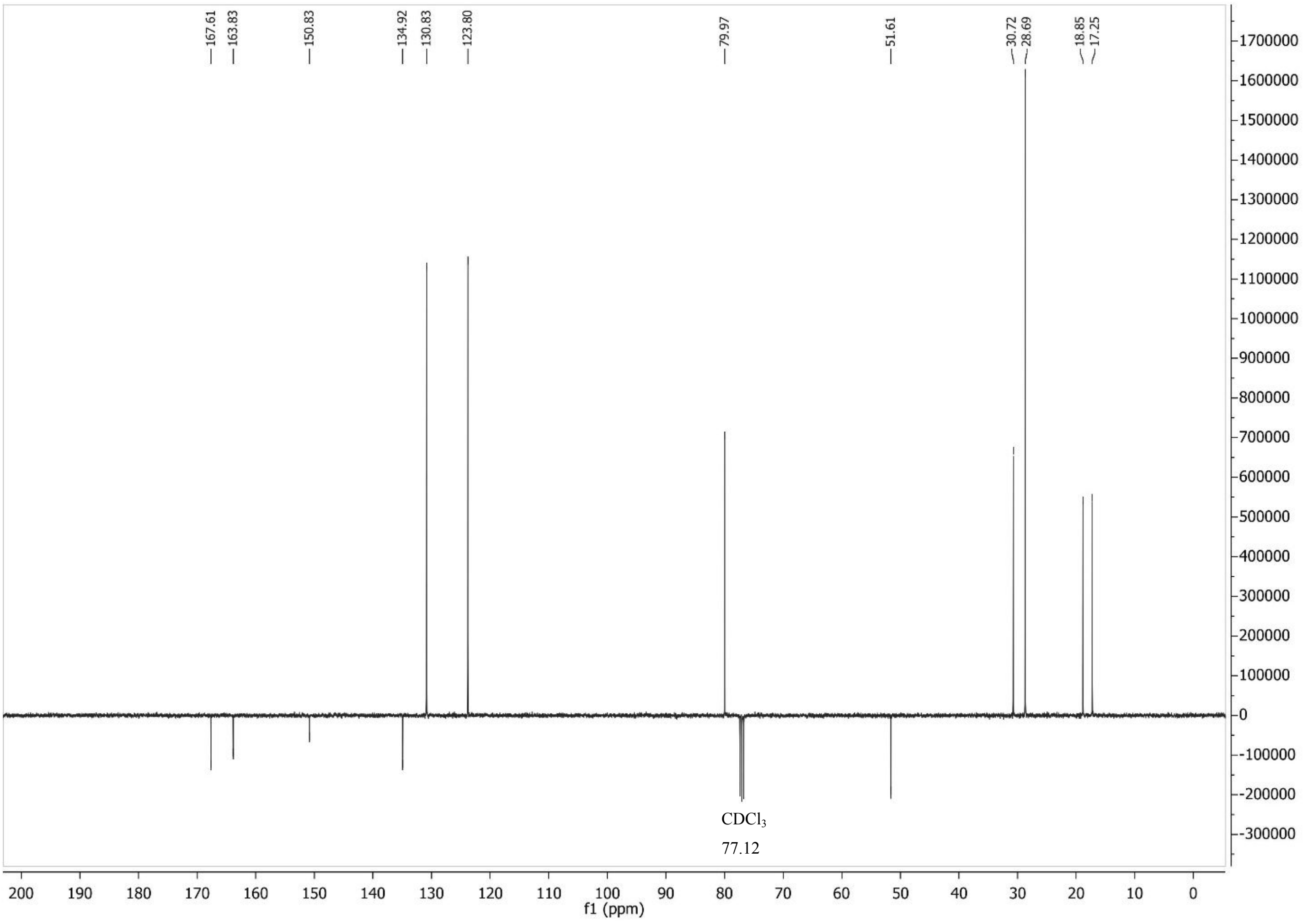

^{1}H and ^{13}C NMR spectra of compound **69**

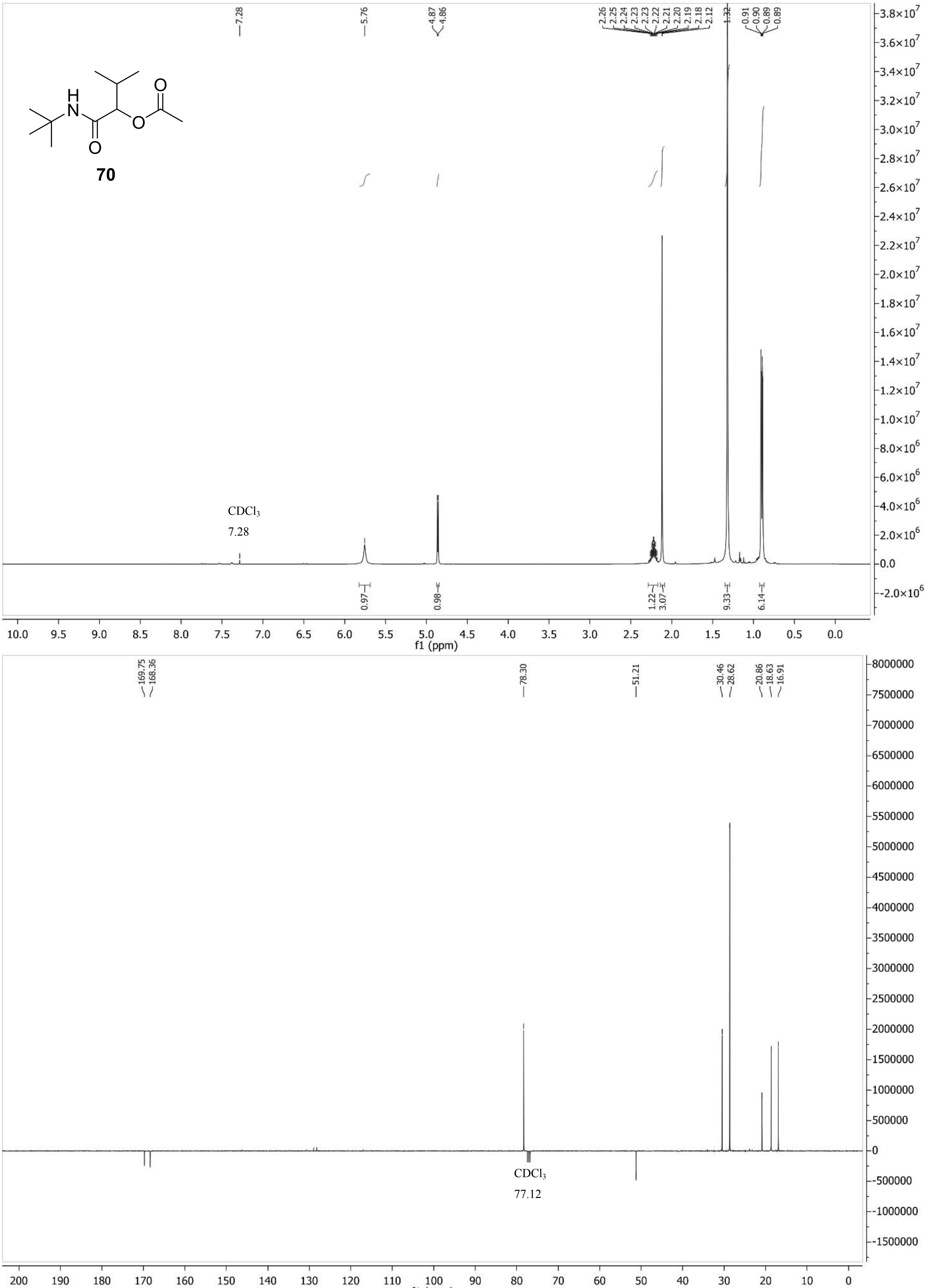

^{1}H and ^{13}C NMR spectra of compound **70**

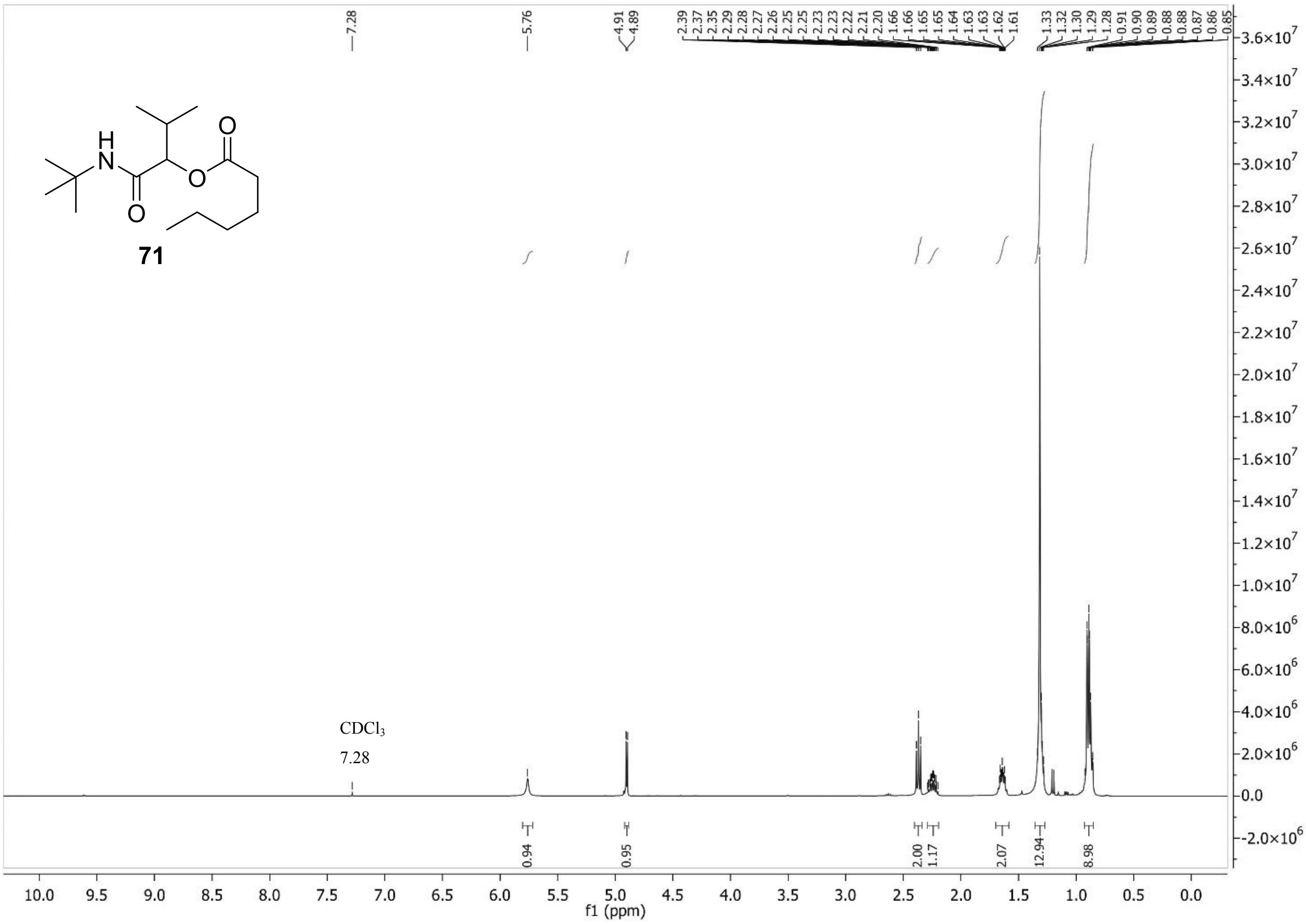

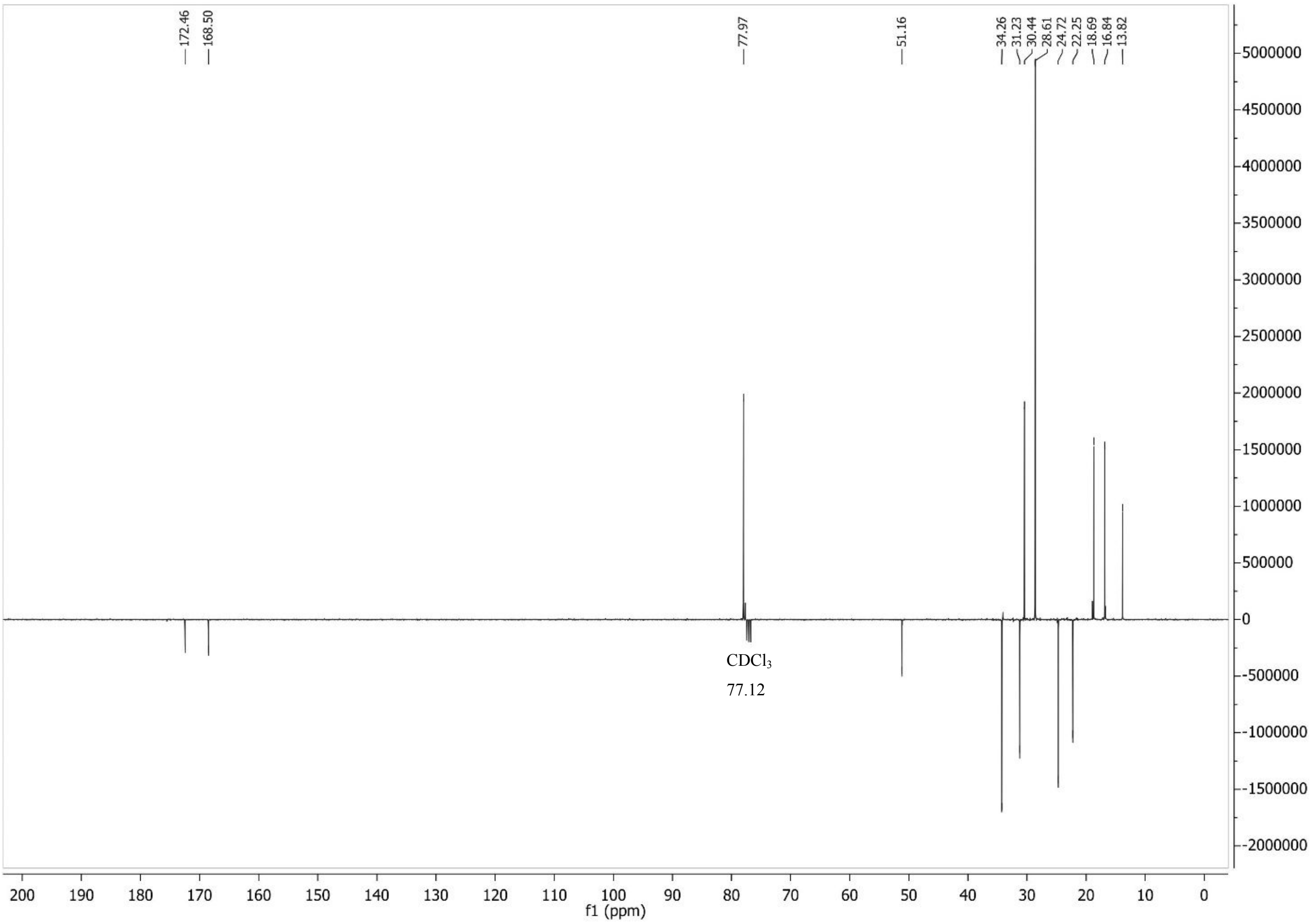

^{1}H and ^{13}C NMR spectra of compound **71**

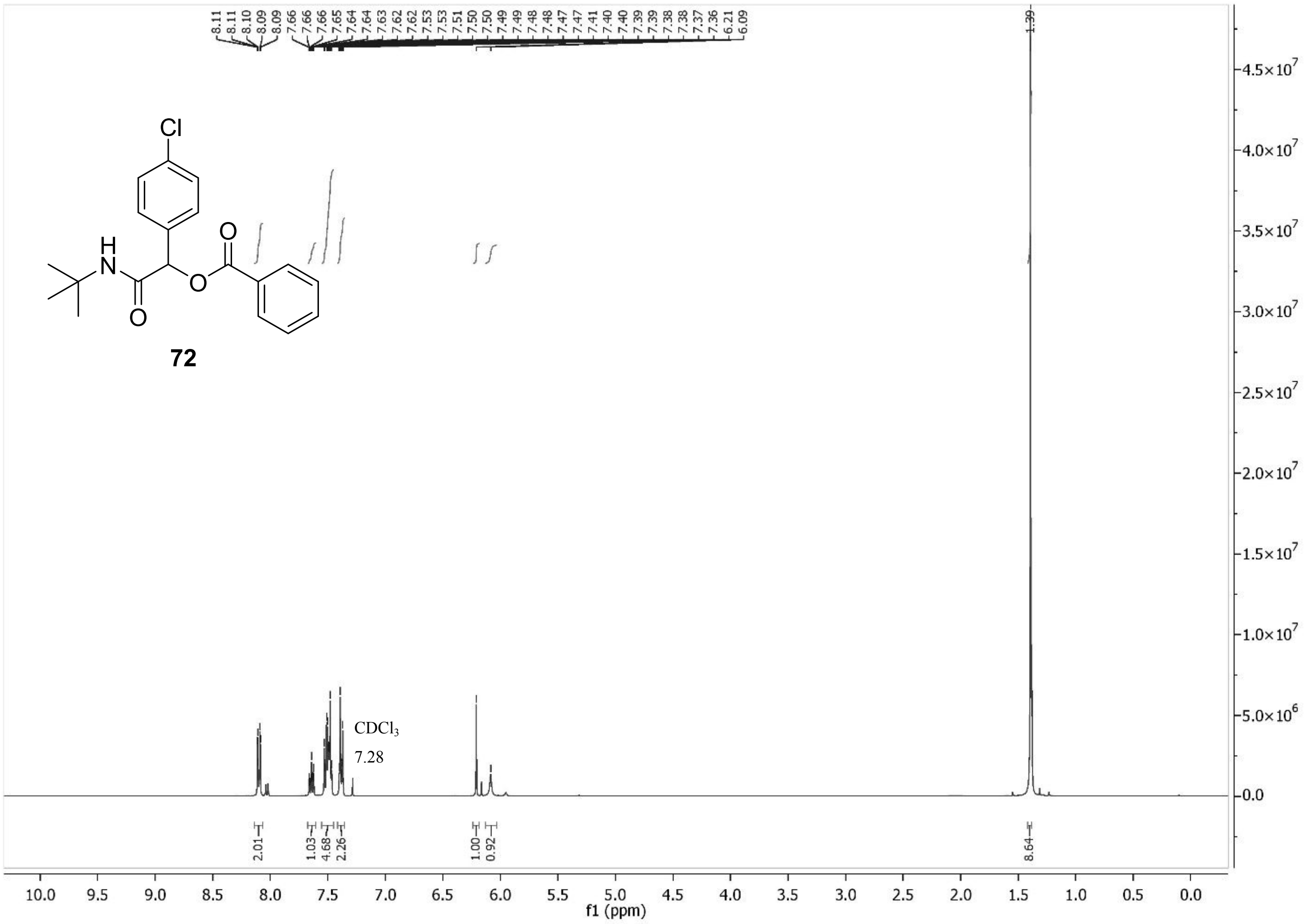

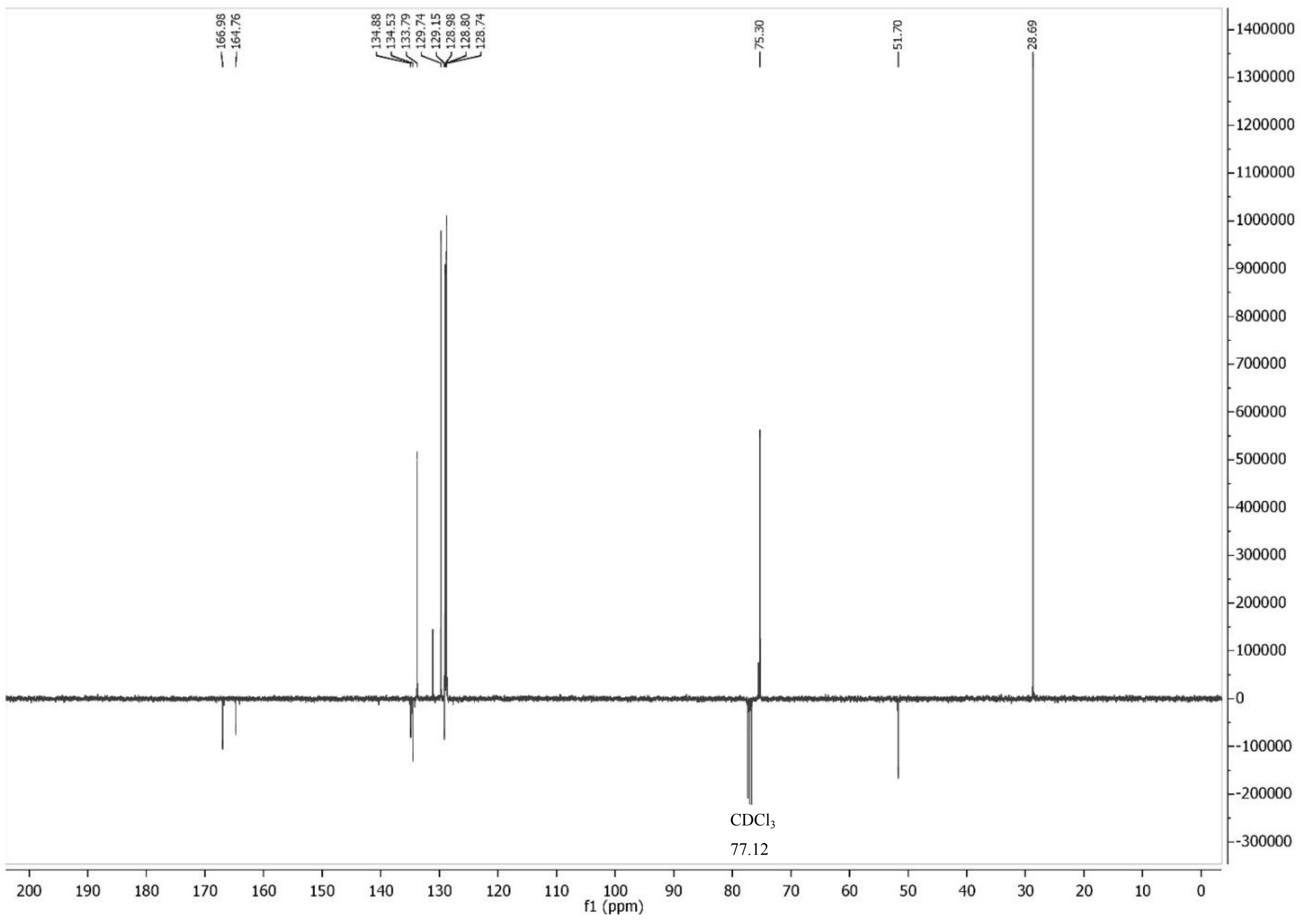

^{1}H and ^{13}C NMR spectra of compound **72**

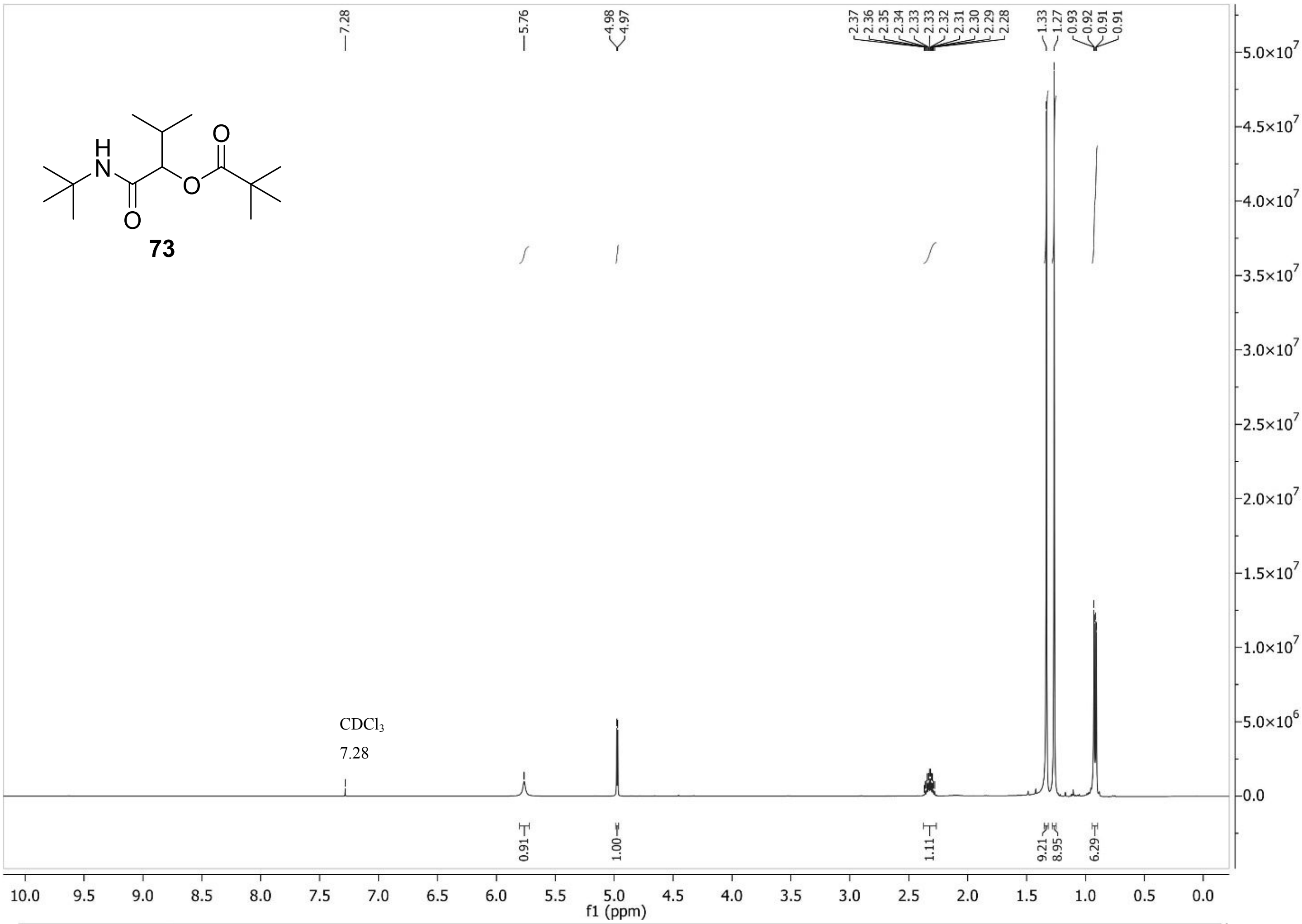

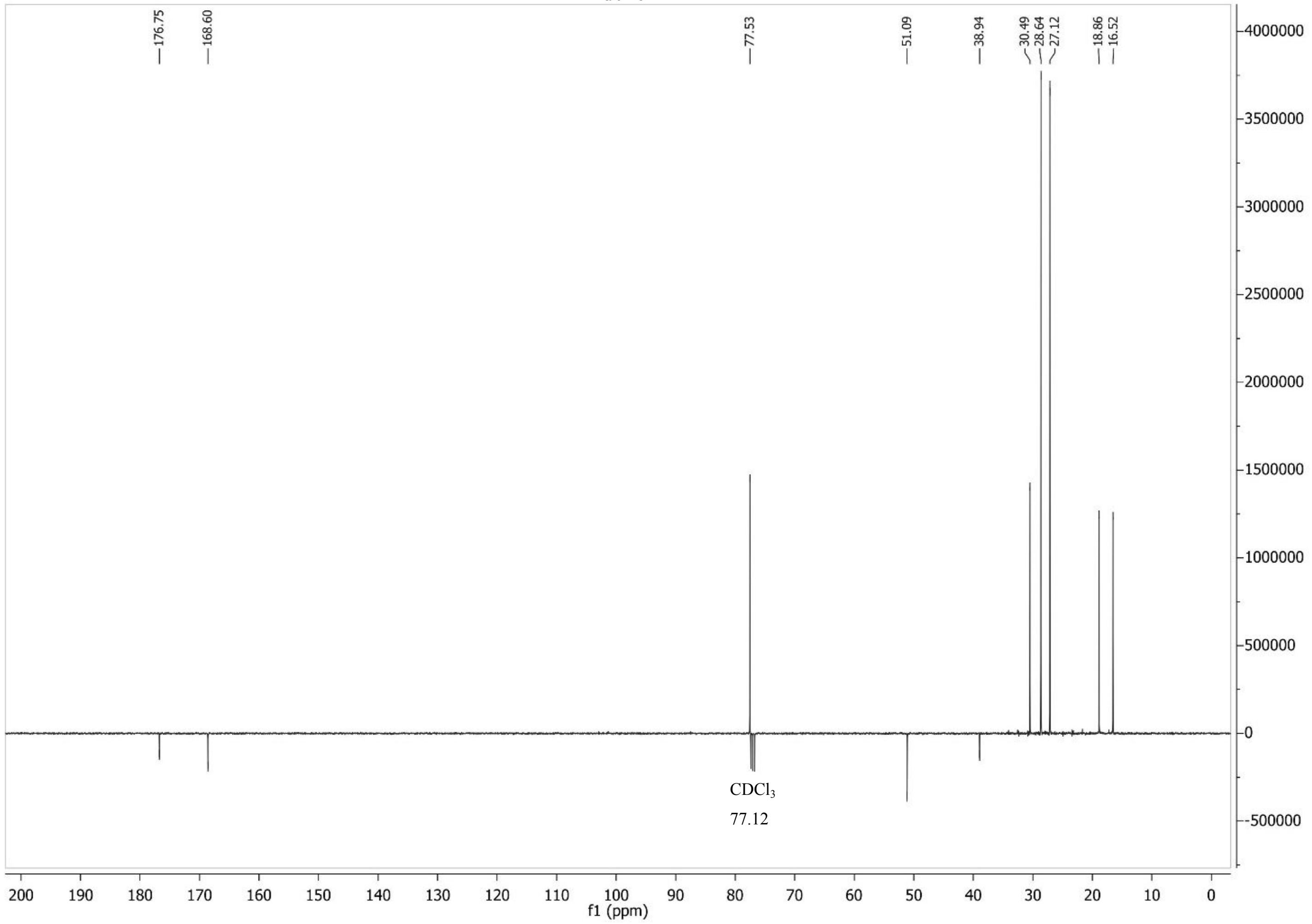

^{1}H and ^{13}C NMR spectra of compound **73**

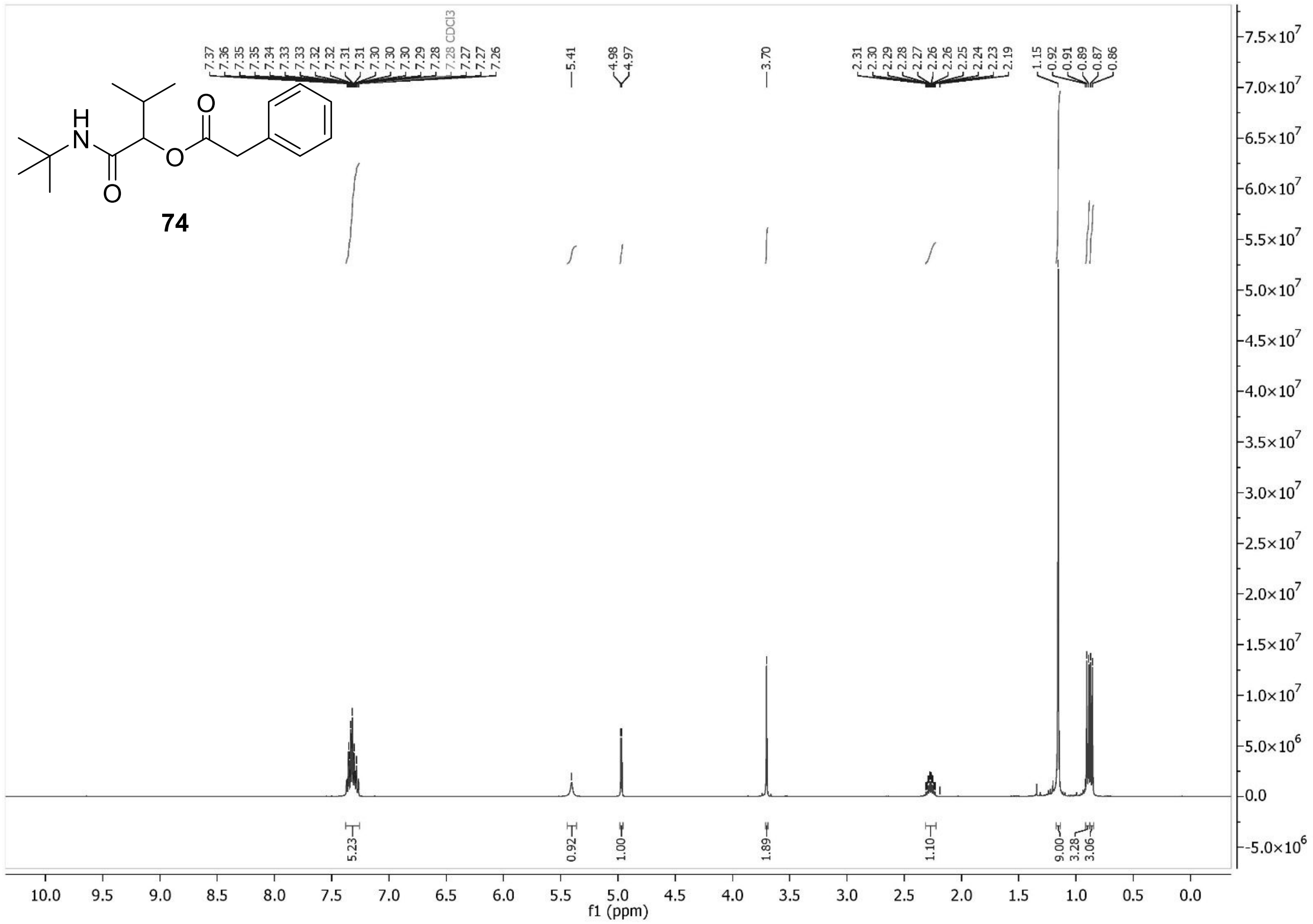

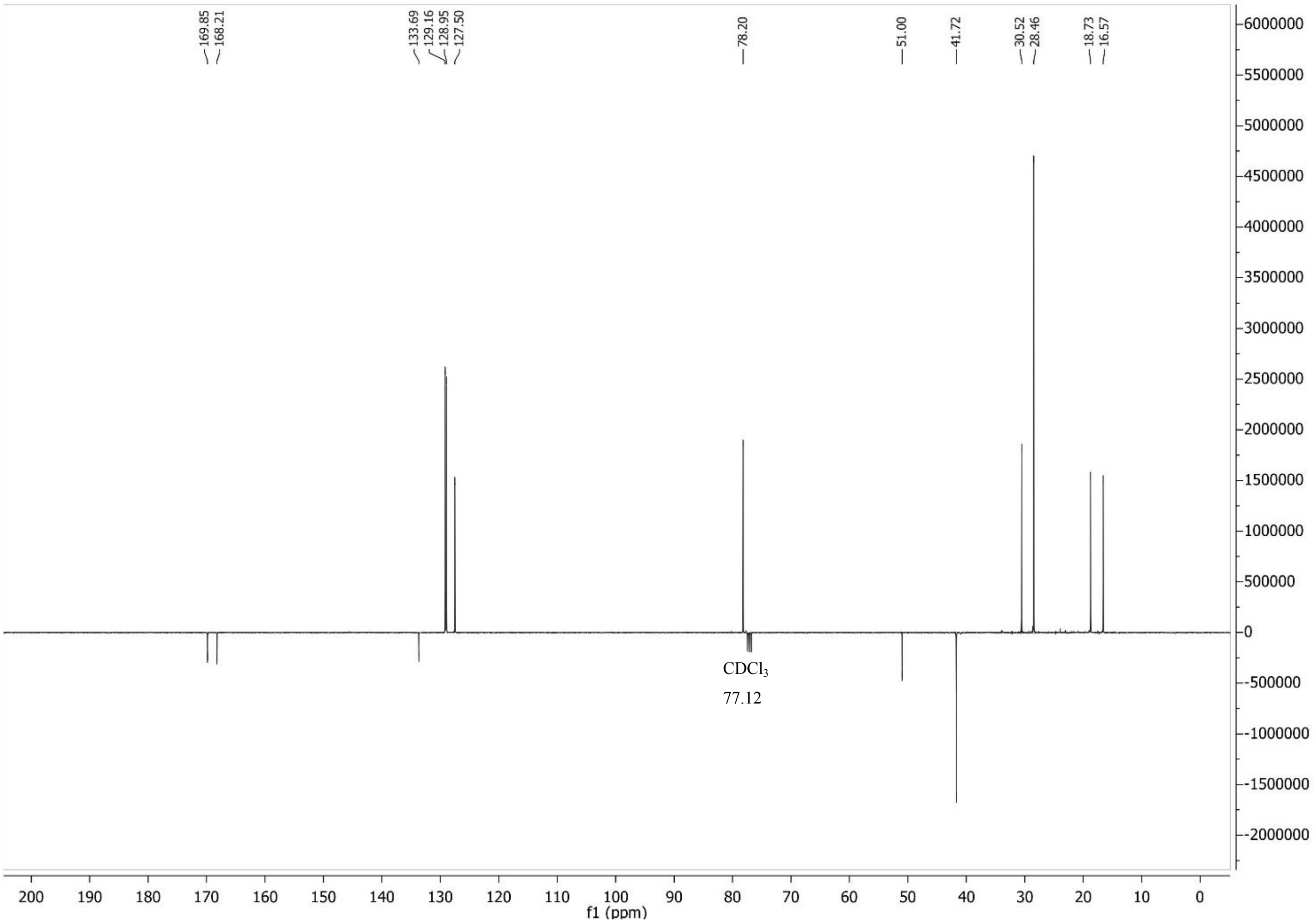

^{1}H and ^{13}C NMR spectra of compound **74**

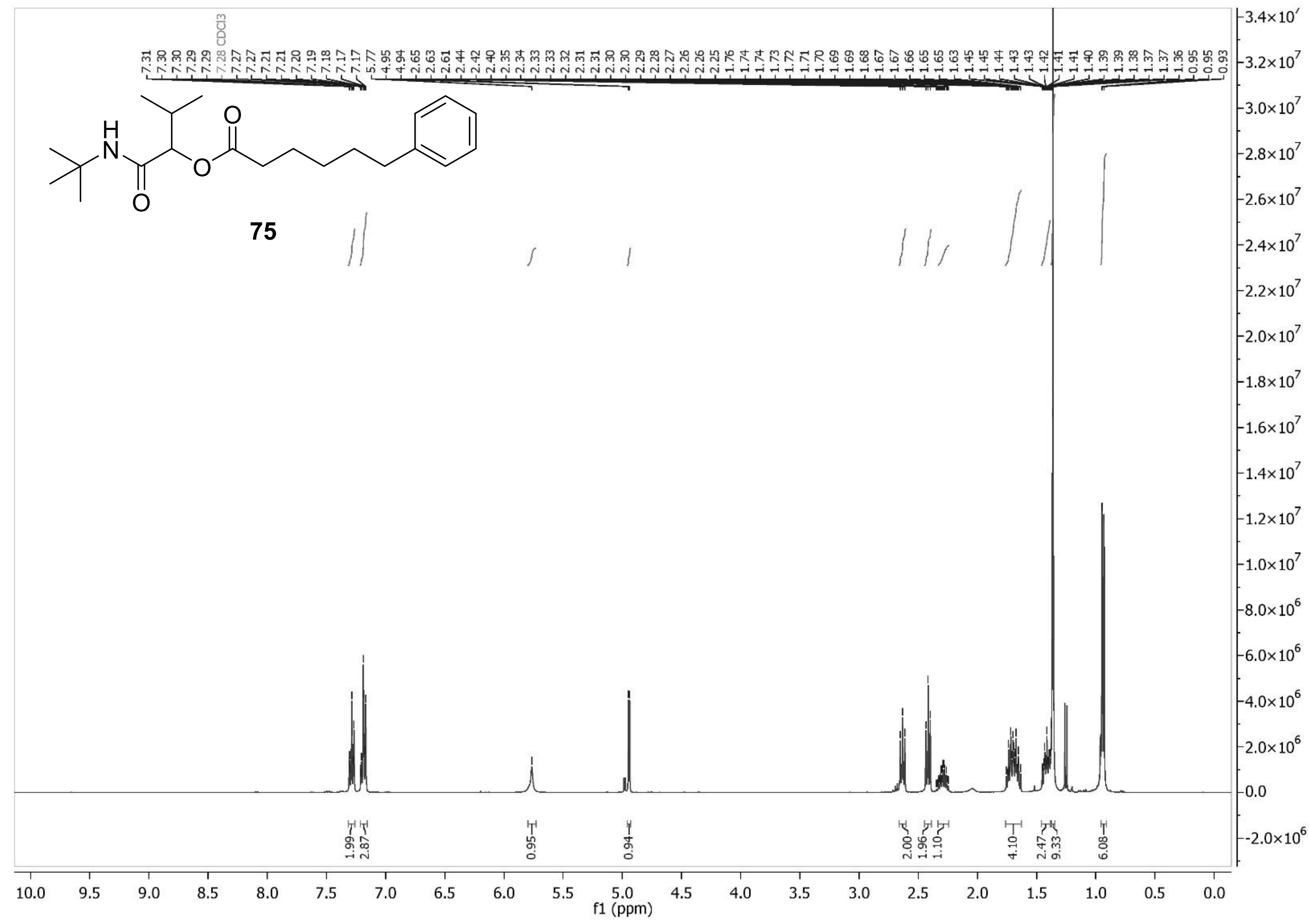

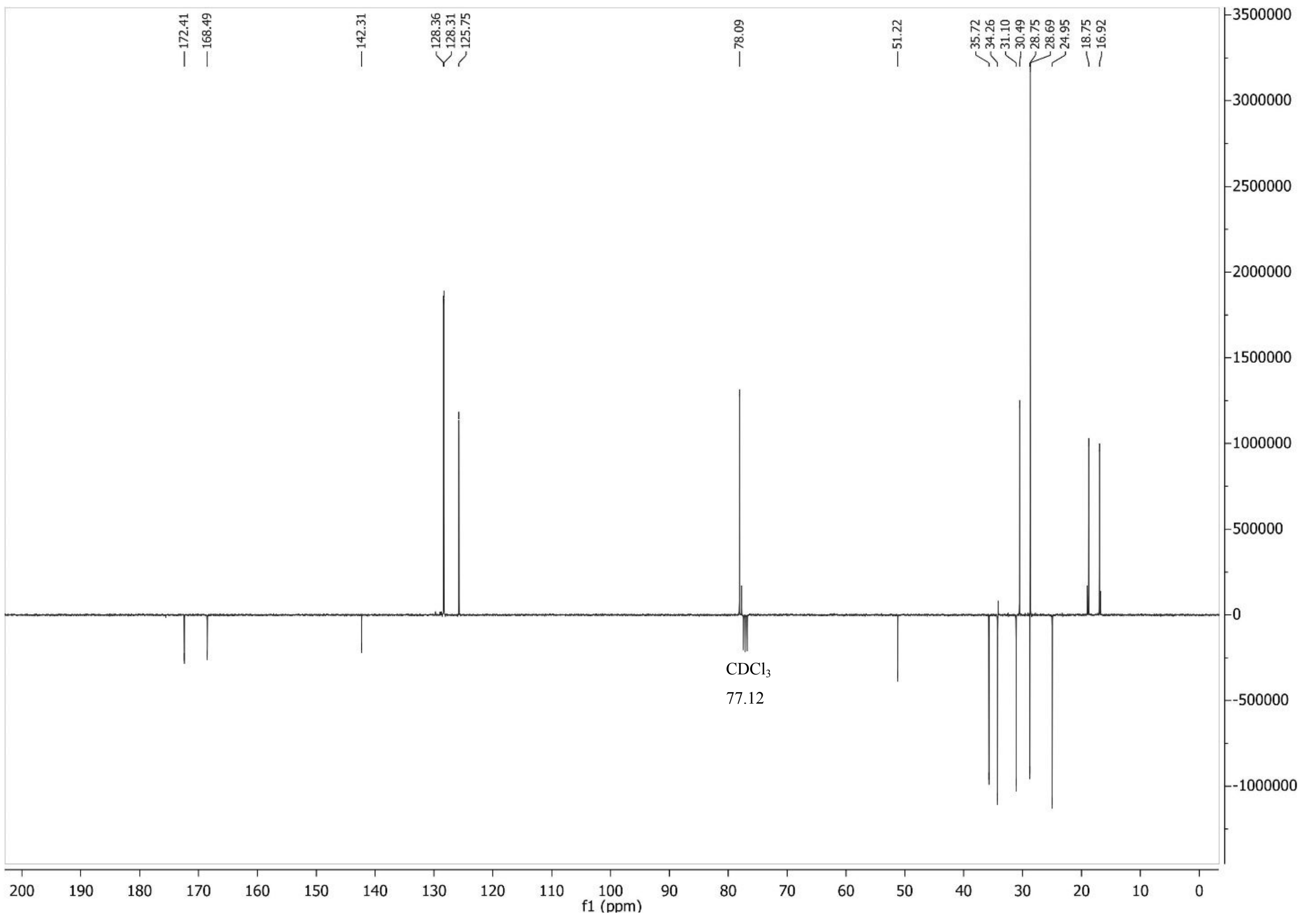

^{1}H and ^{13}C NMR spectra of compound **75**

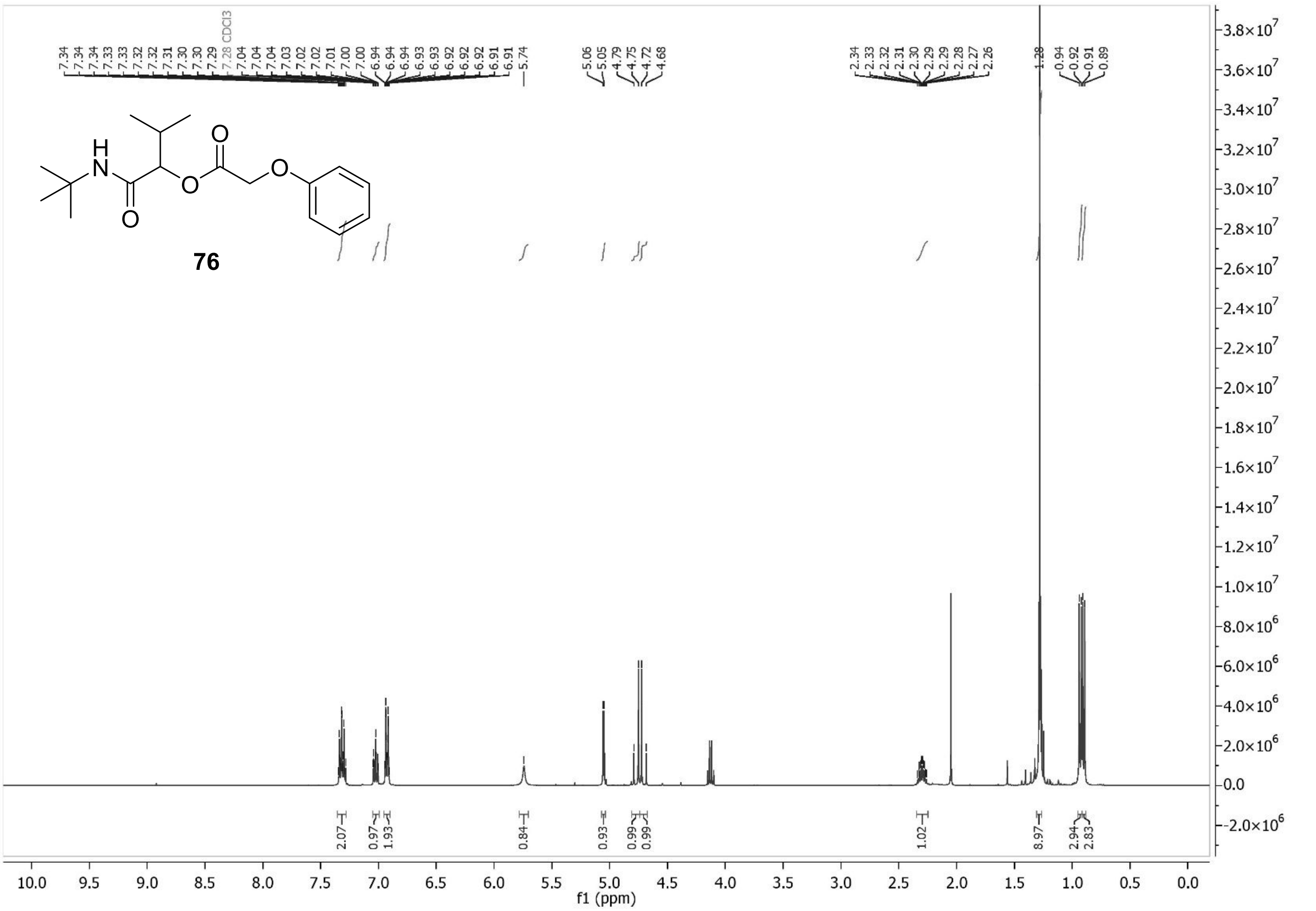

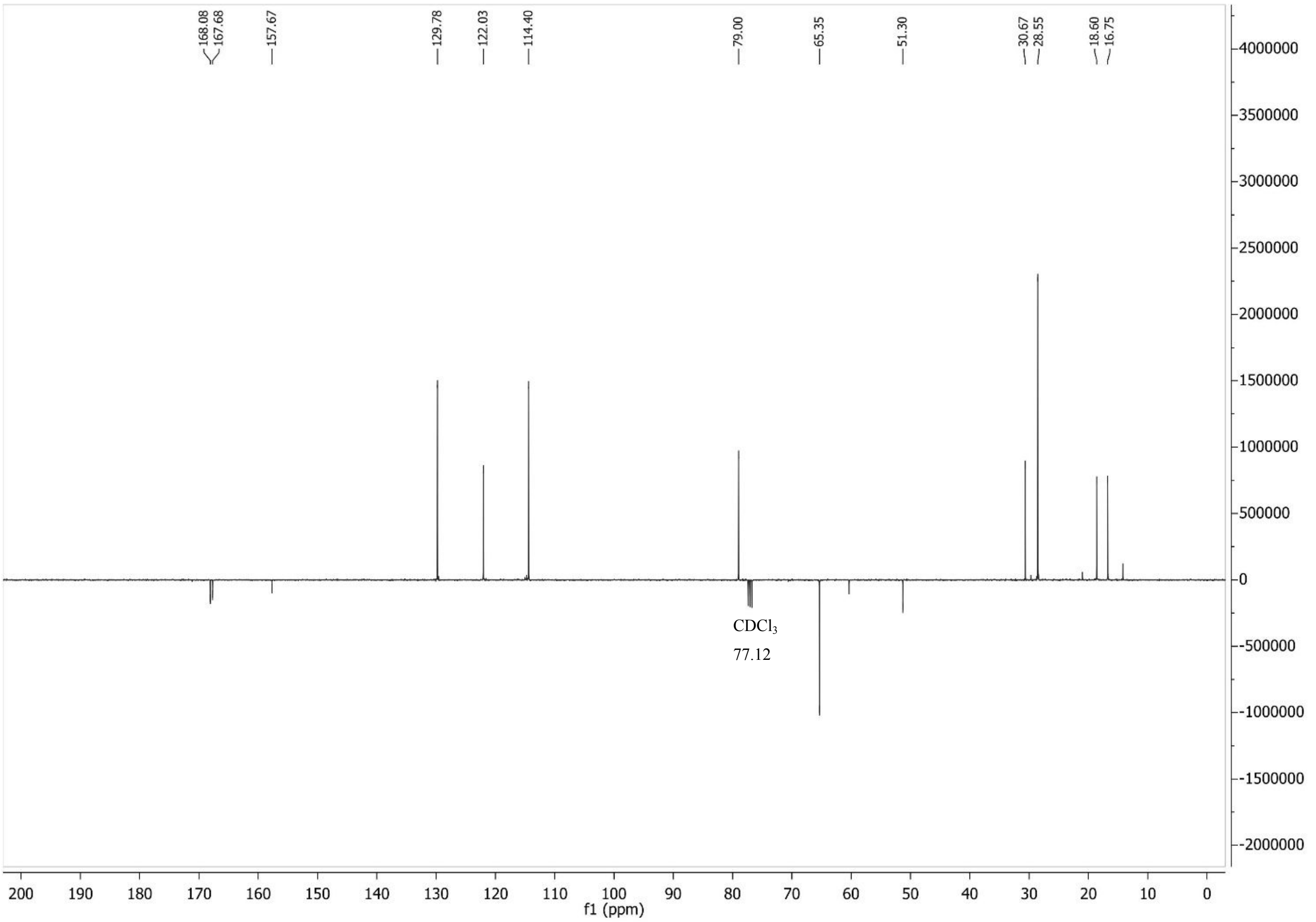

^{1}H and ^{13}C NMR spectra of compound **76**

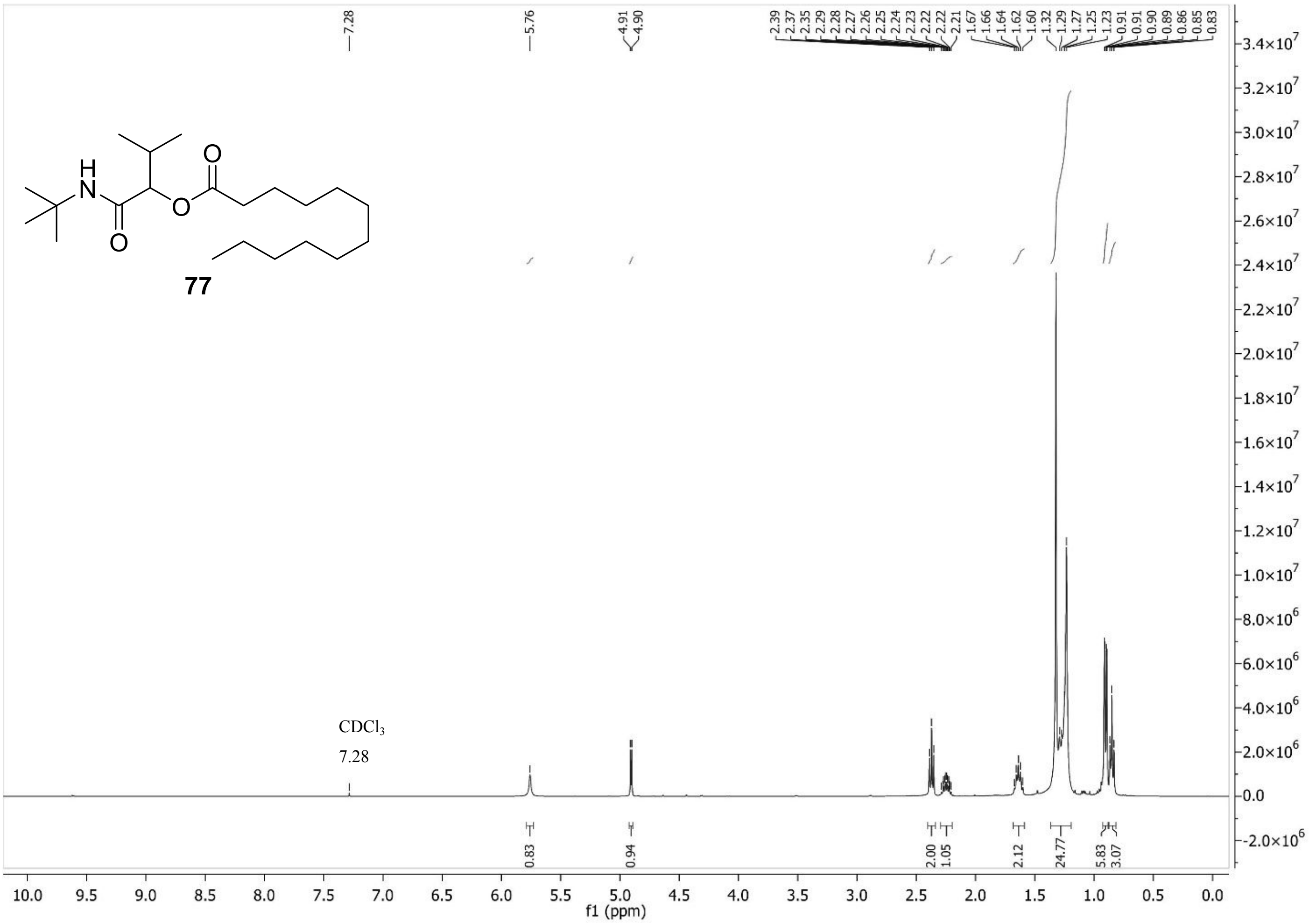

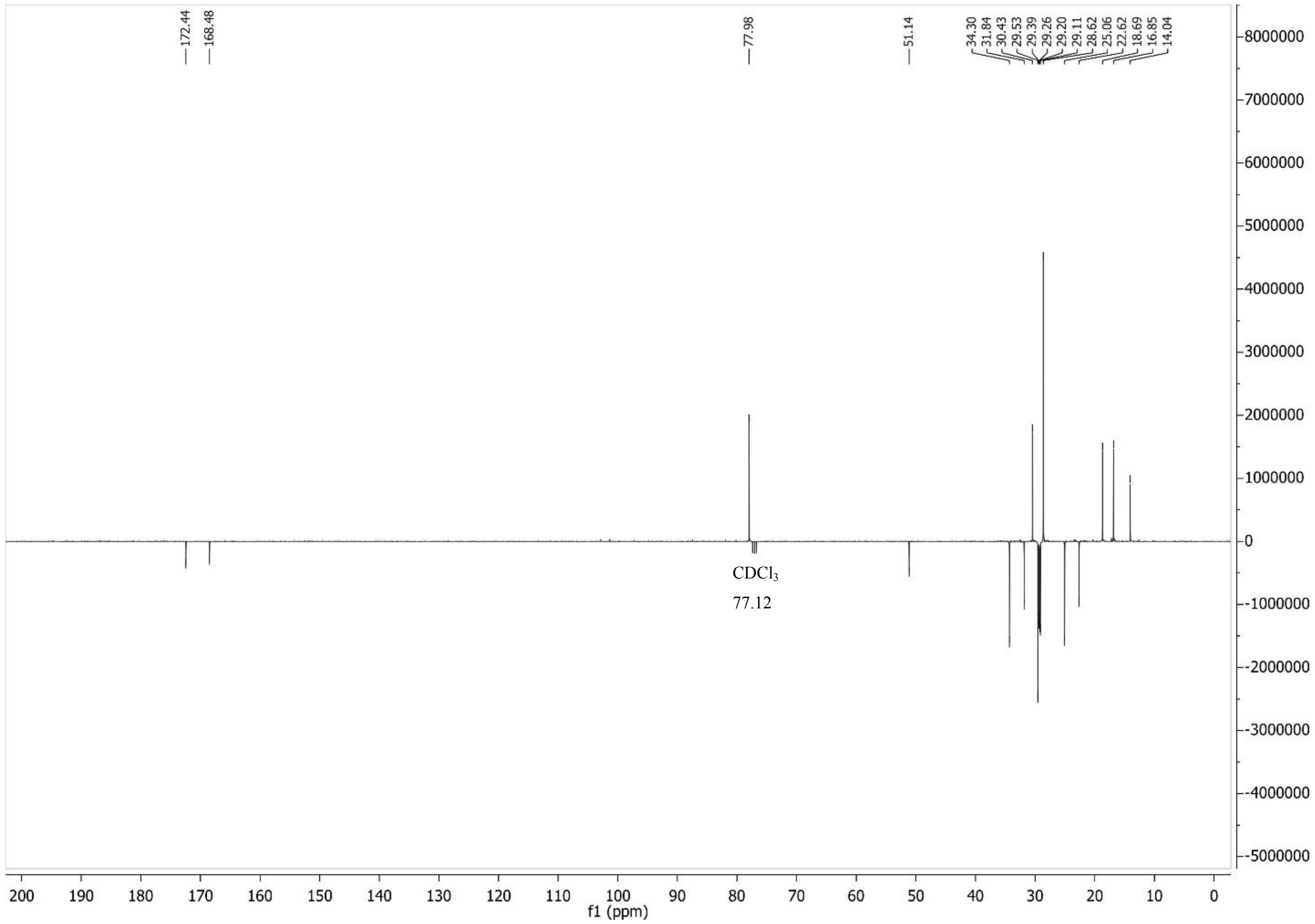

^{1}H and ^{13}C NMR spectra of compound **77**

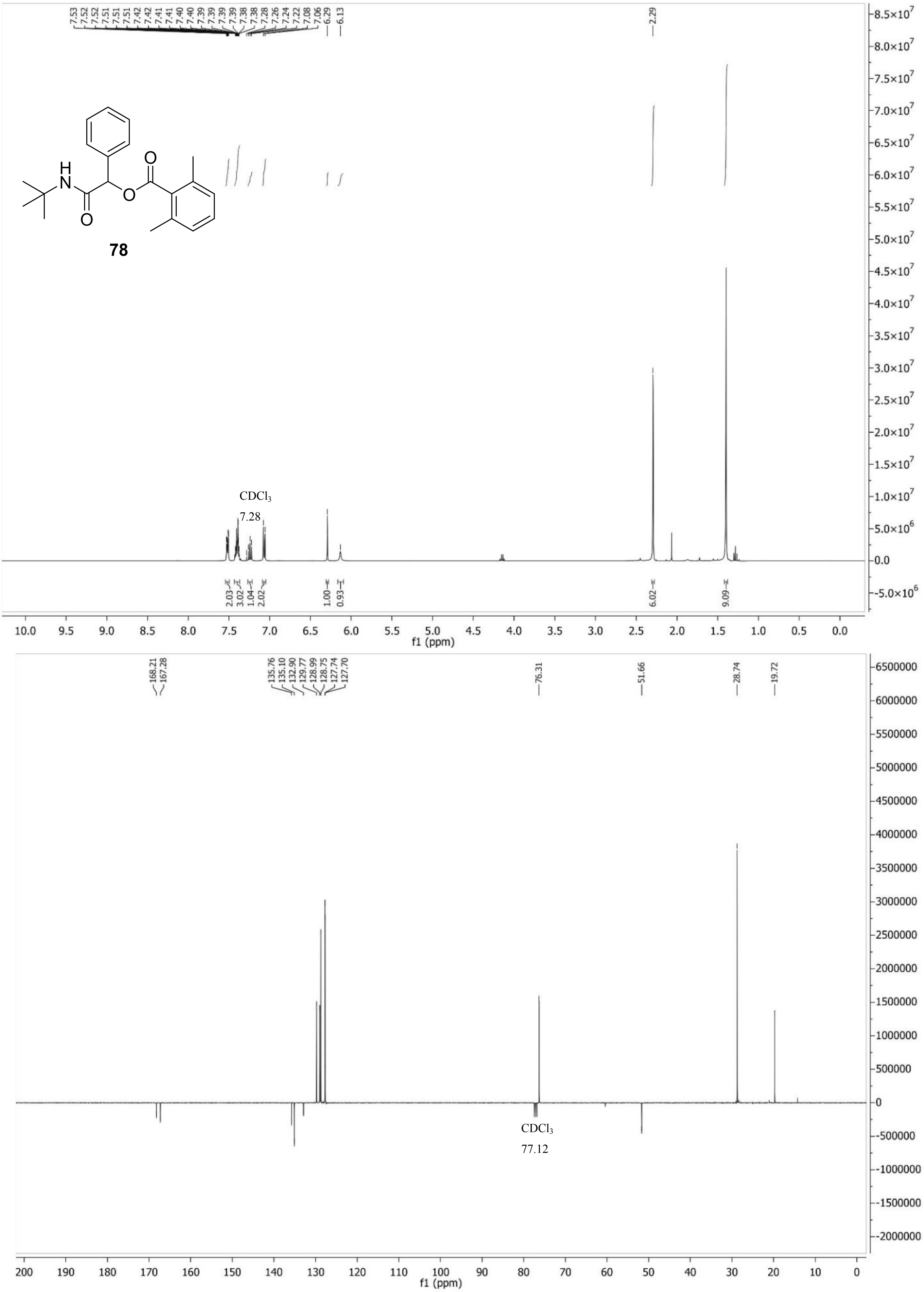

^{1}H and ^{13}C NMR spectra of compound **78**

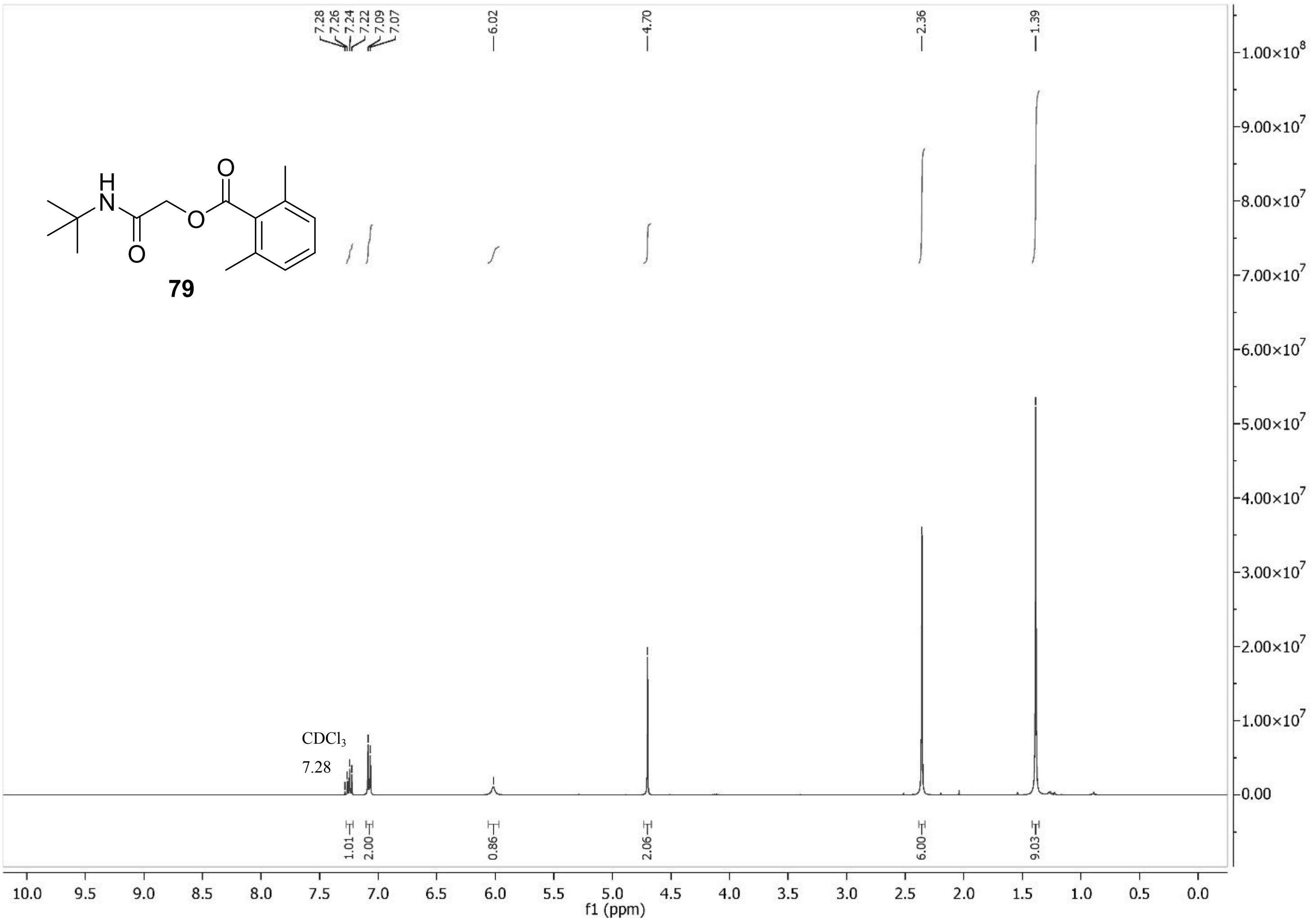

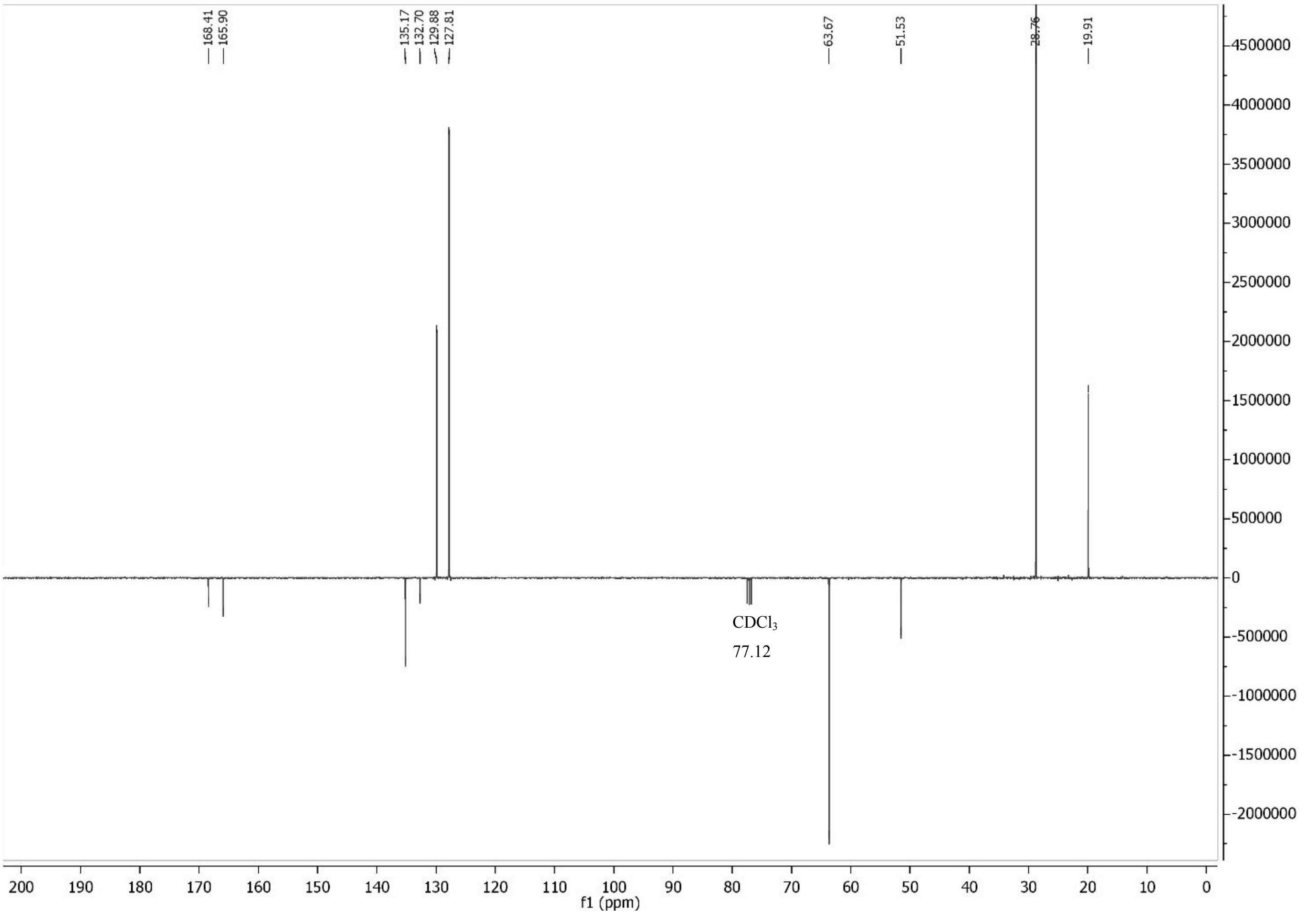

^{1}H and ^{13}C NMR spectra of compound **79**

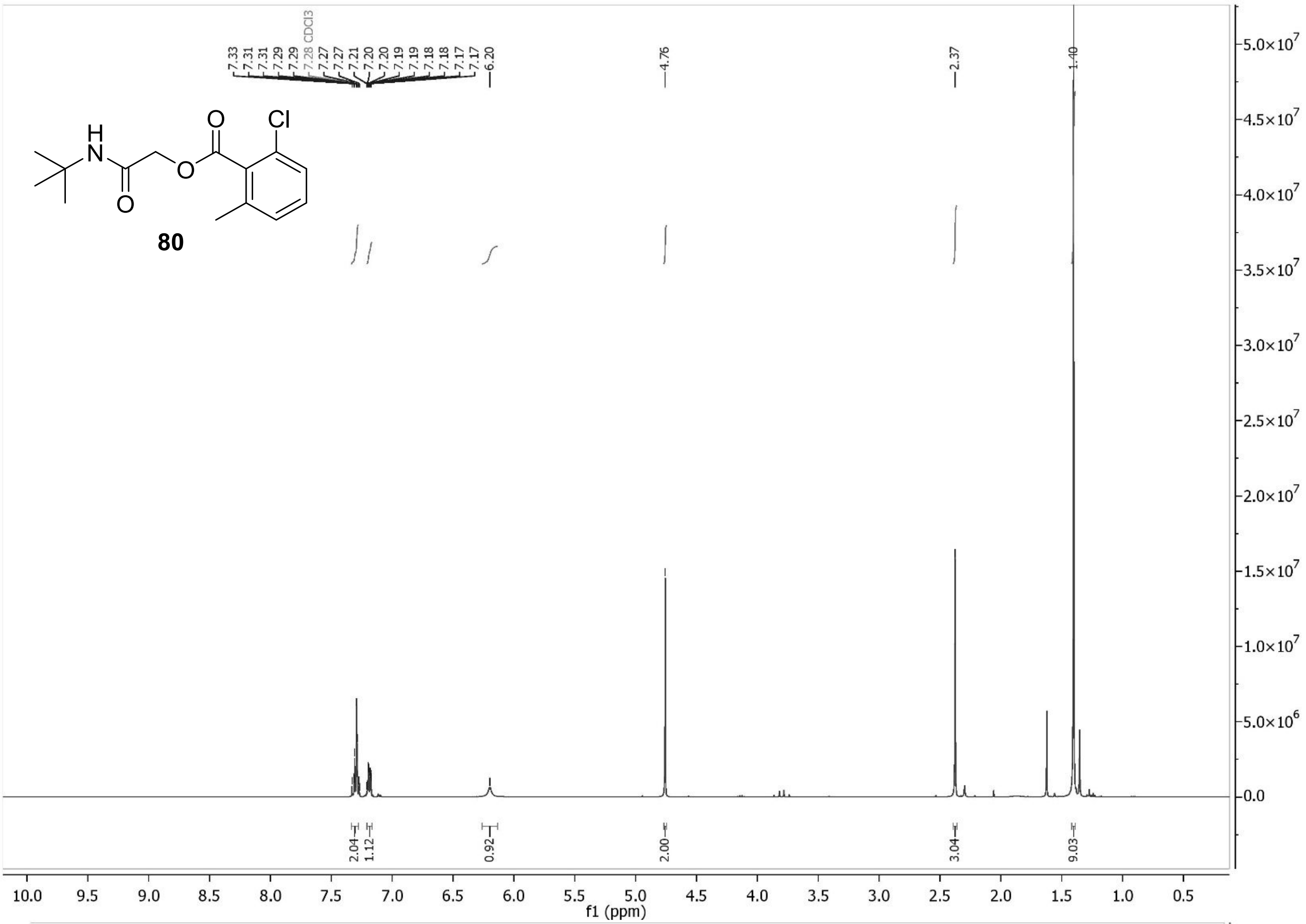

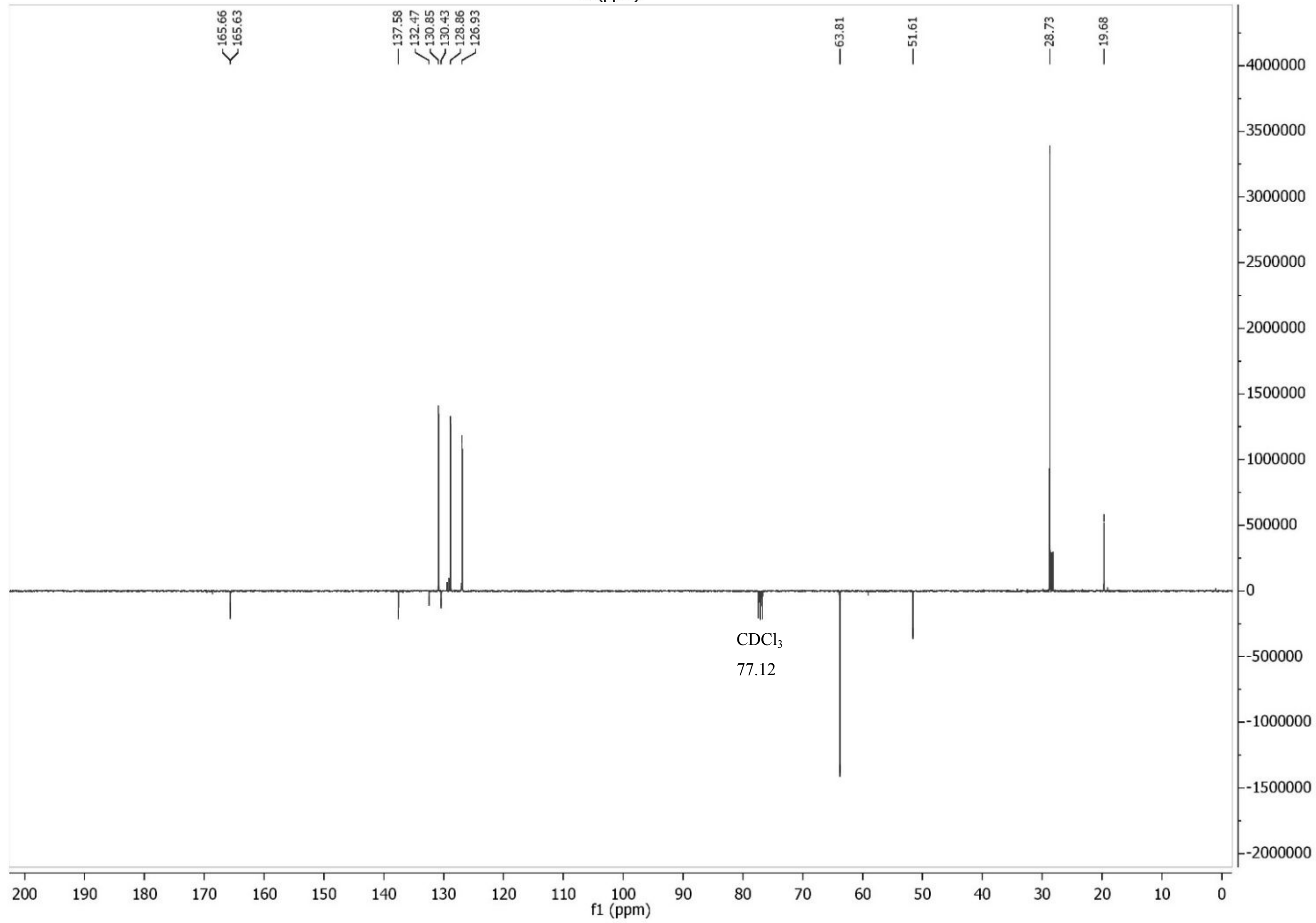

^{1}H and ^{13}C NMR spectra of compound **80**

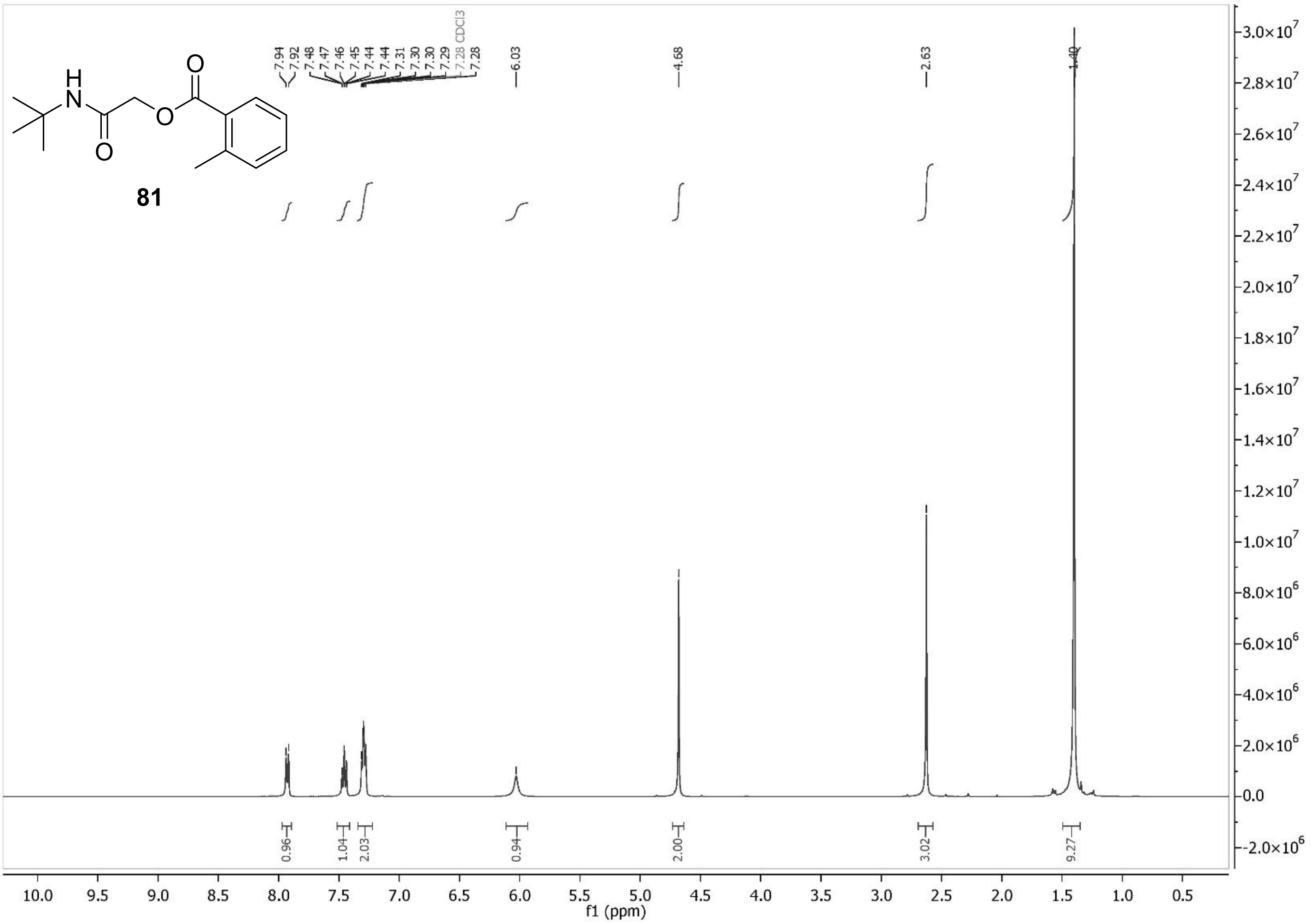

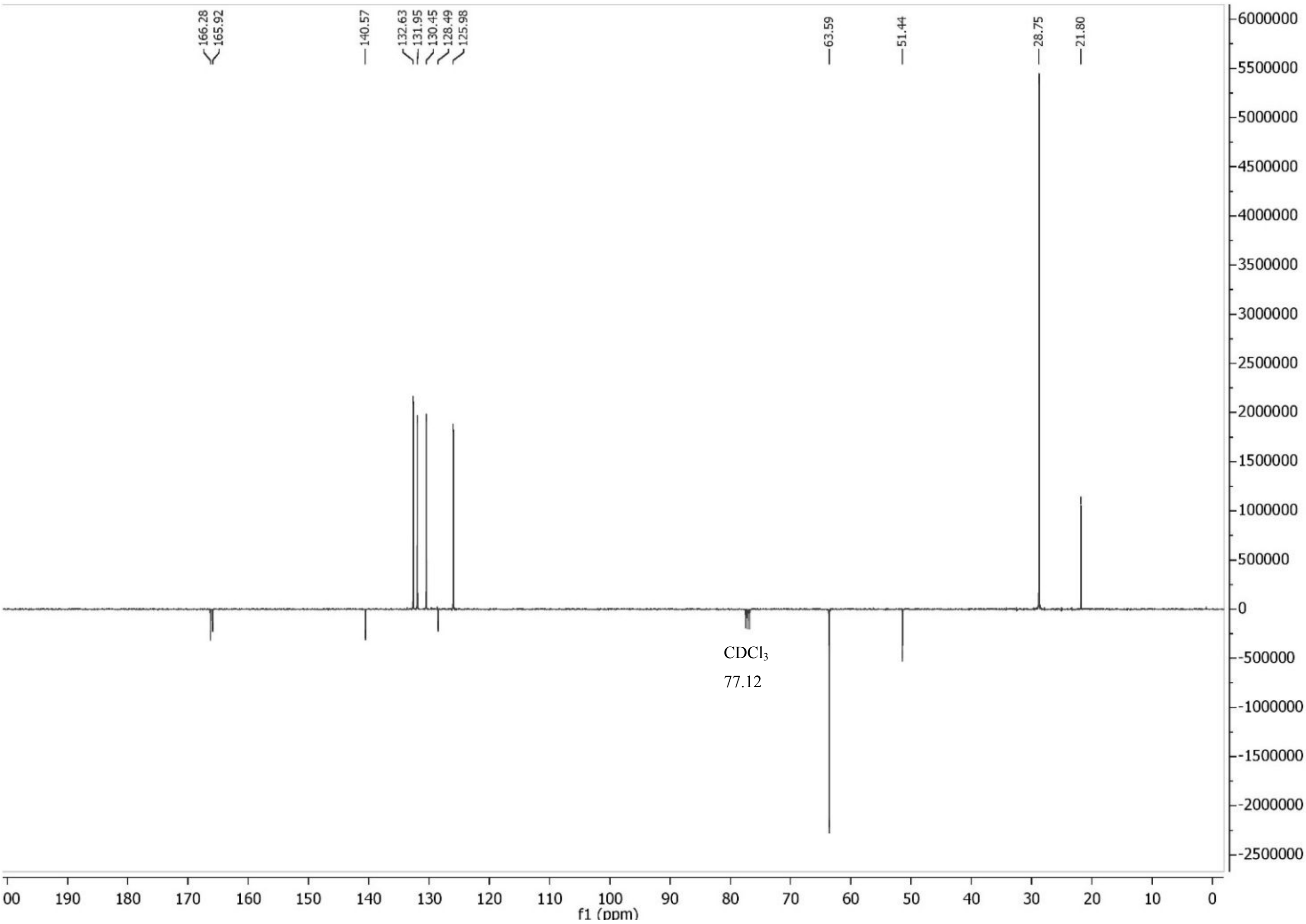

^{1}H and ^{13}C NMR spectra of compound **81**

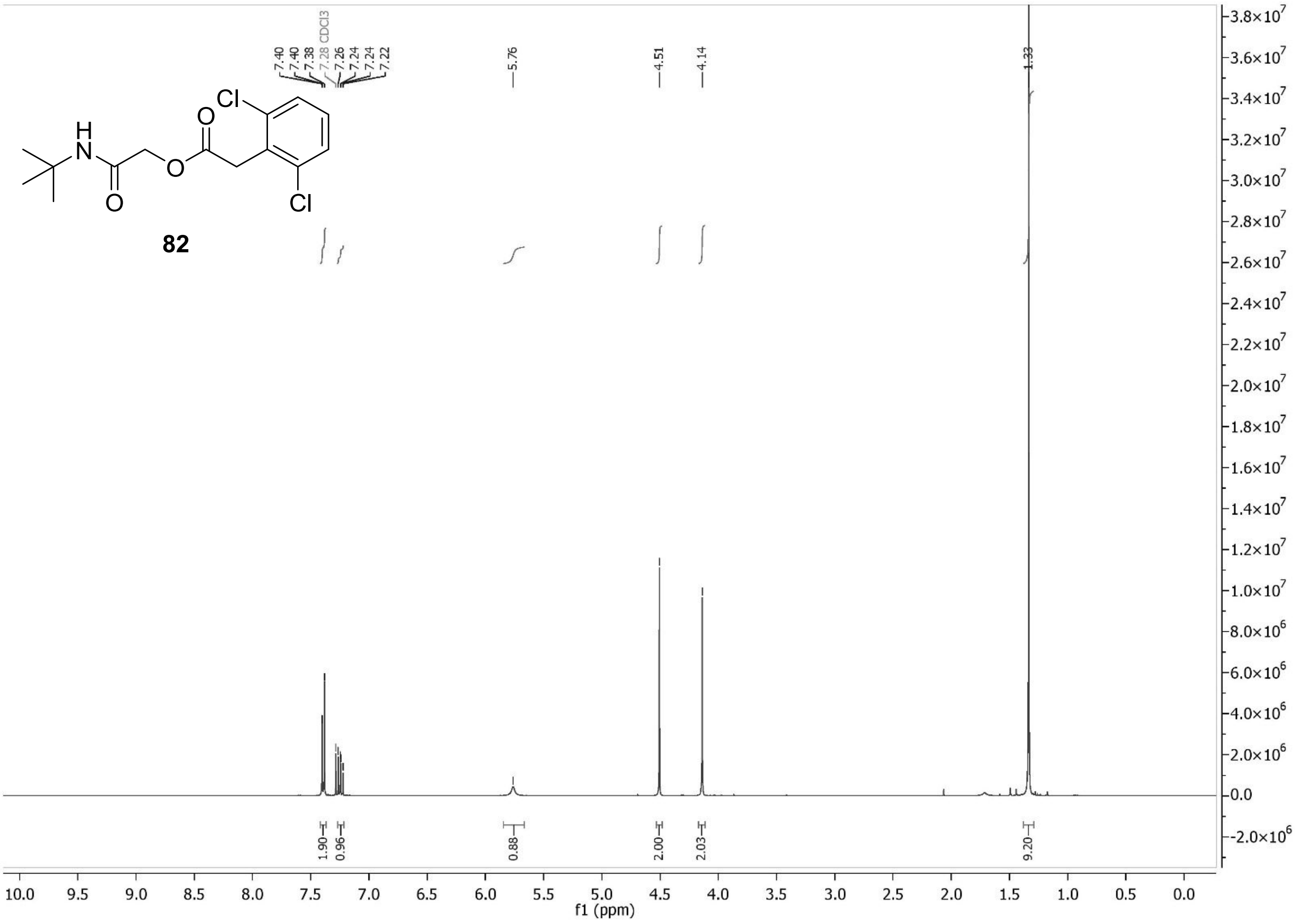

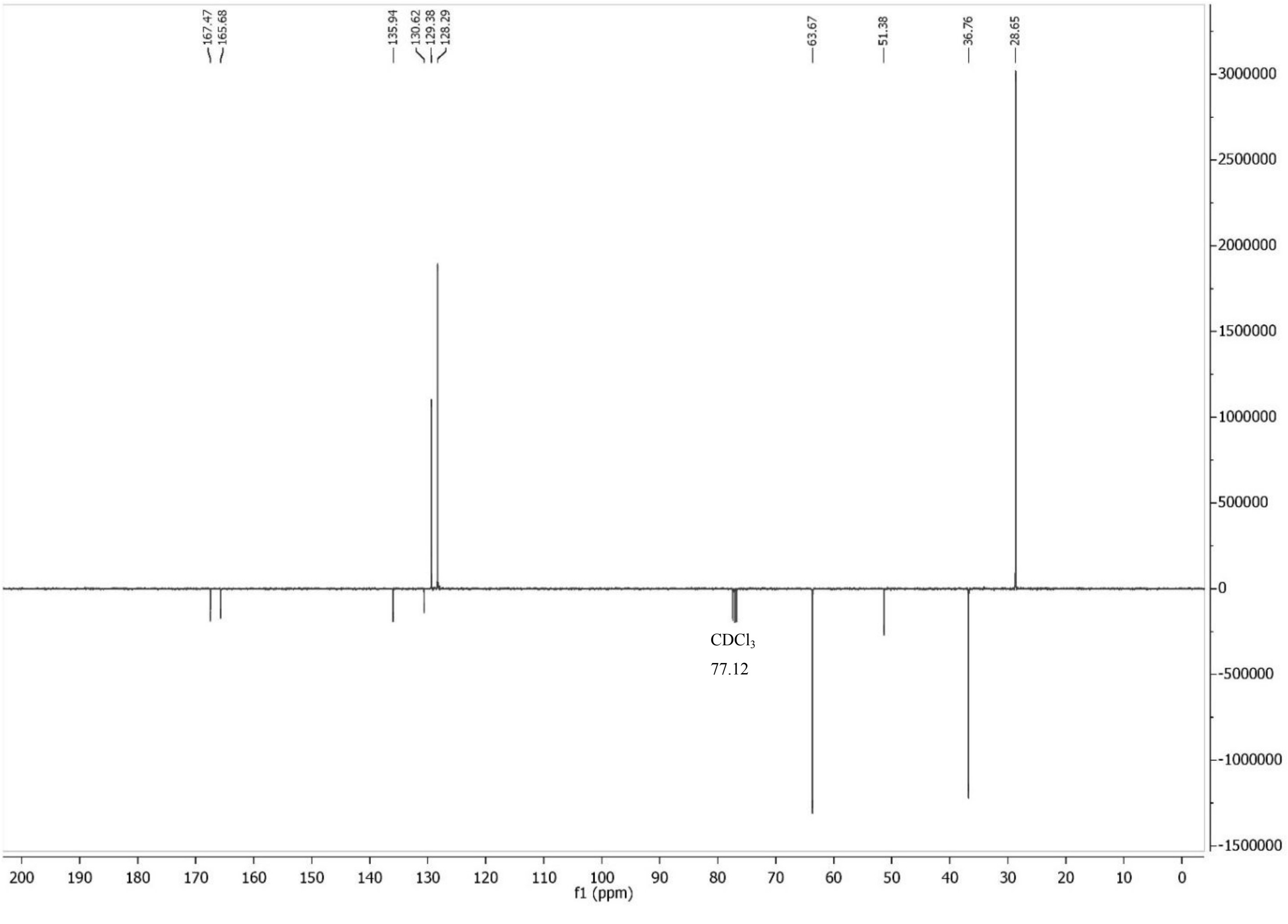

^{1}H and ^{13}C NMR spectra of compound **82**

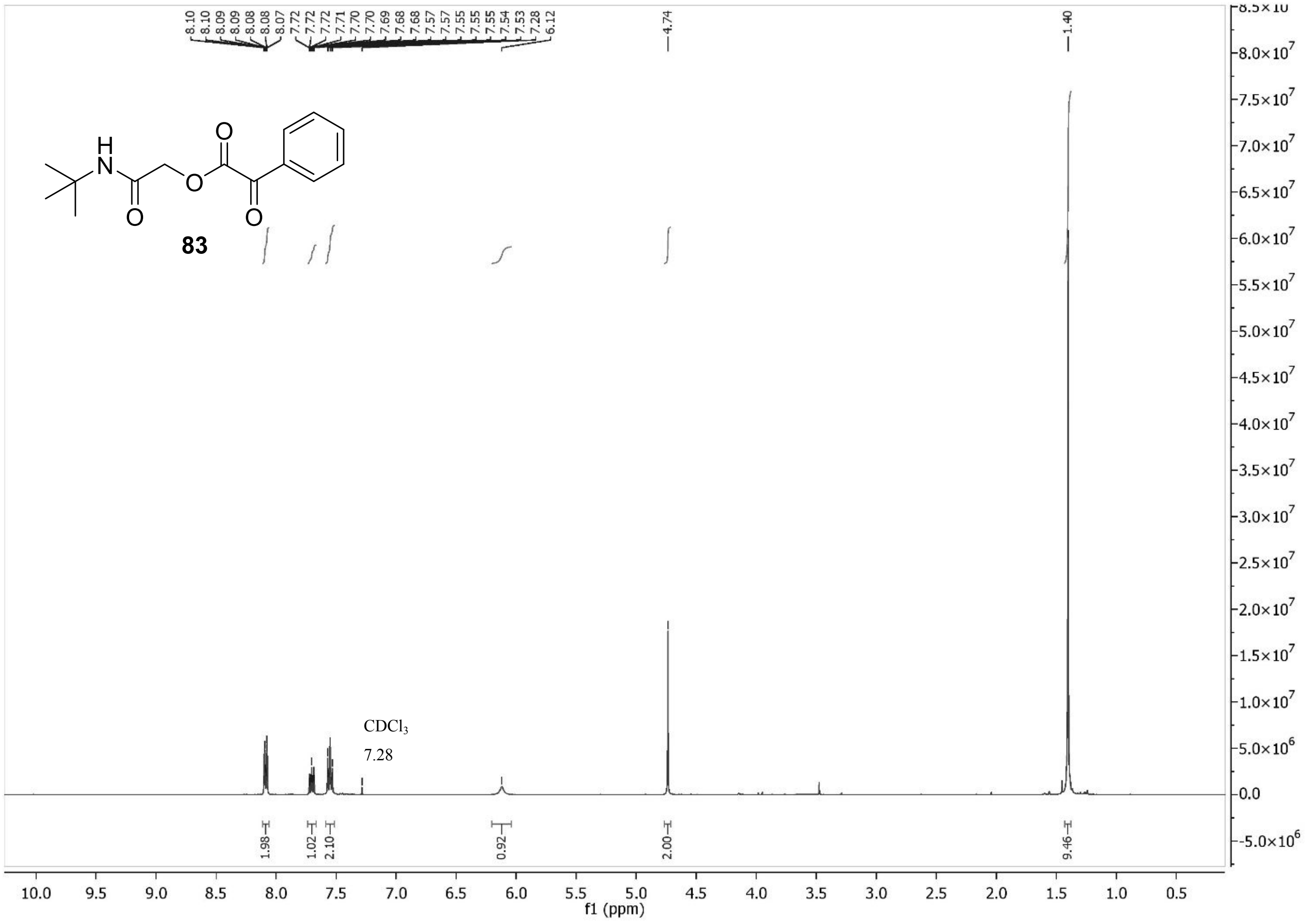

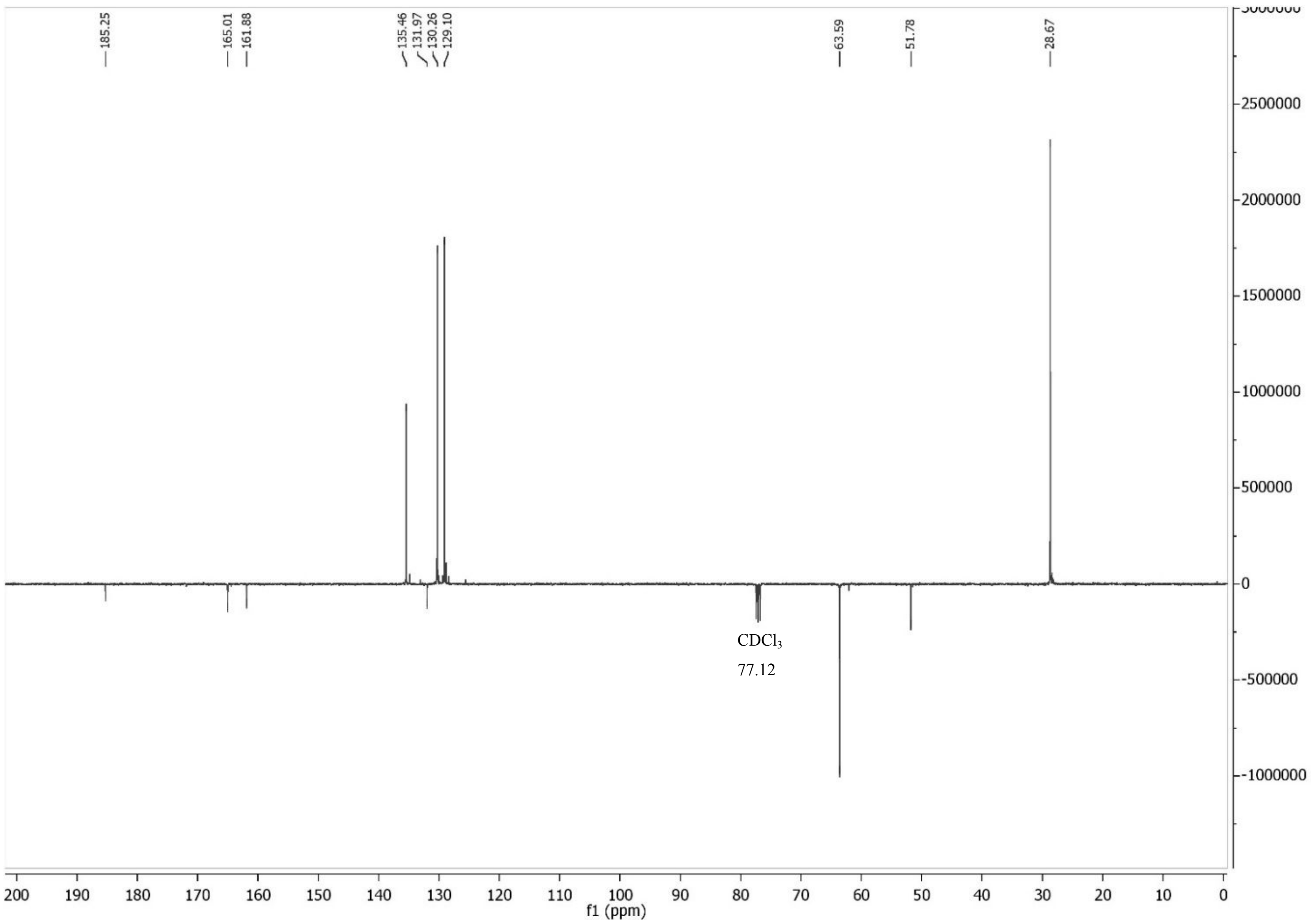

^{1}H and ^{13}C NMR spectra of compound **83**

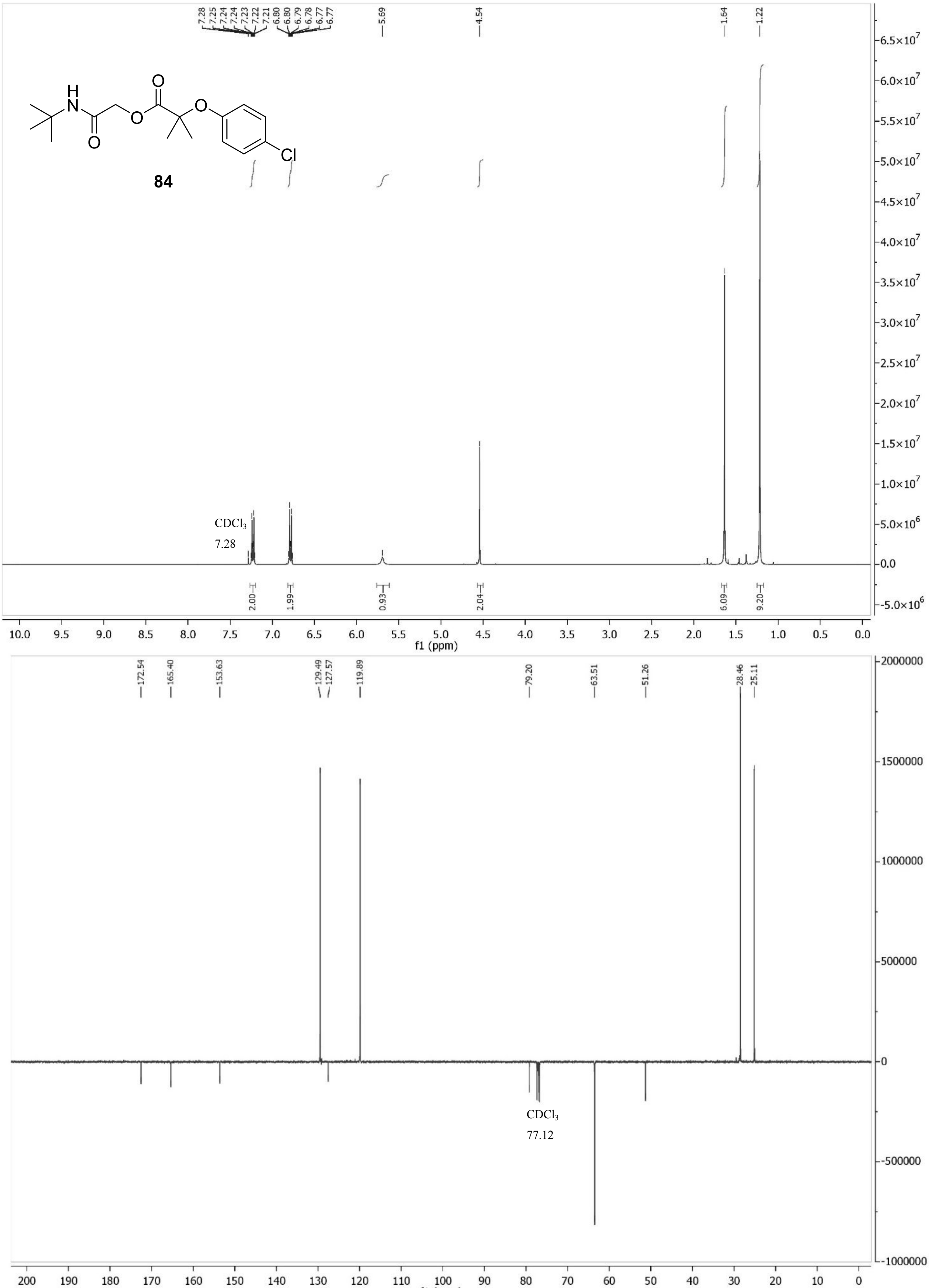

^{1}H and ^{13}C NMR spectra of compound **84**

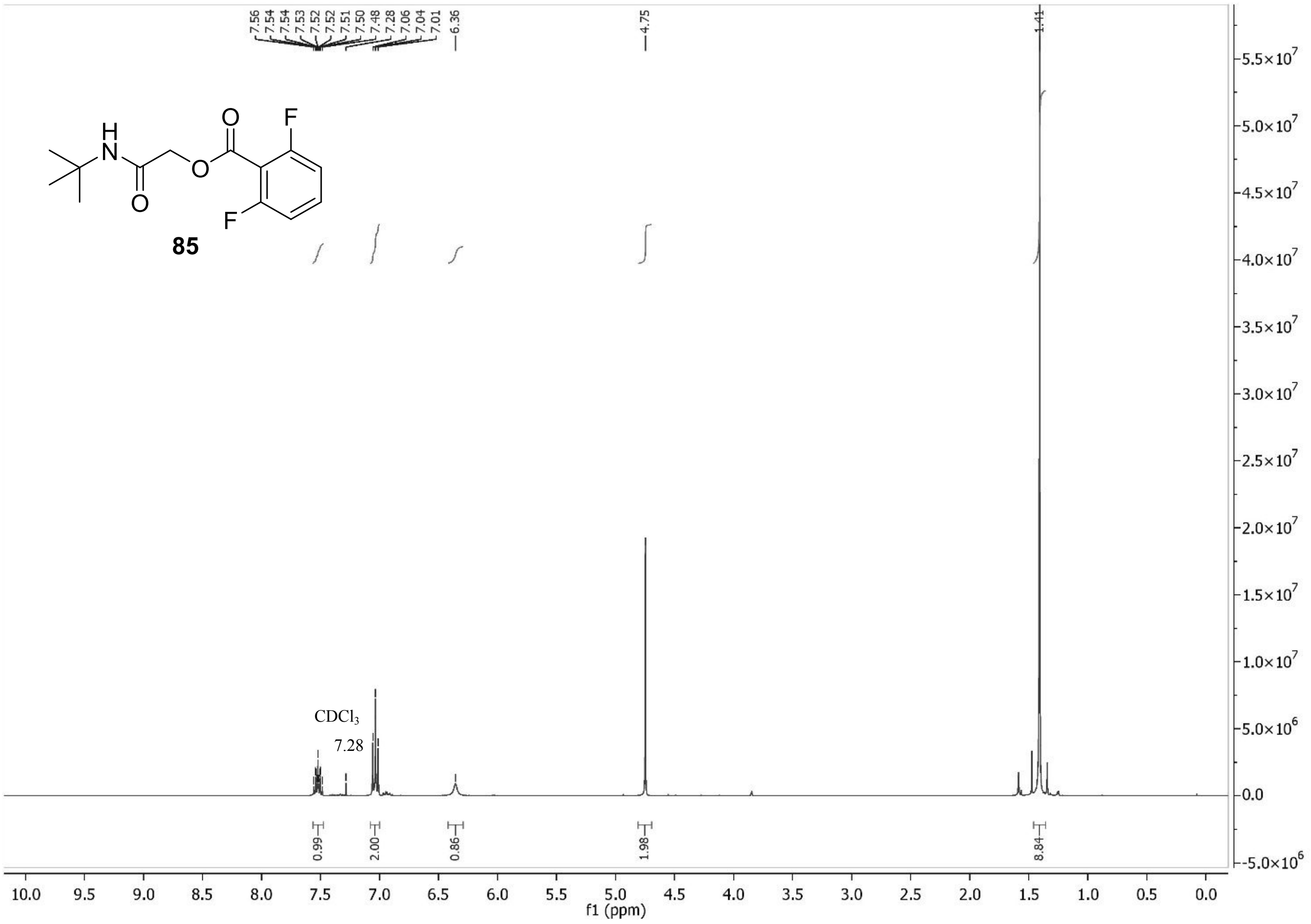

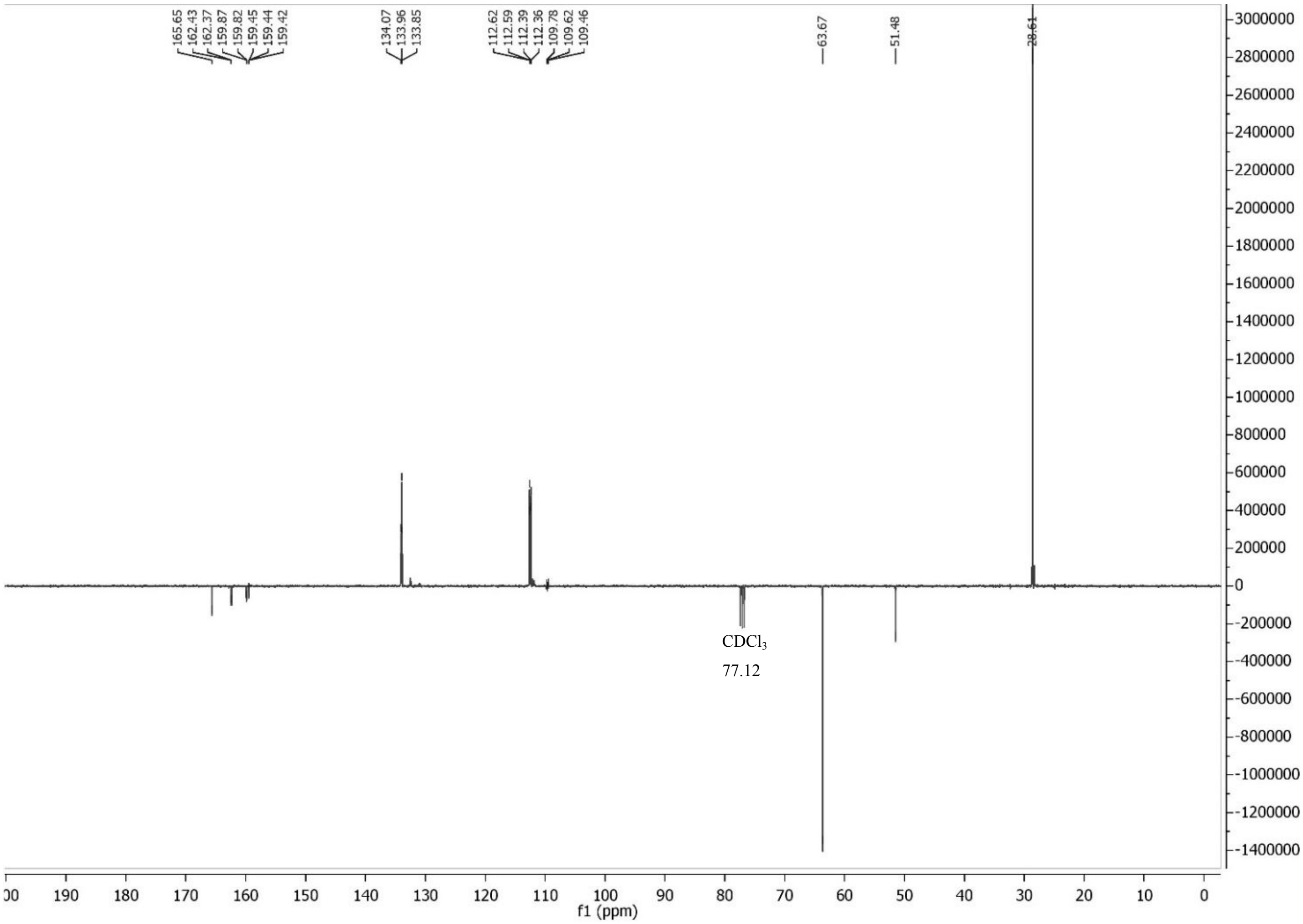

^{1}H and ^{13}C NMR spectra of compound **85**

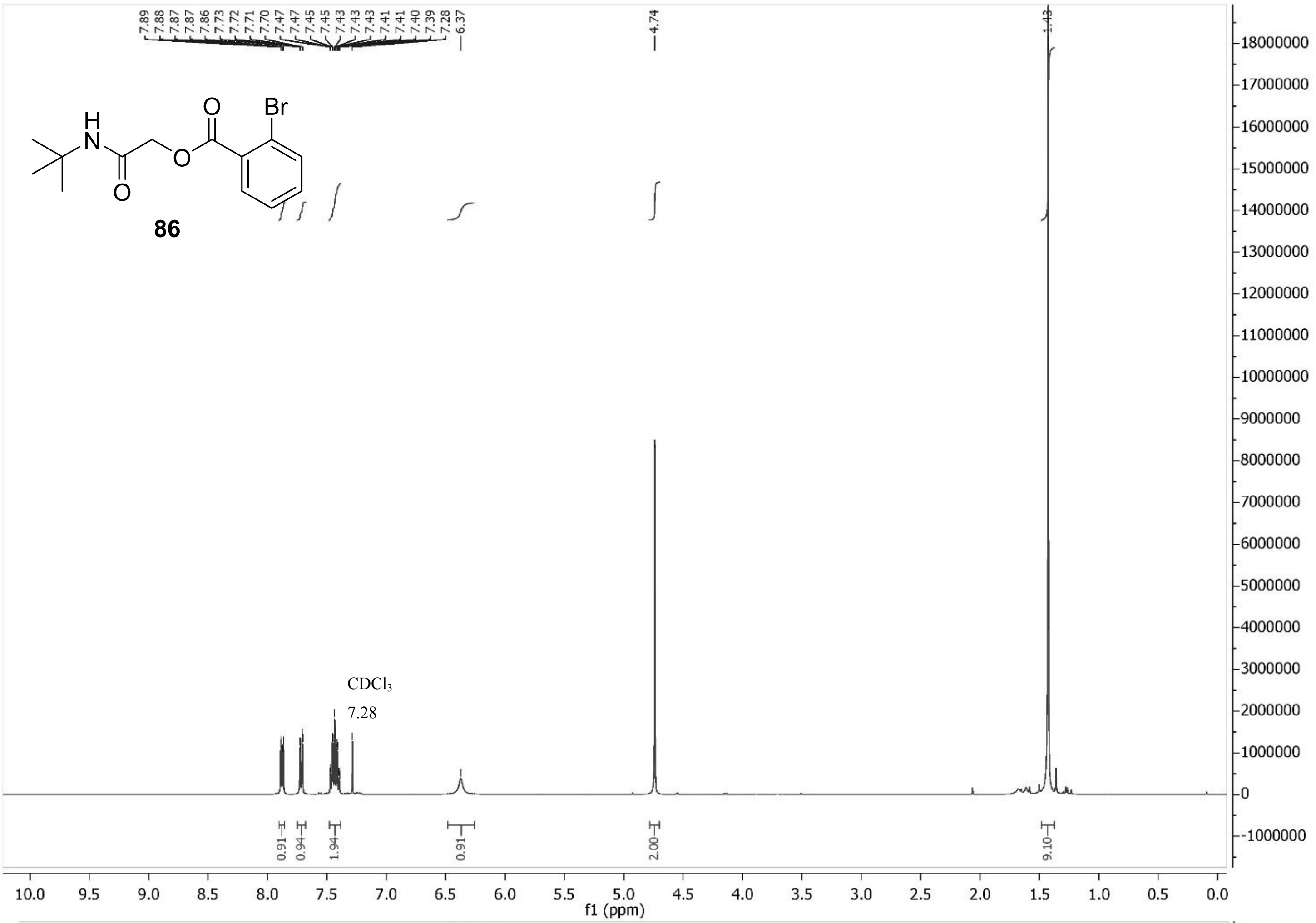

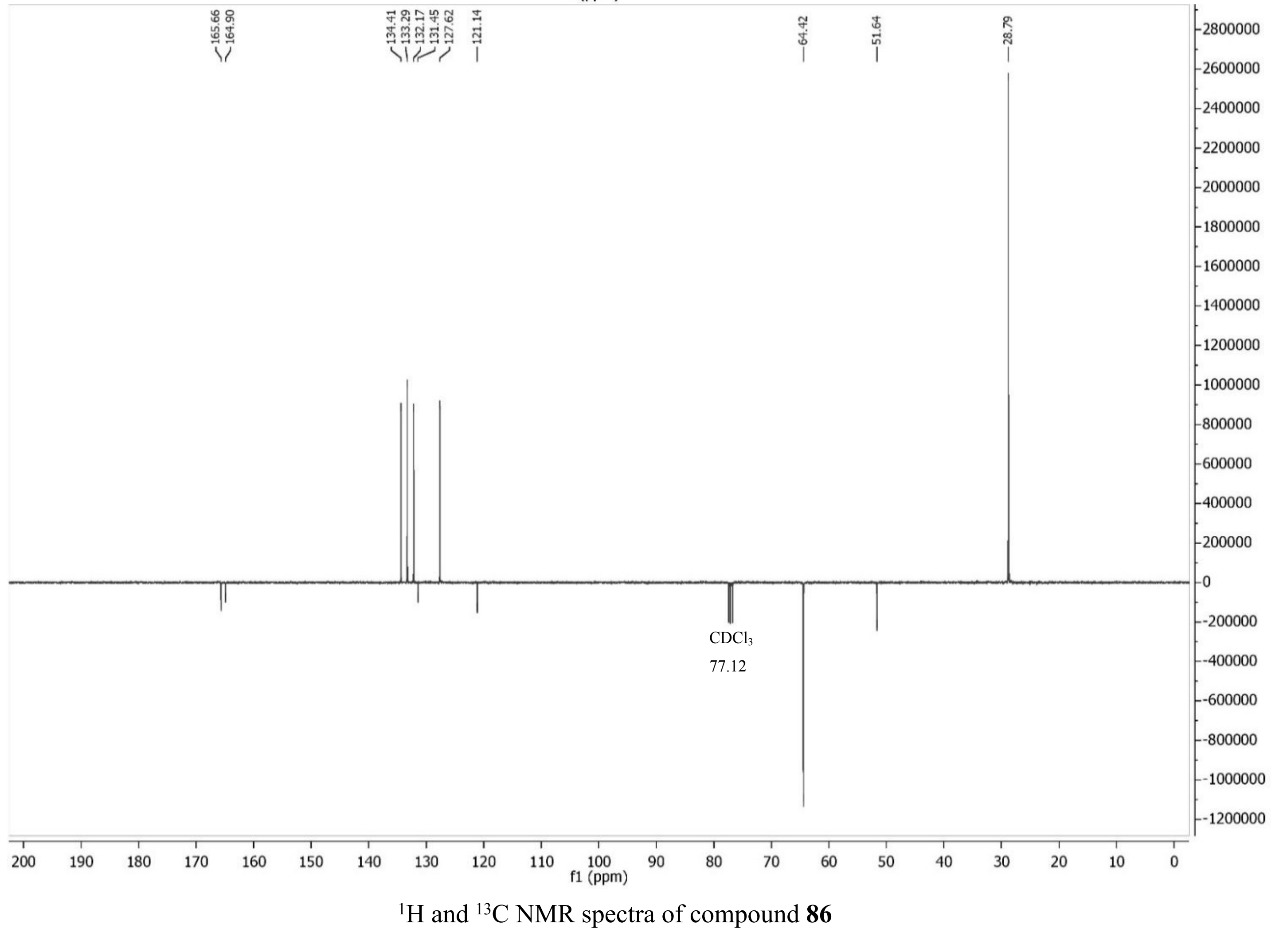

^{1}H and ^{13}C NMR spectra of compound **86**

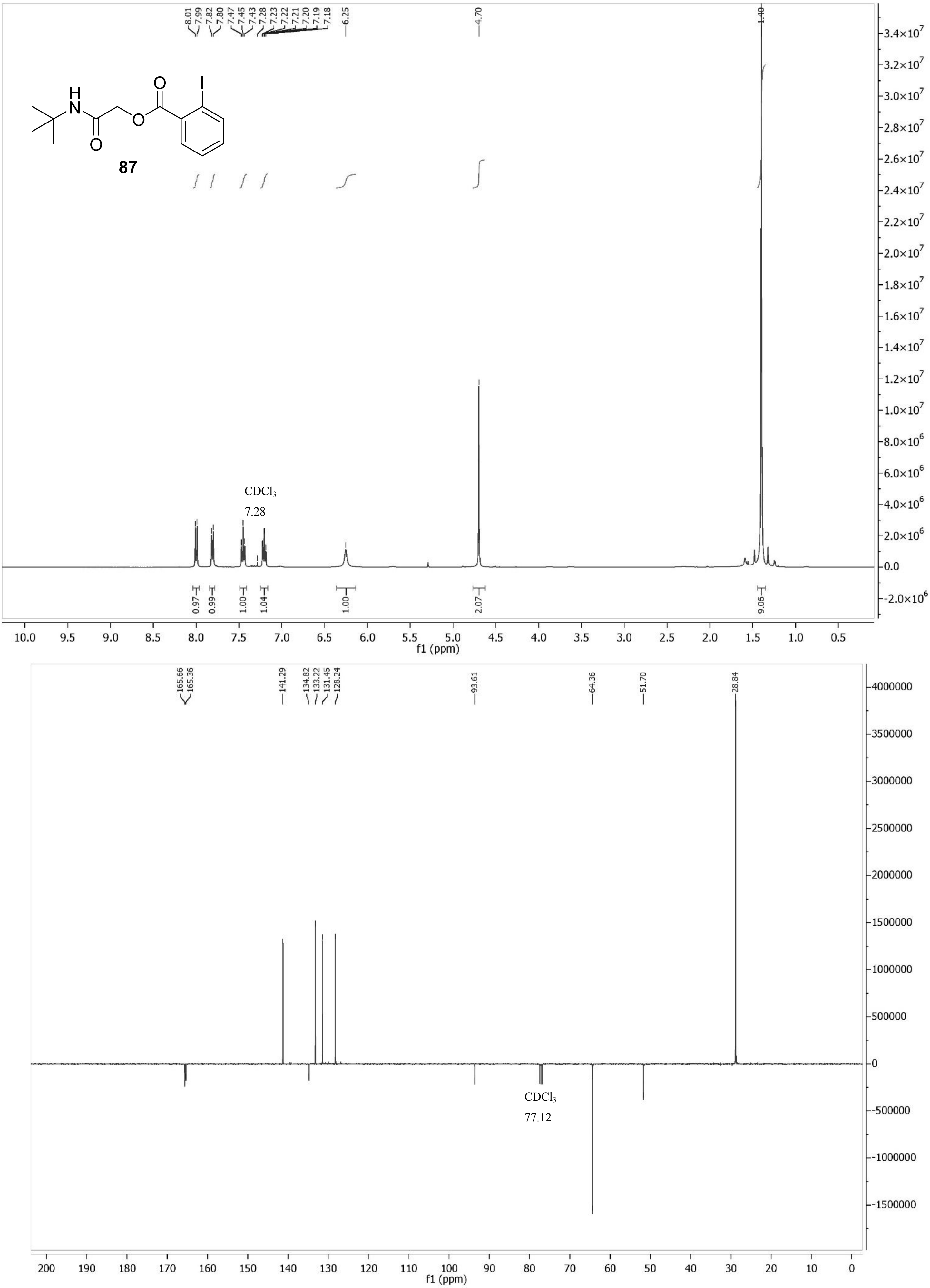

^{1}H and ^{13}C NMR spectra of compound **87**

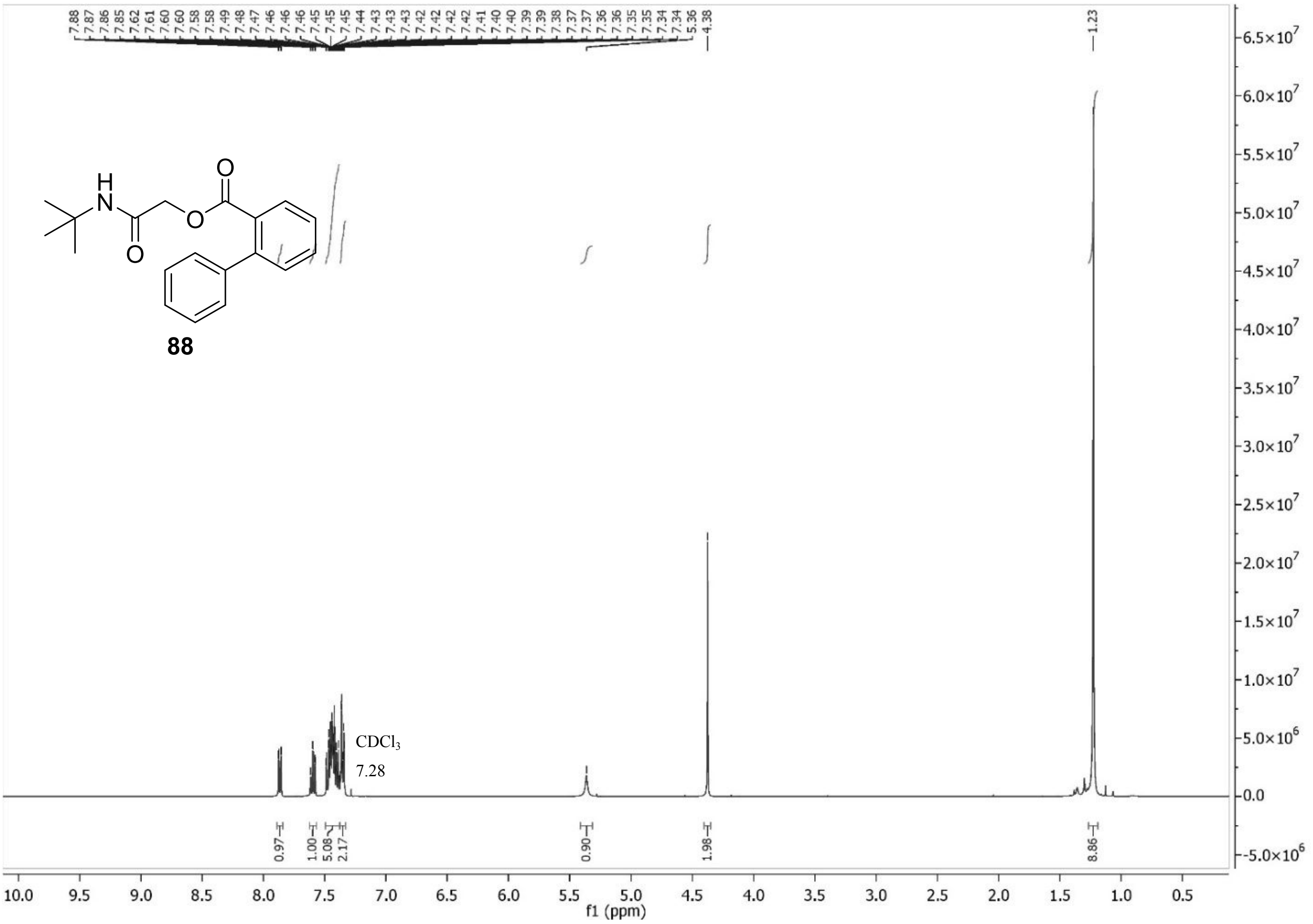

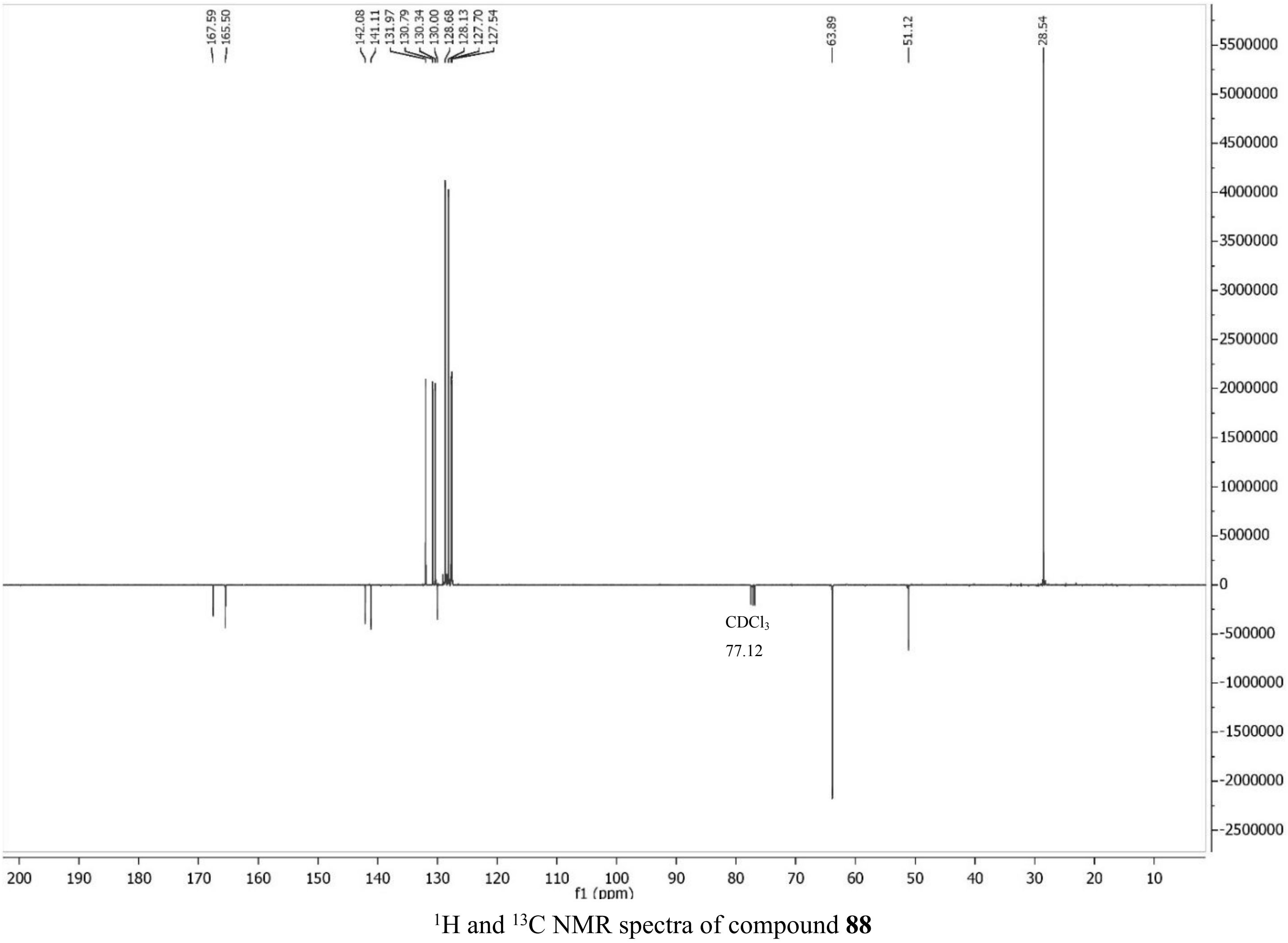

^{1}H and ^{13}C NMR spectra of compound **88**

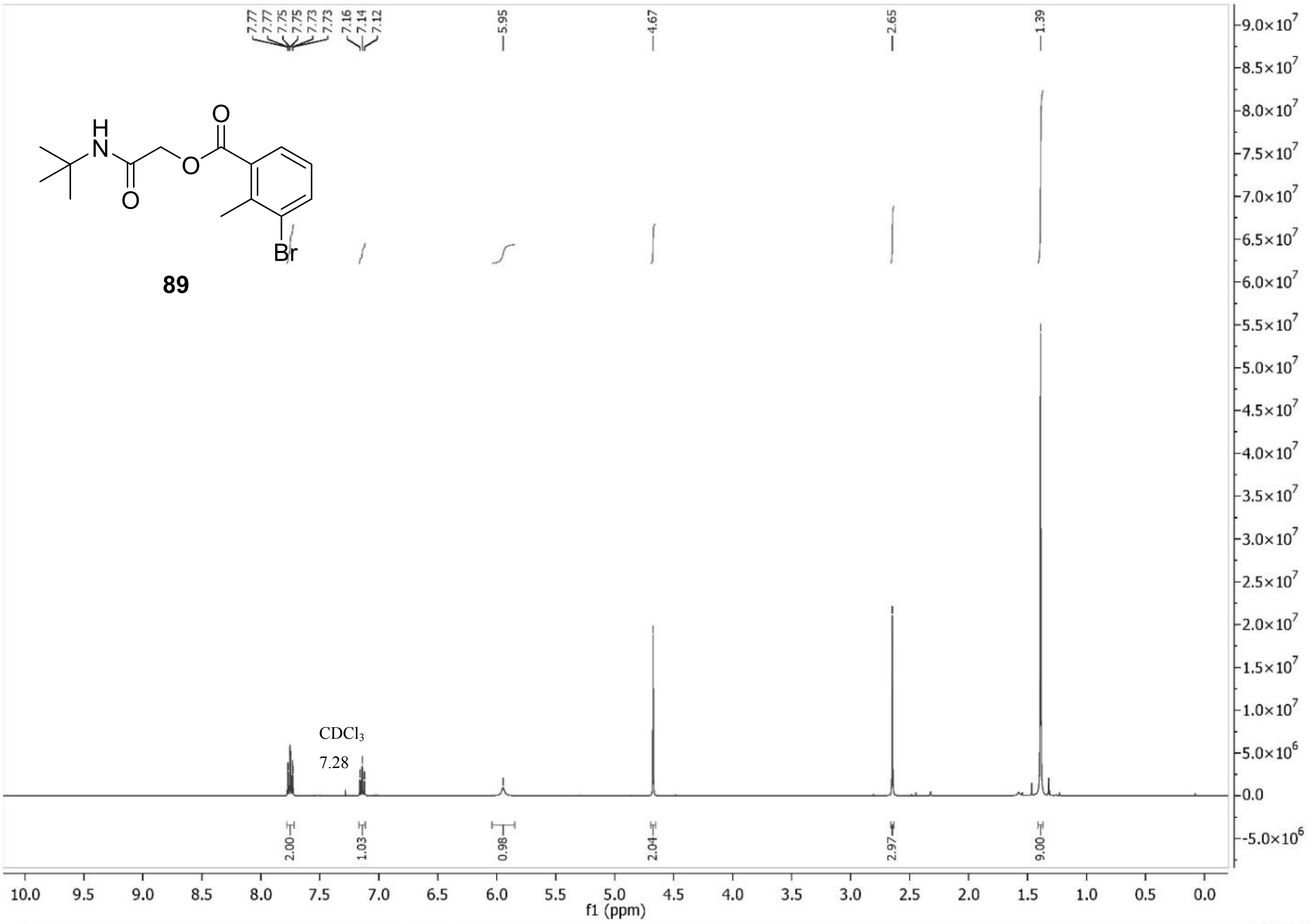

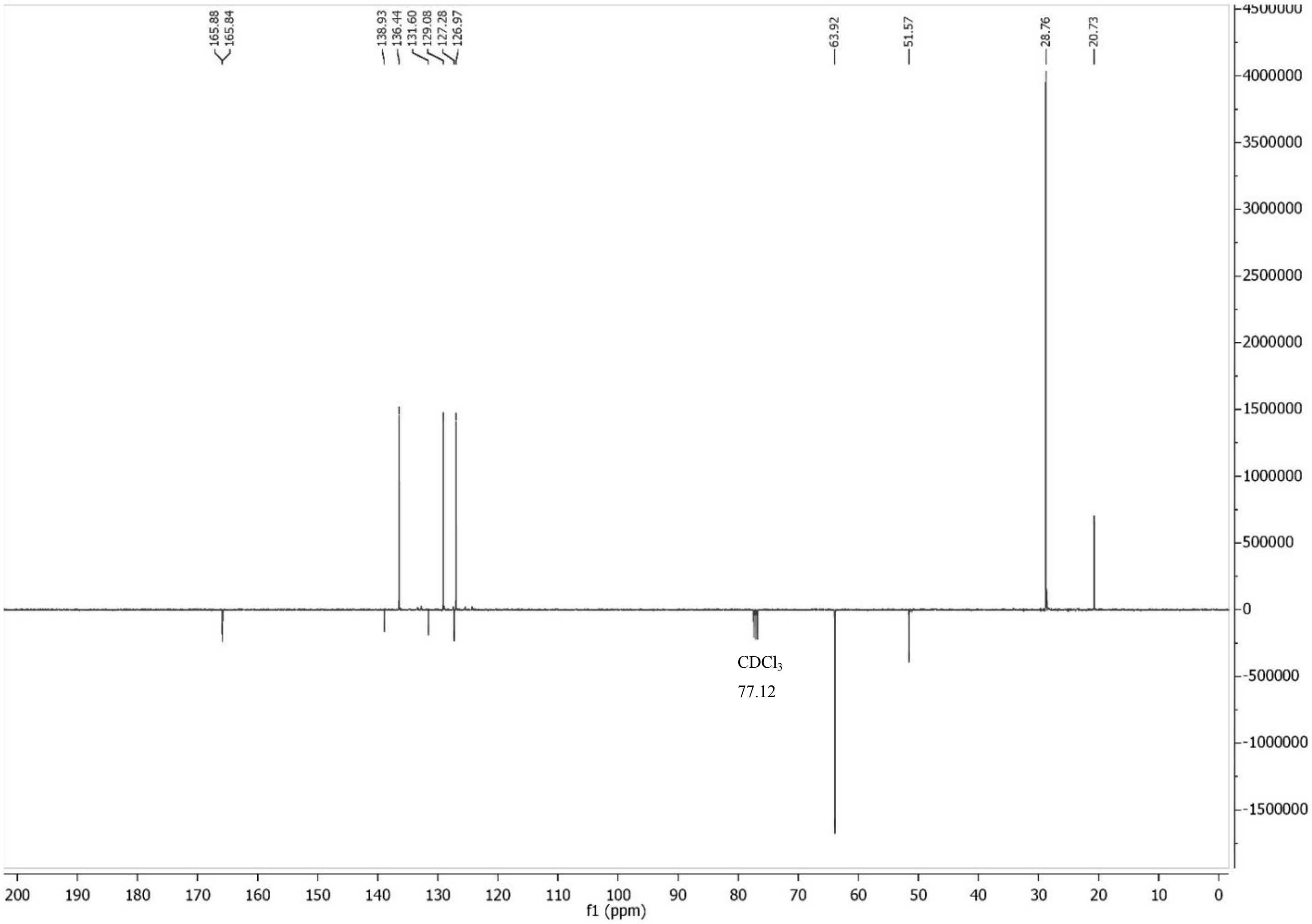

^{1}H and ^{13}C NMR spectra of compound **89**

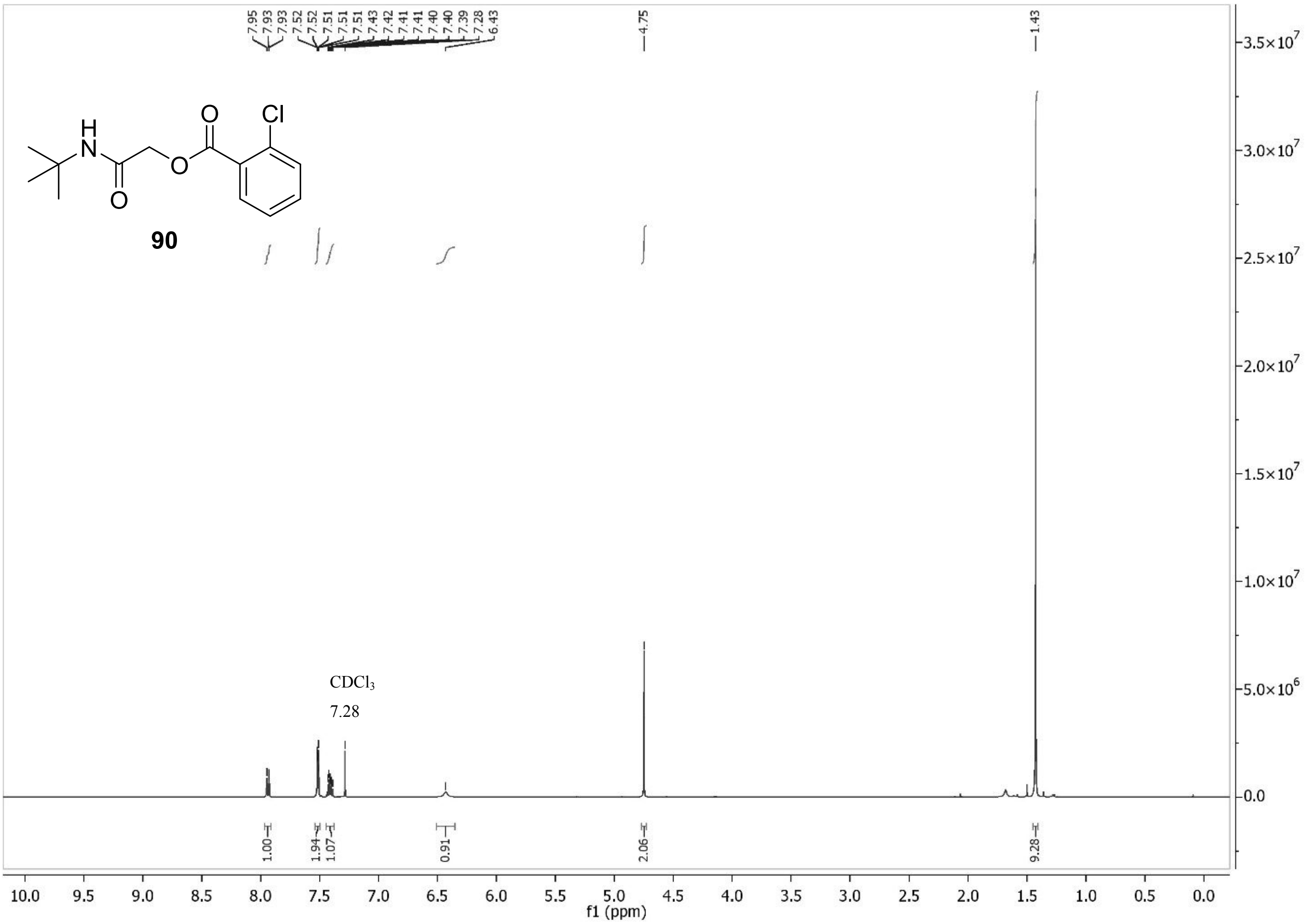

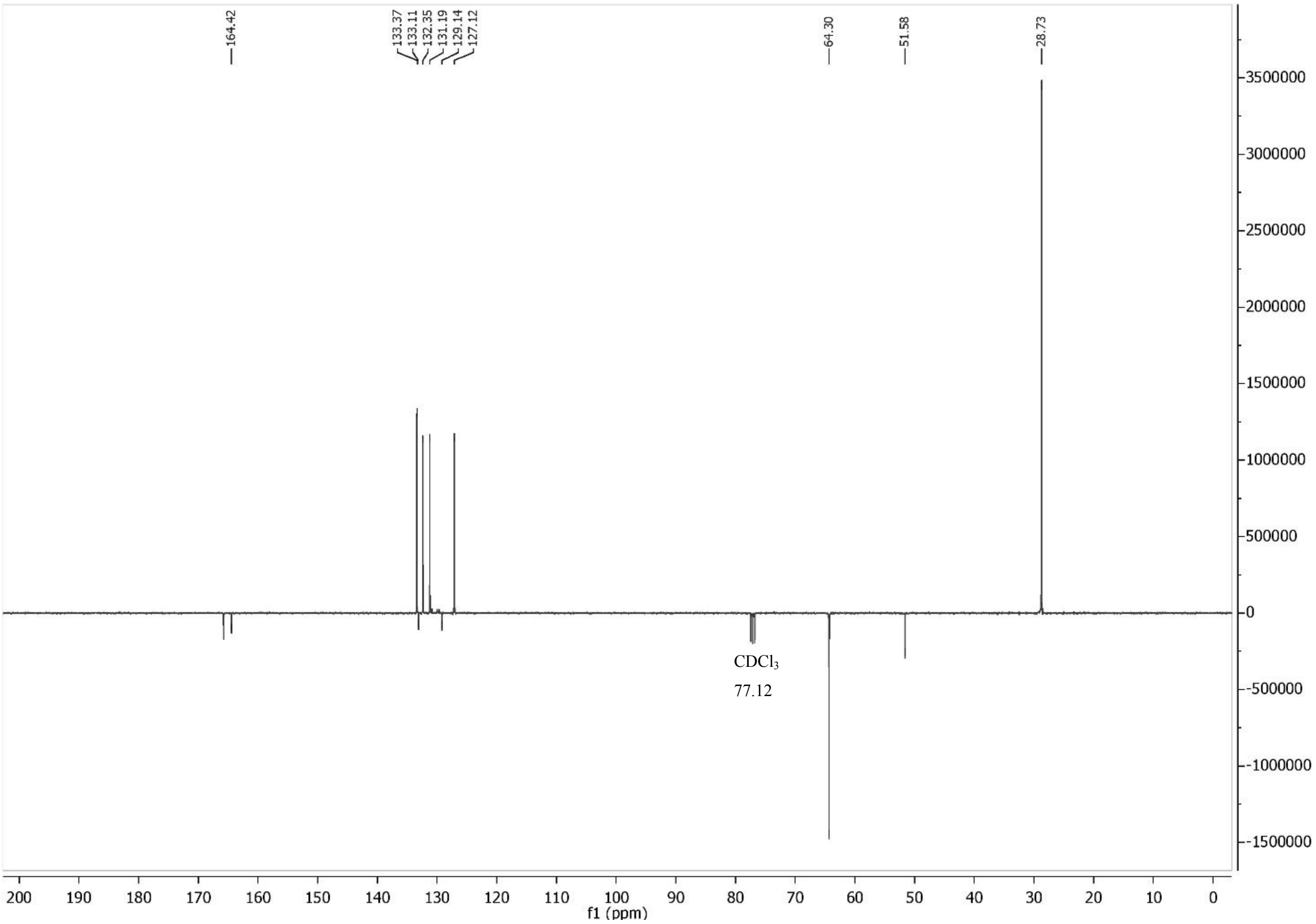

^{1}H and ^{13}C NMR spectra of compound **90**

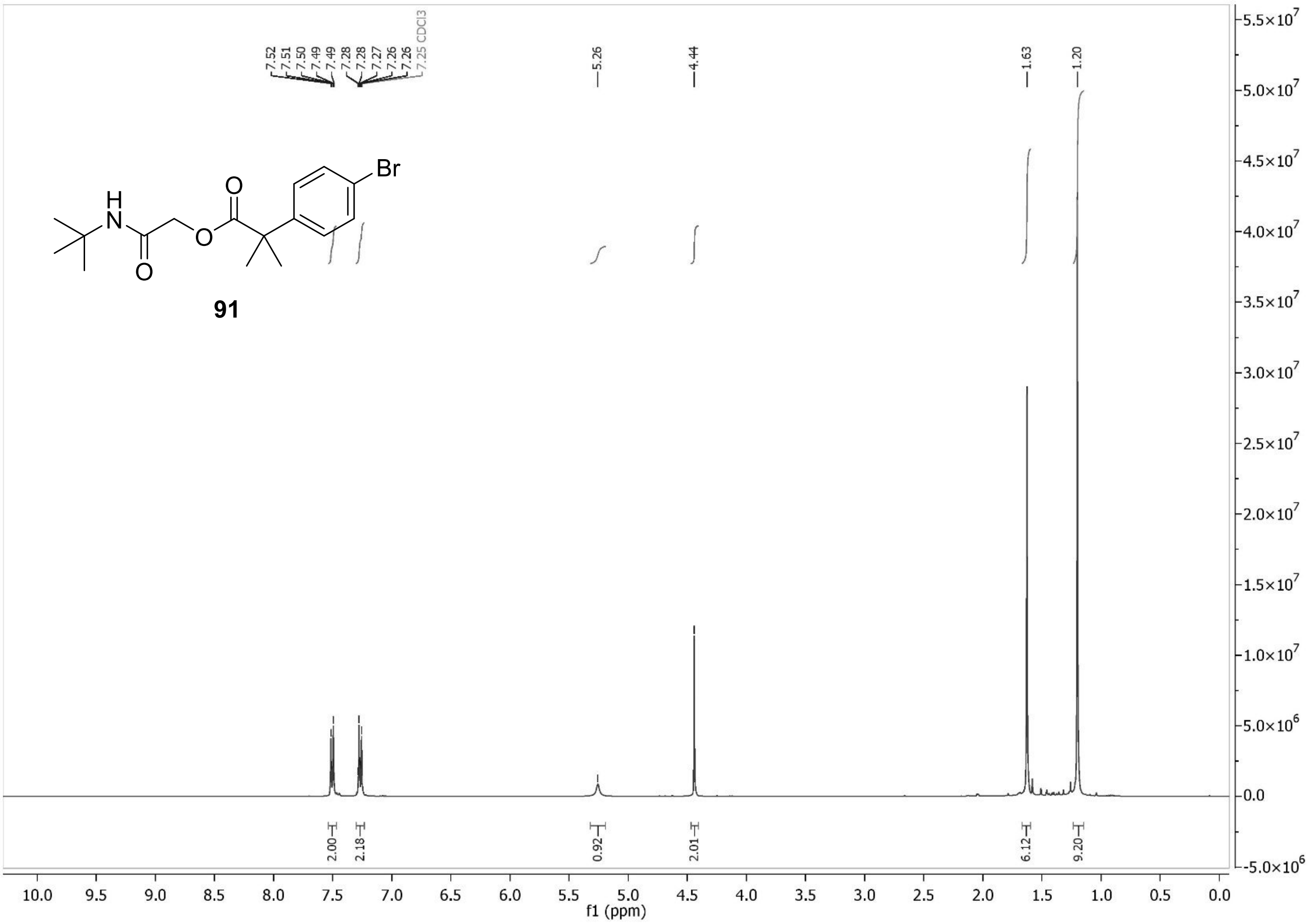

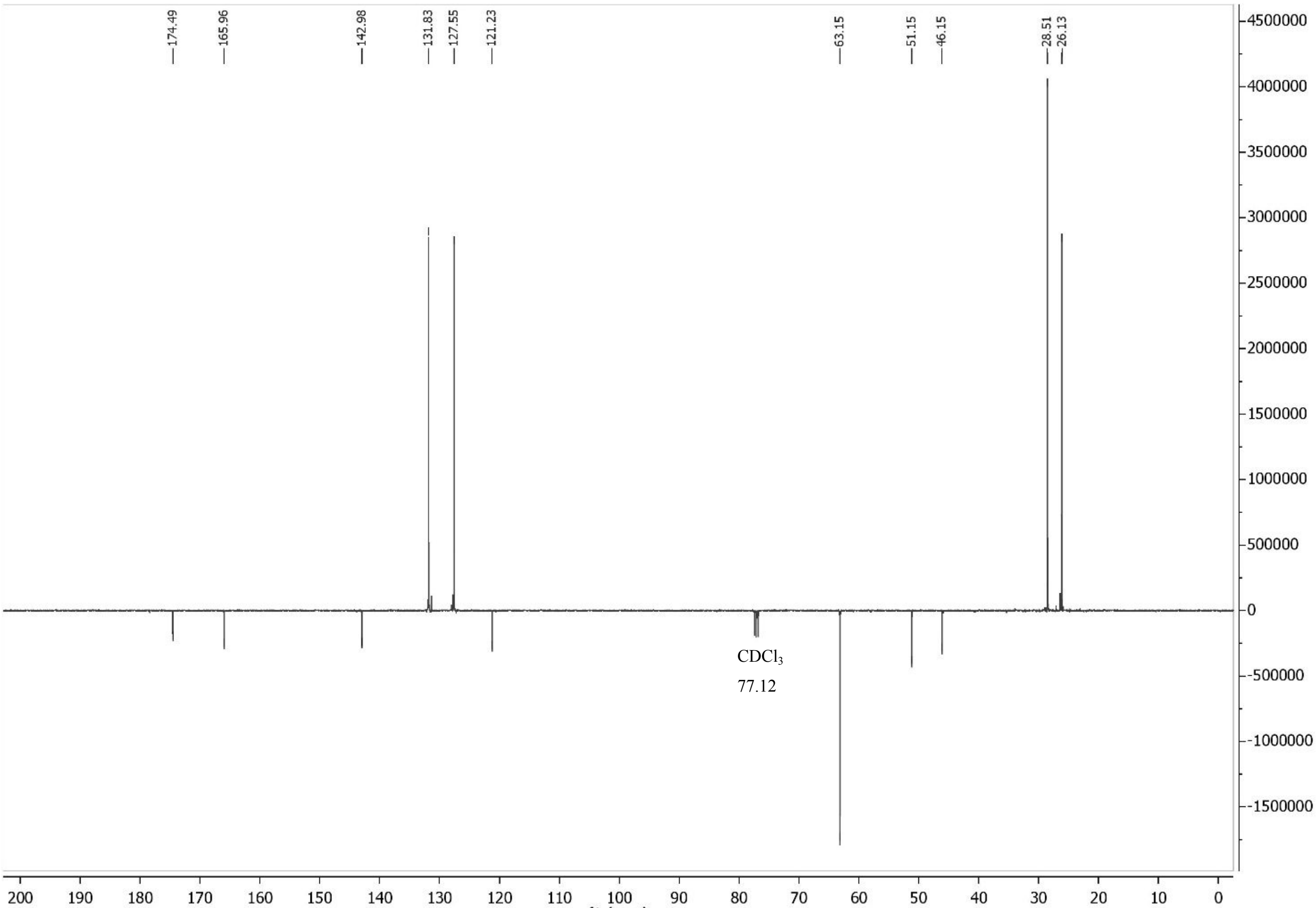

^{1}H and ^{13}C NMR spectra of compound **91**

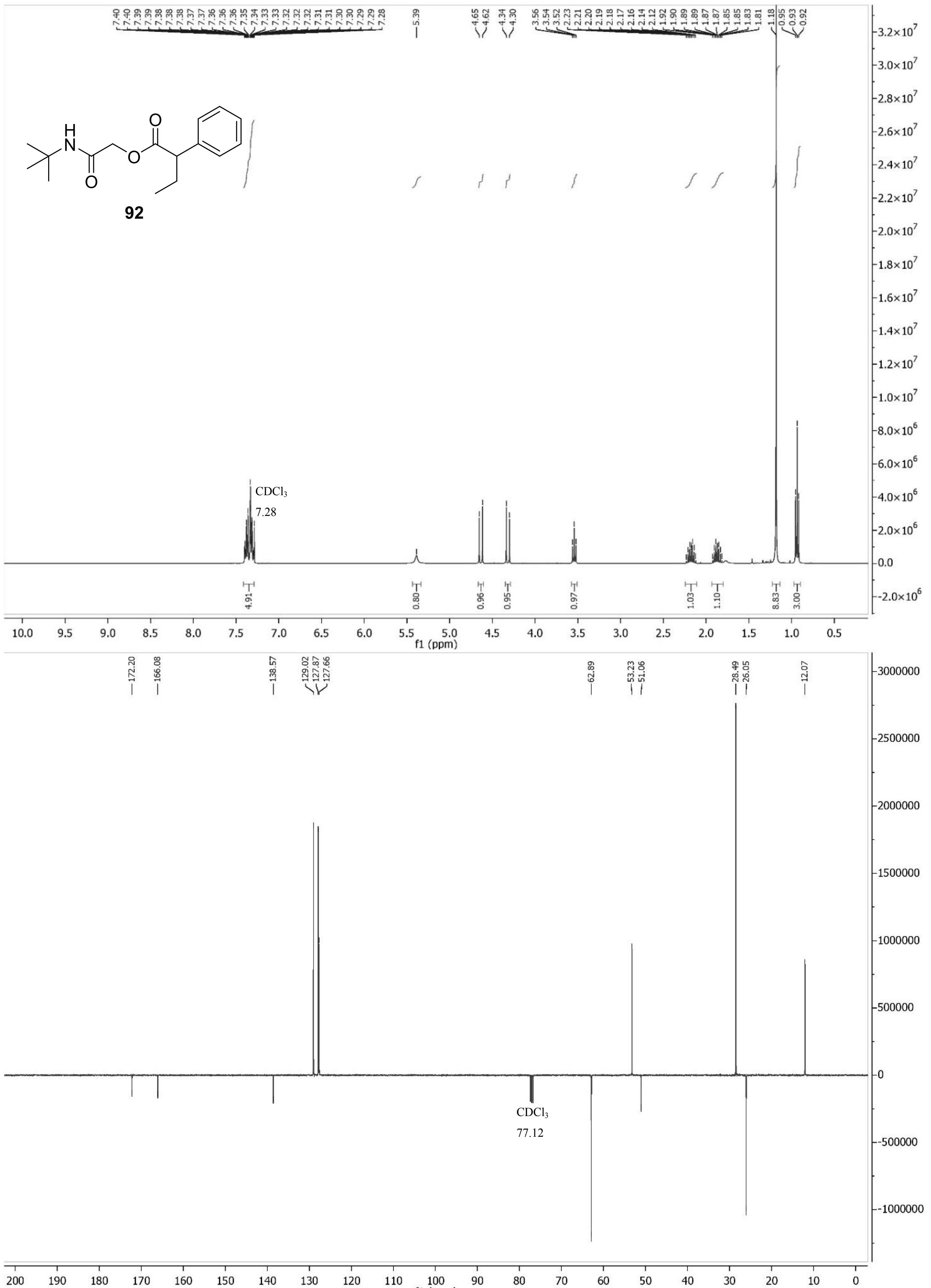

^{1}H and ^{13}C NMR spectra of compound **92**

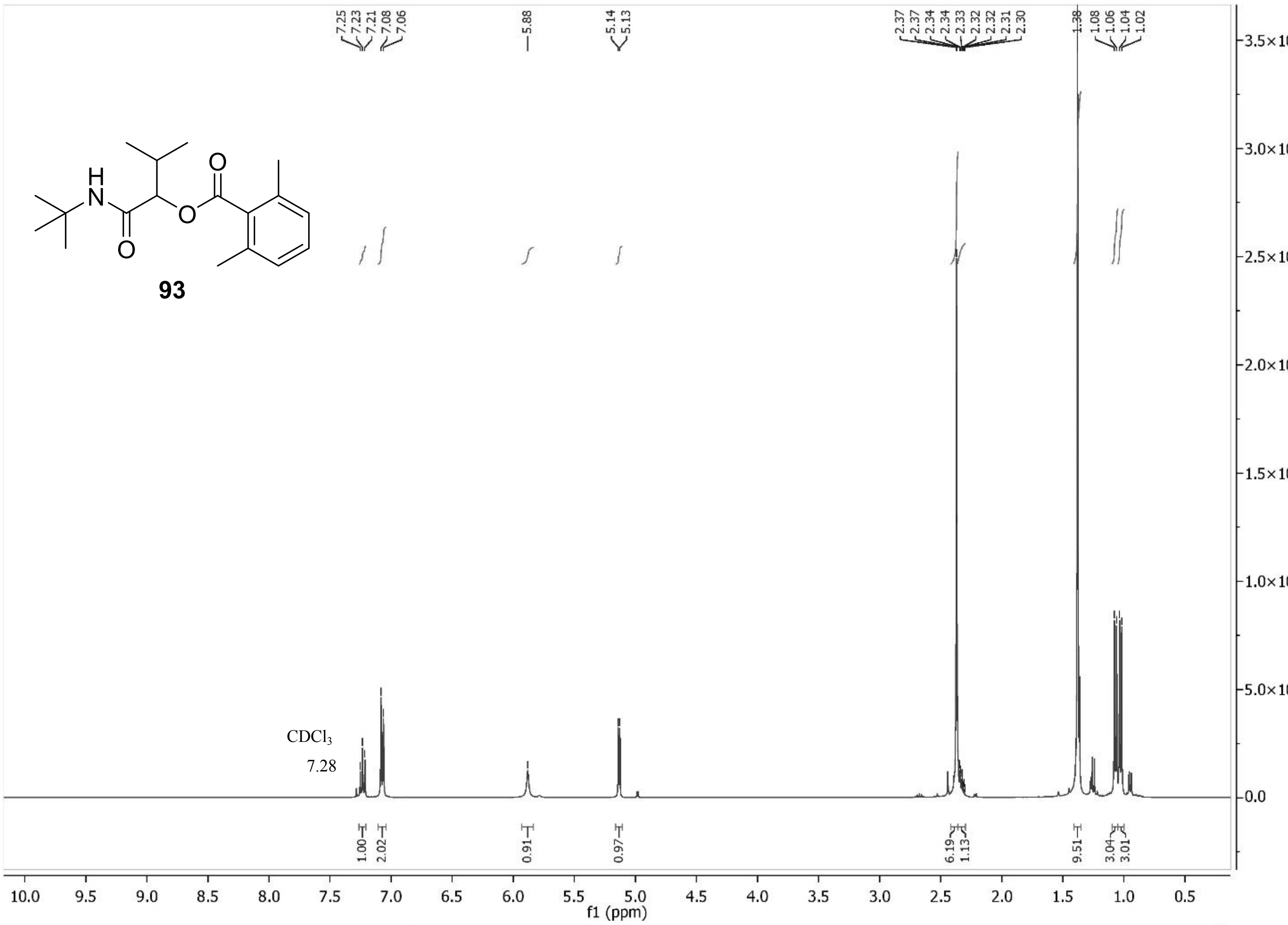

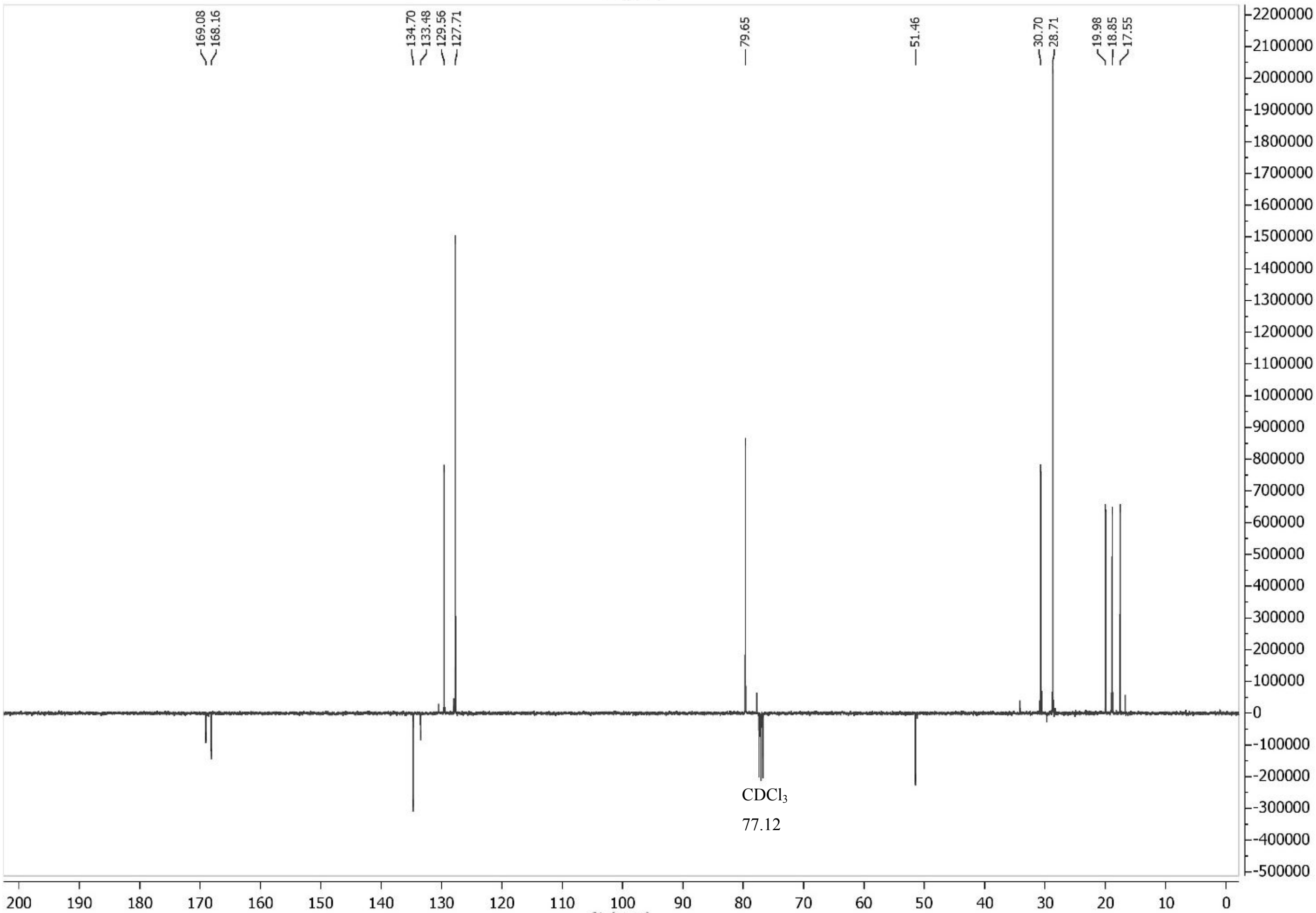

^{1}H and ^{13}C NMR spectra of compound **93**

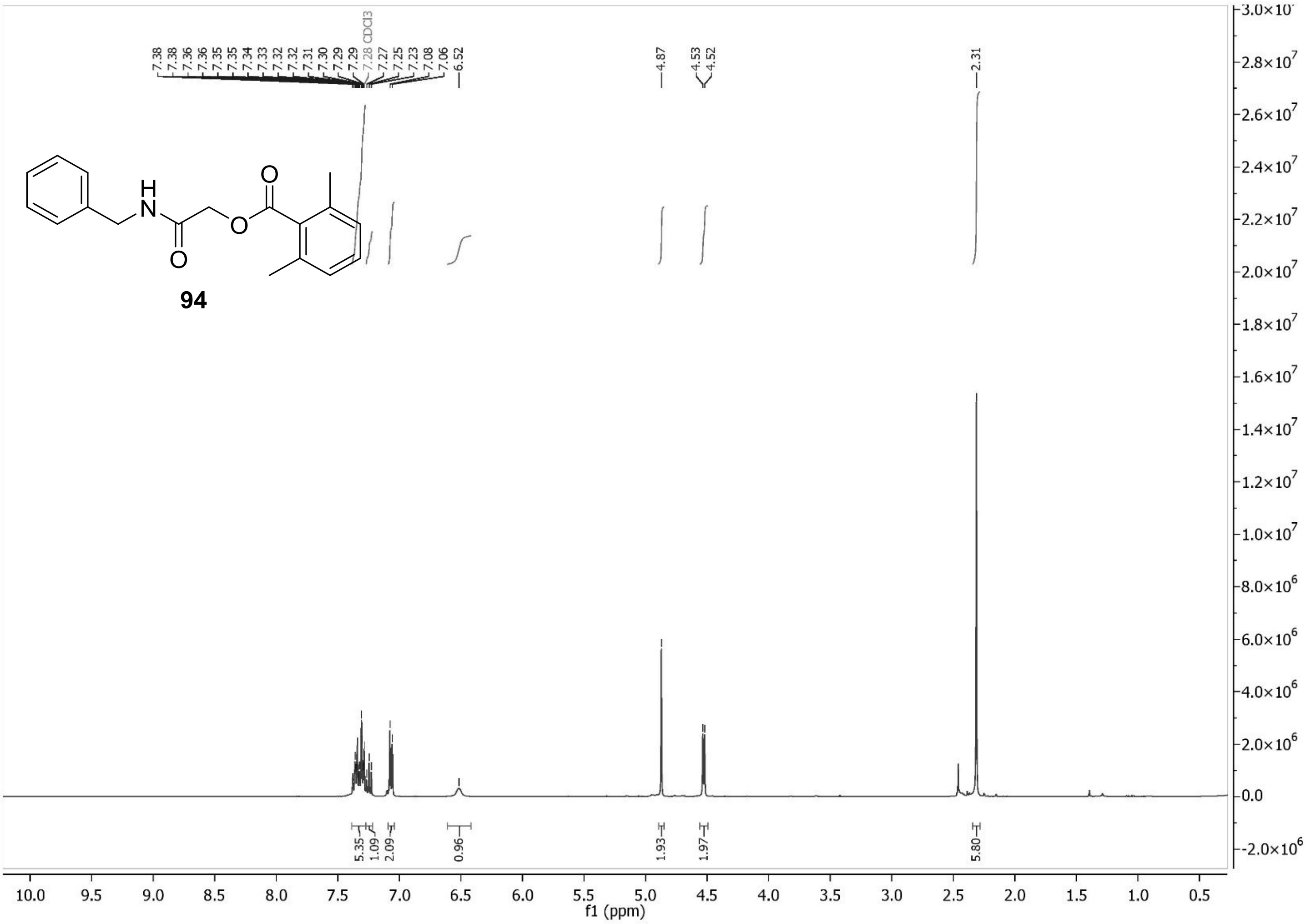

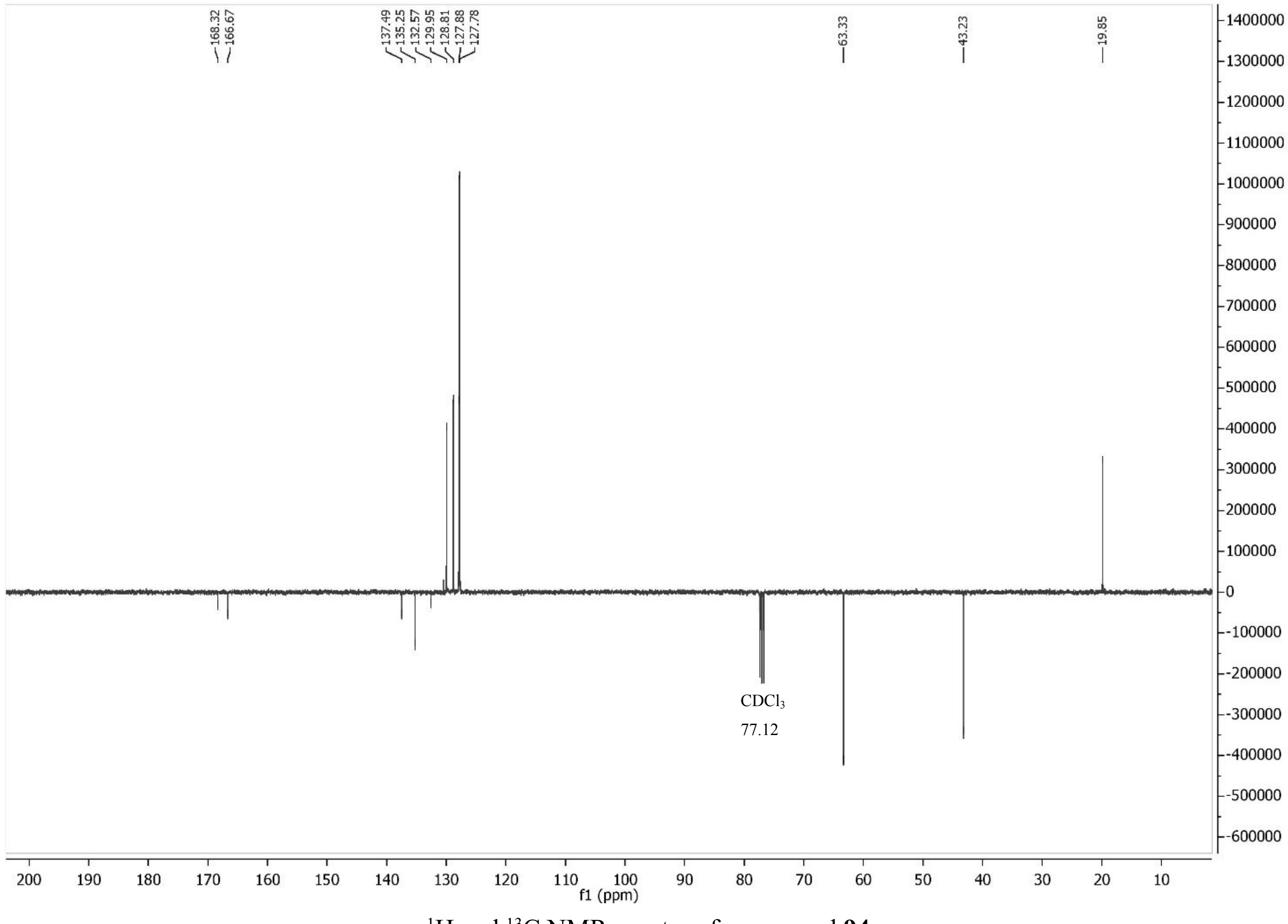

^{1}H and ^{13}C NMR spectra of compound **94**

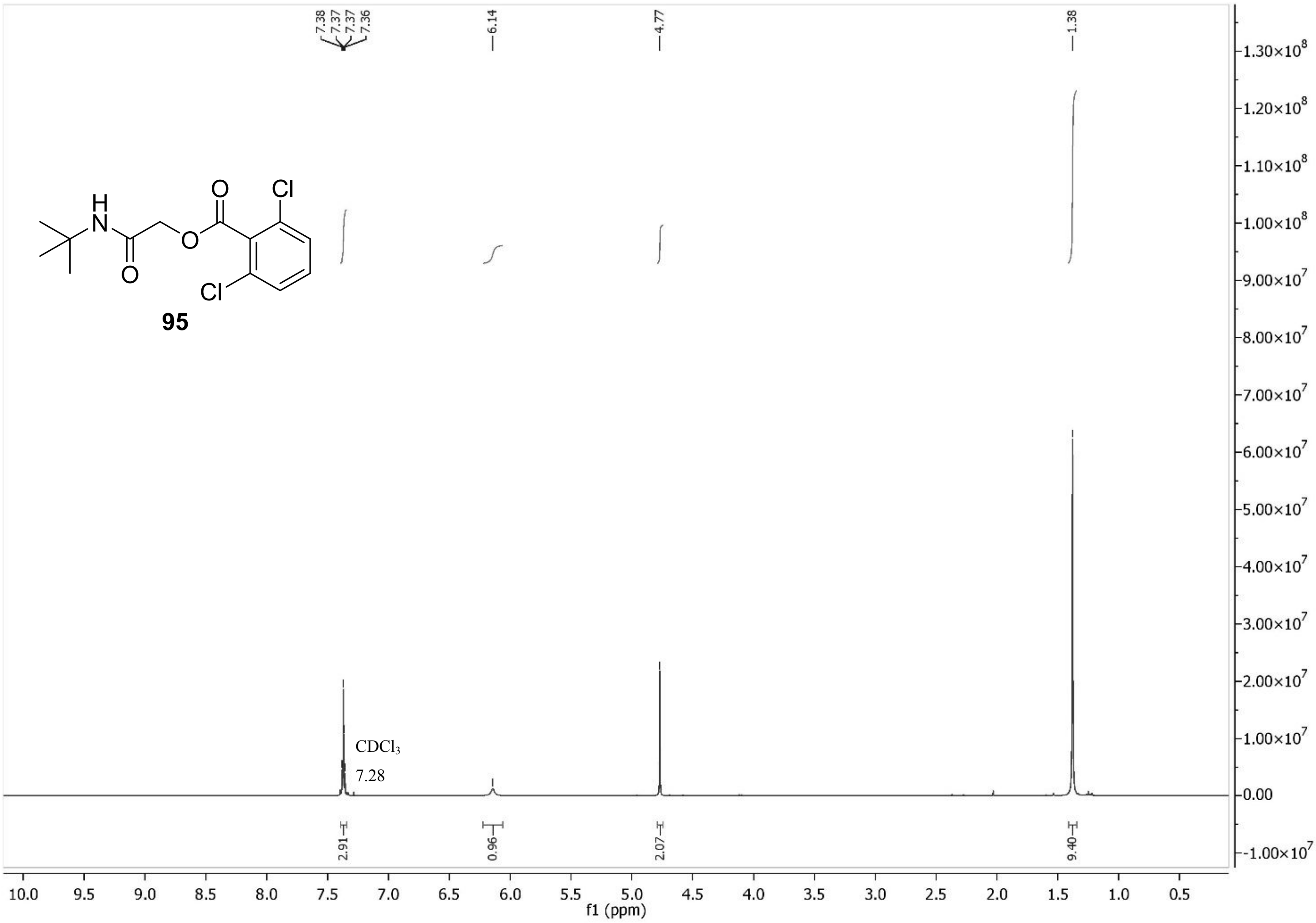

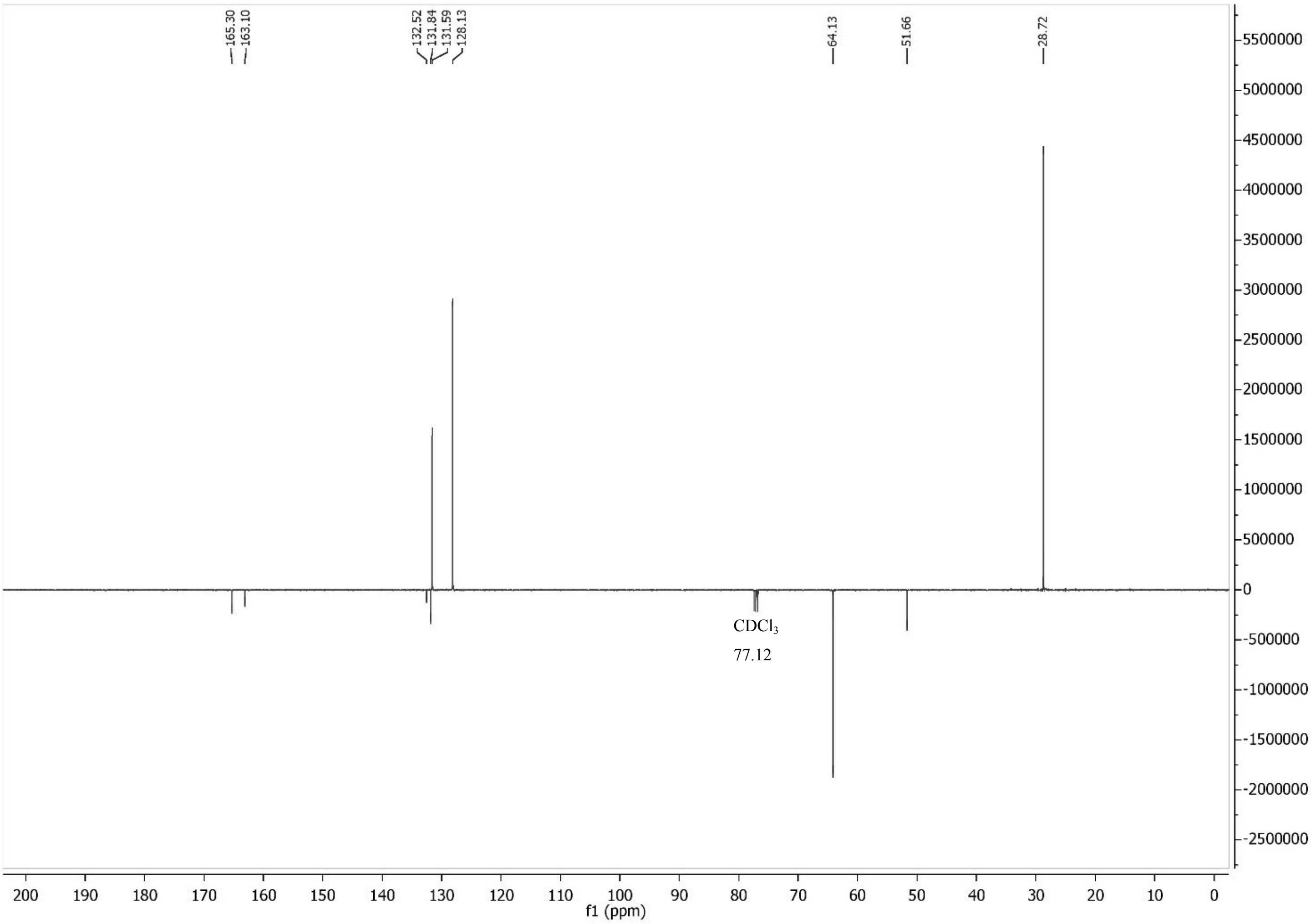

^{1}H and ^{13}C NMR spectra of compound **95**

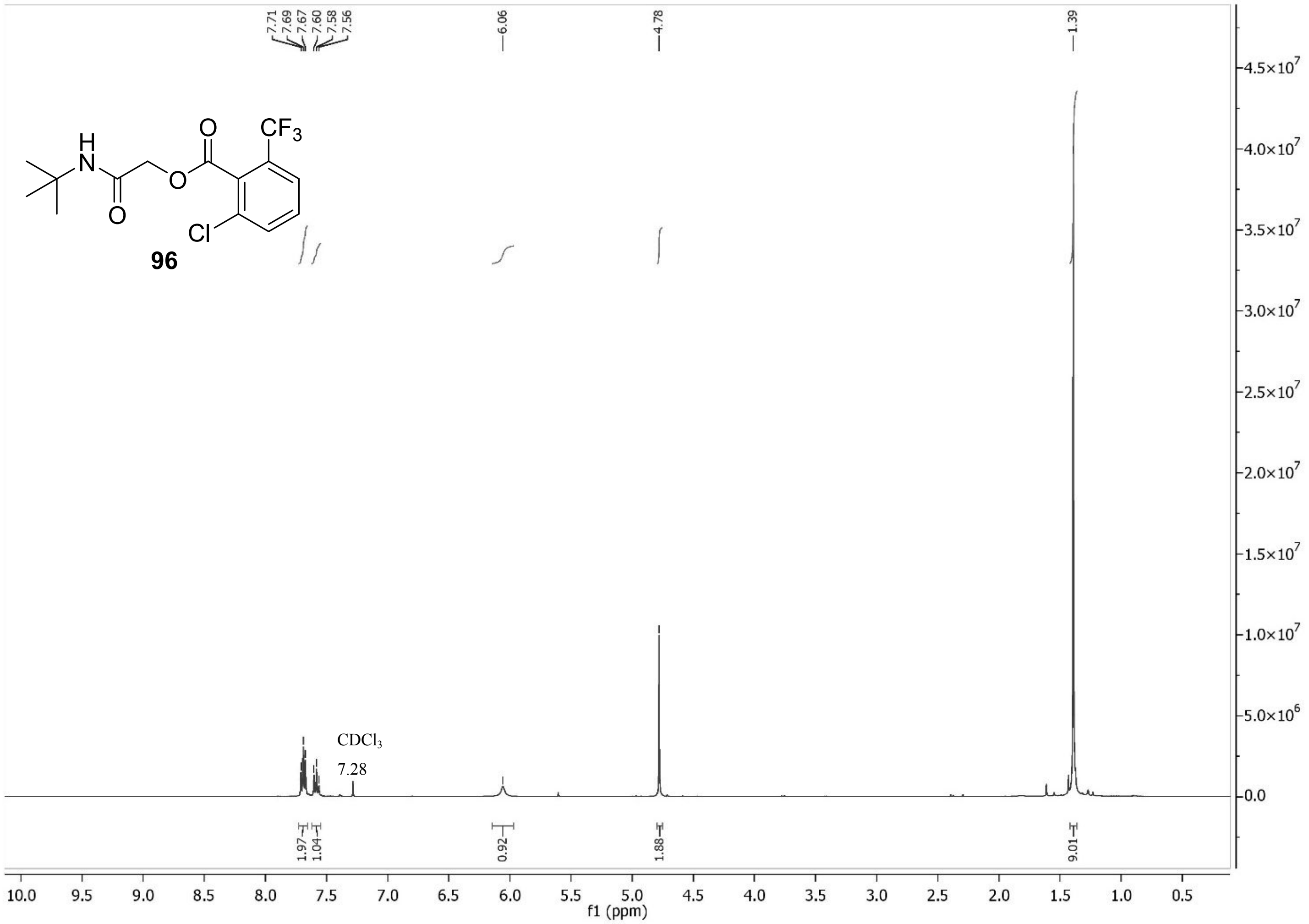

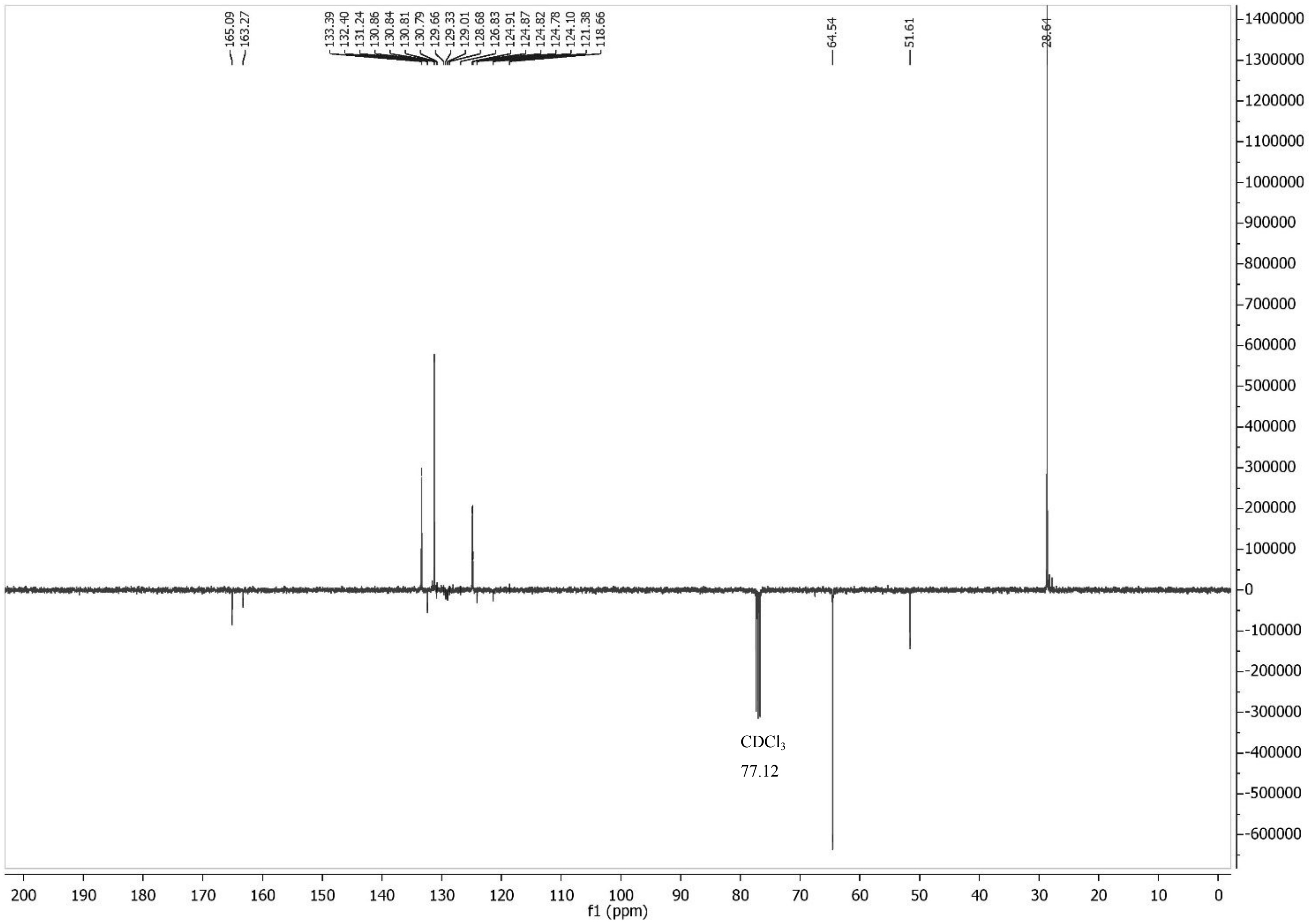

^{1}H and ^{13}C NMR spectra of compound **96**

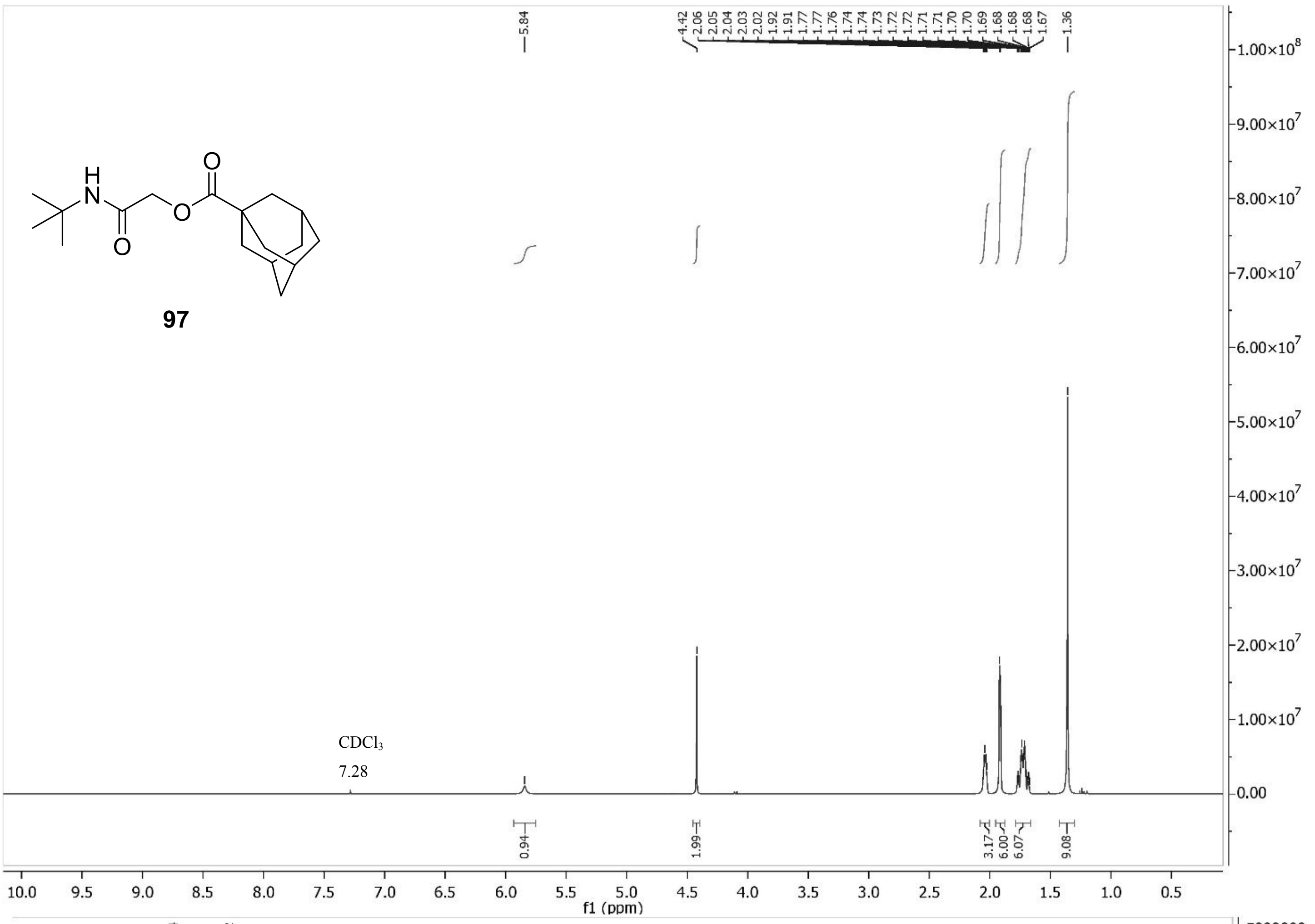

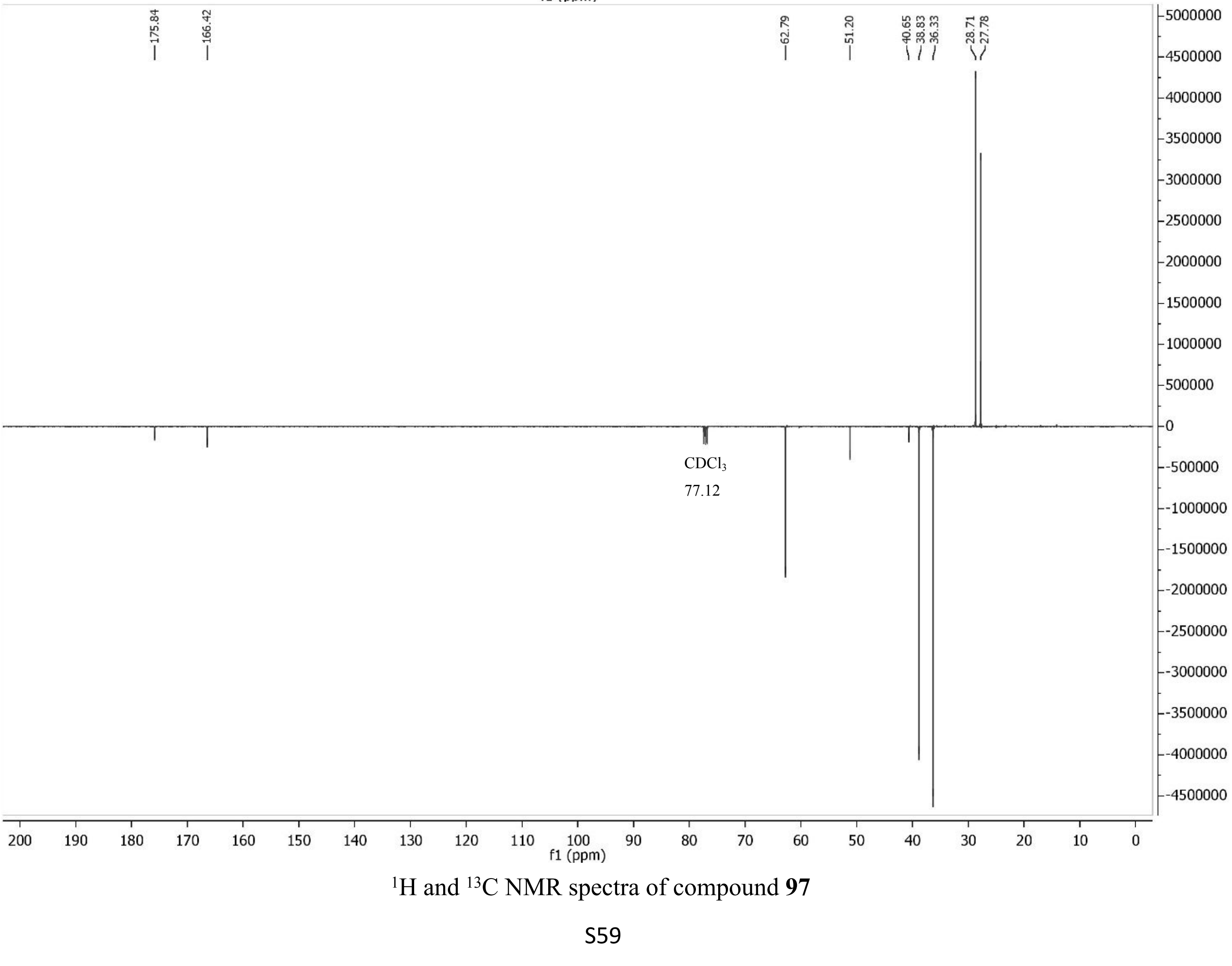

^{1}H and ^{13}C NMR spectra of compound **97**

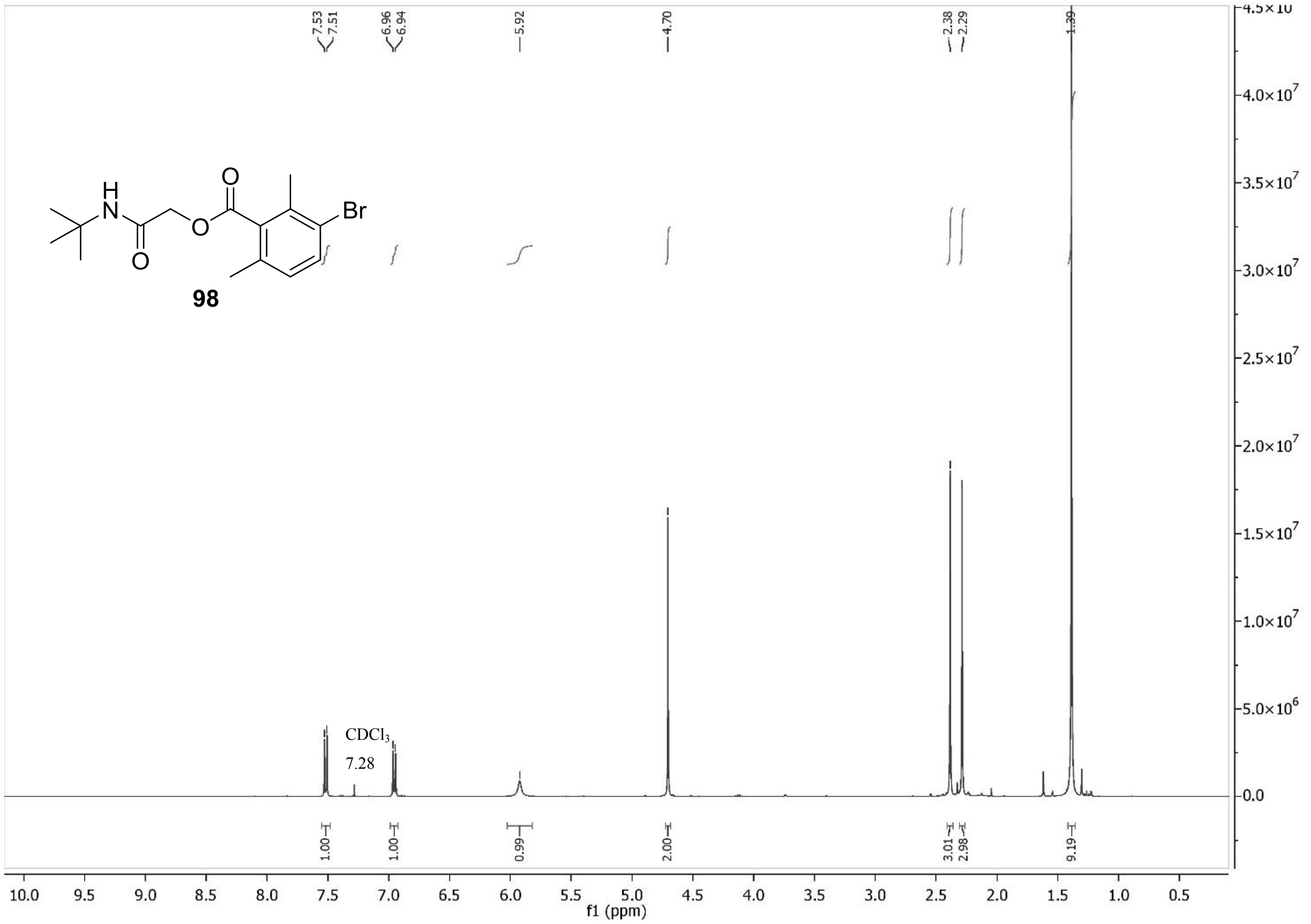

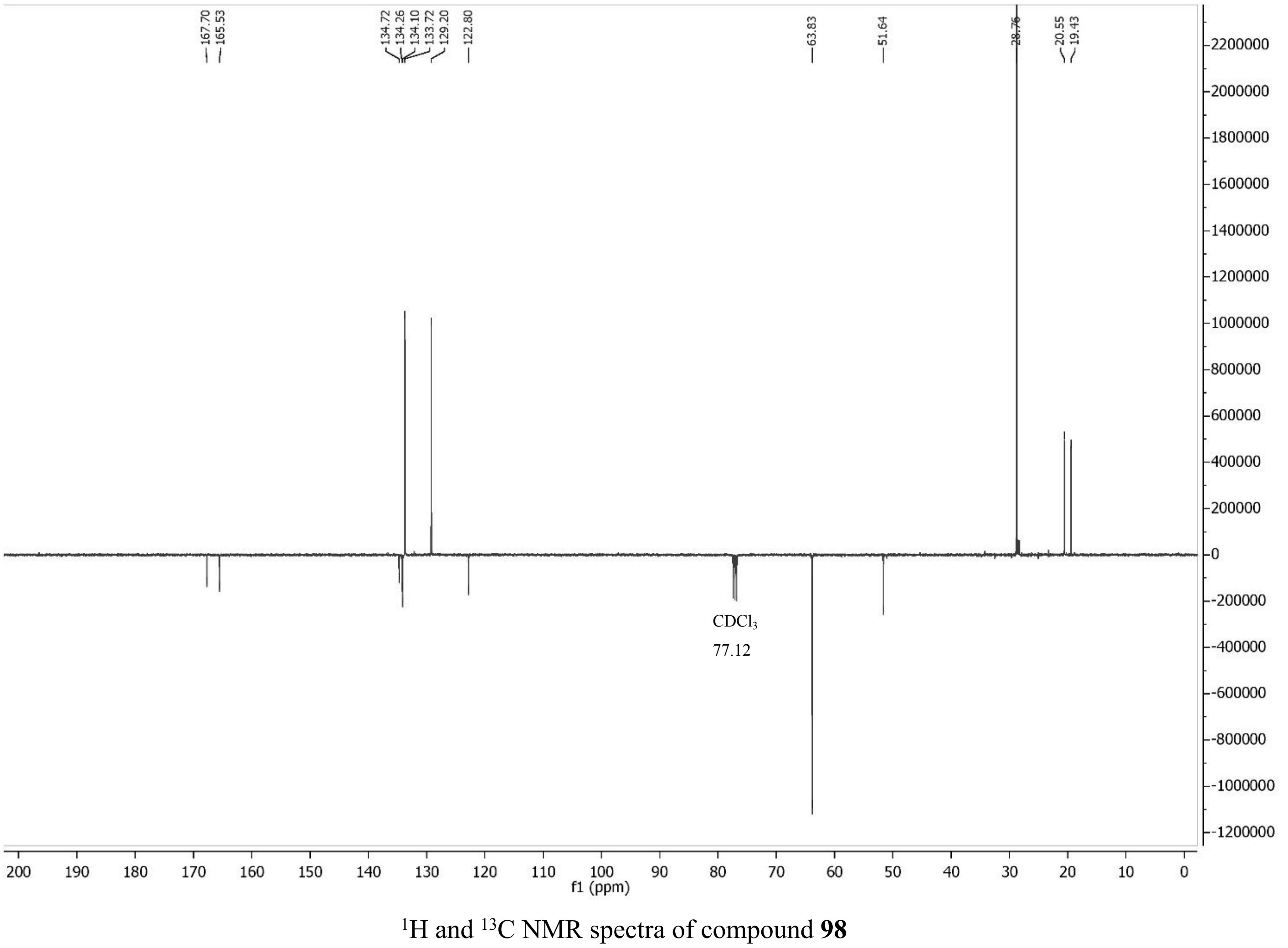

^{1}H and ^{13}C NMR spectra of compound **98**

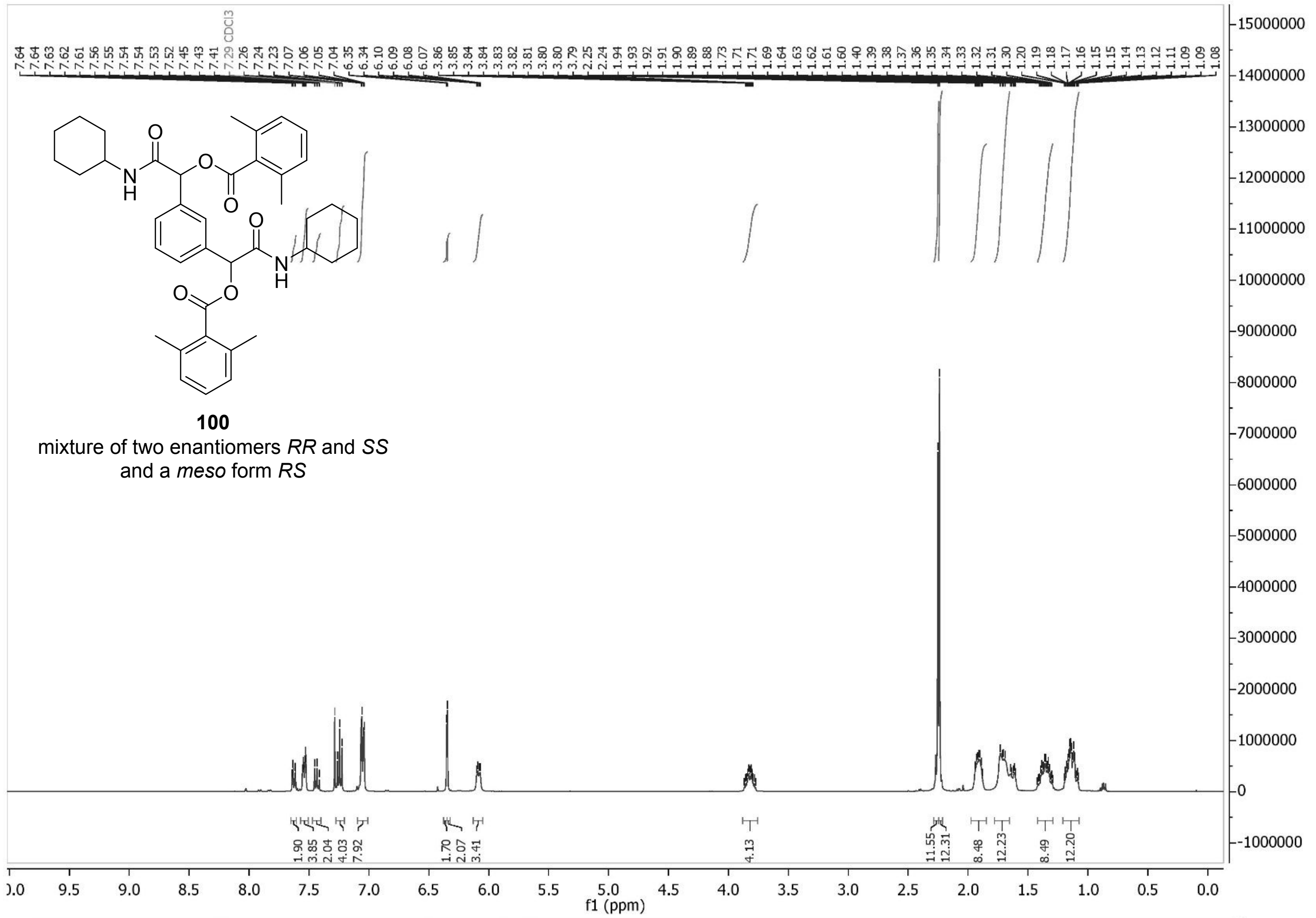

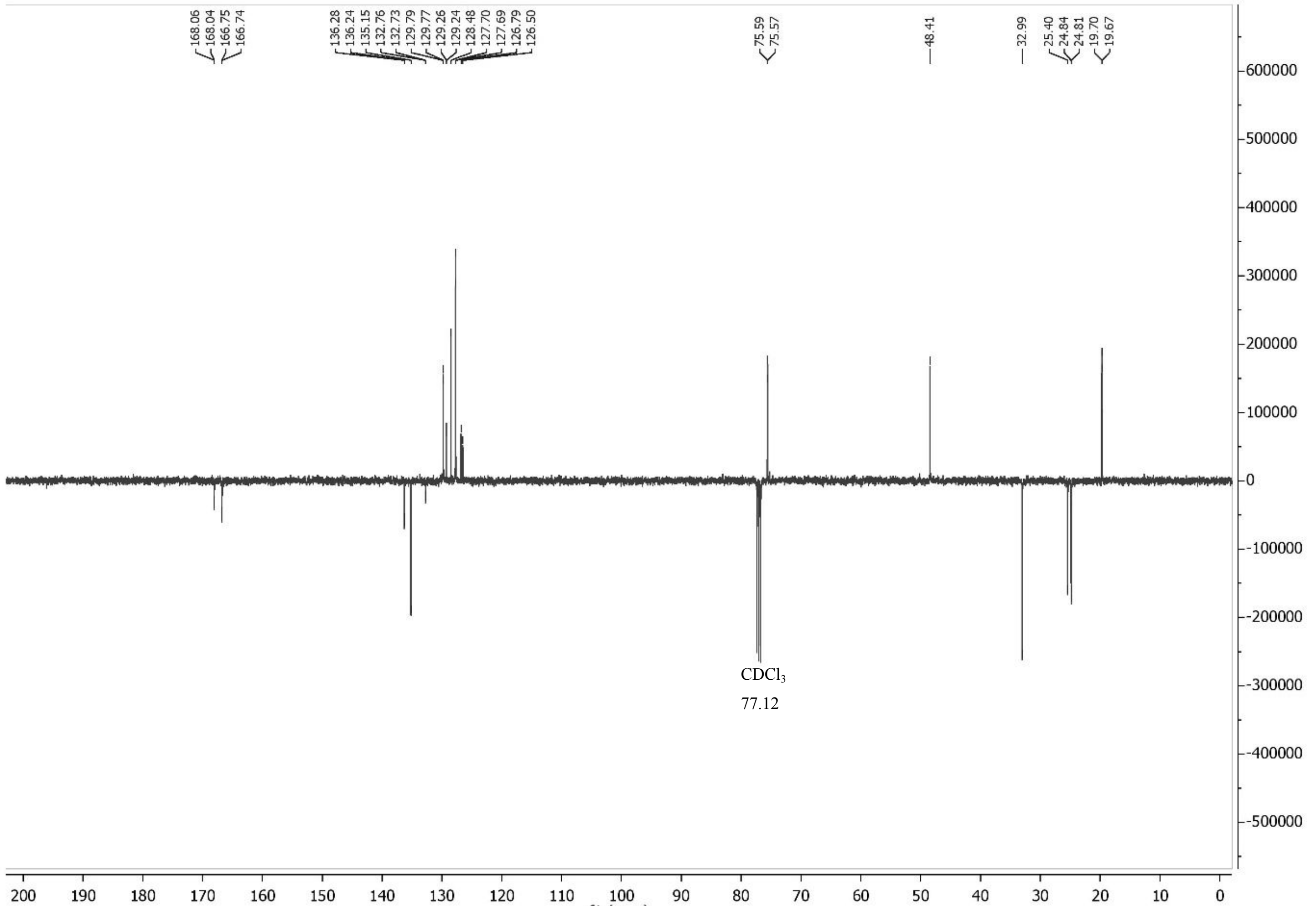

^{1}H and ^{13}C NMR spectra of compound **100**

Purity of selected compounds

HPLC System: Shimadzu LC-10 series, SPD-M10Avp photodiode array detector

Column: Kinetex C18 (150 × 4.6 mm, 5 µm d_p) (Phenomenex).

Mobile Phase Phase A: 0.1% formic acid in deionized and microfiltered water.

Phase B: 0.1% formic acid in acetonitrile HPLC grade.

A:B= 50:50% flow rate= 1.0 mL/min, for all the compounds except **99**, **100**.

A:B= 30:70% flow rate= 1.2 mL/min, for **99**, **100**.

**Compound**	**Area% λ=220 nm**	**Area% λ=280 nm**
**57**	97.6	>99
**58**	>99	95.5
**78**	>99	98.6
**79**	>99	>99
**80**	99.8	97.2
**93**	99.5	>99
**94**	>99	97.6
**96**	>99	96.2
**98**	>99	99.7
**99**	>99	99.6
**100**	>99	97.1

57

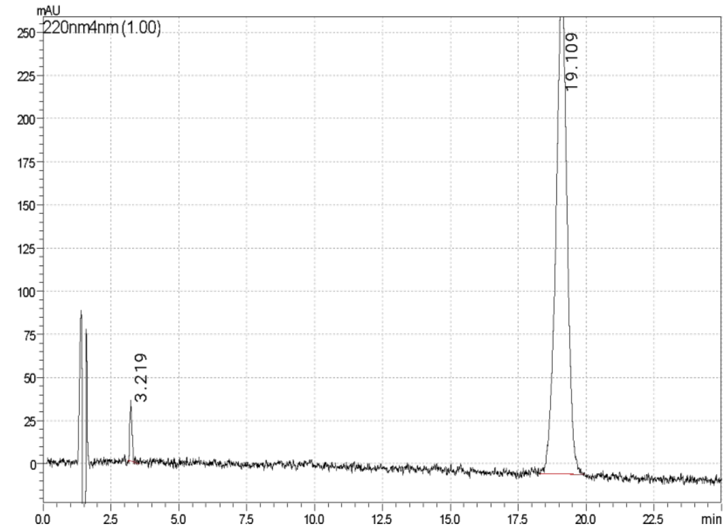
mAU
220nm4nm (1.00)
19.109
3.219
min

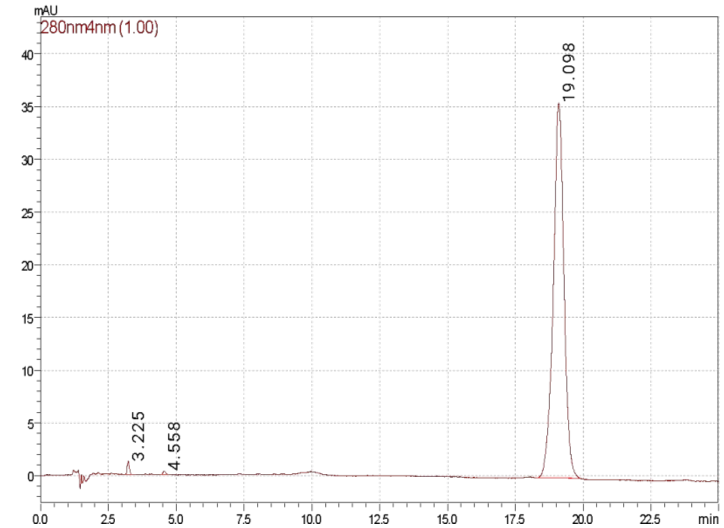
mAU
280nm4nm (1.00)
19.098
3.225
4.558
min

58

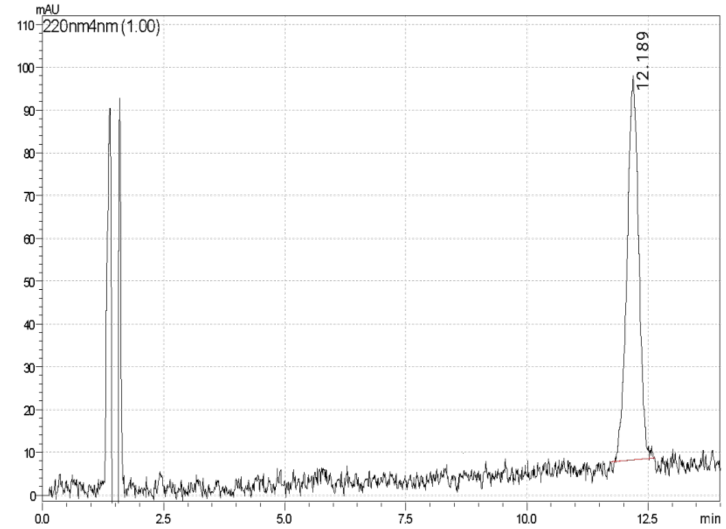
mAU
220nm4nm (1.00)
12.189
min

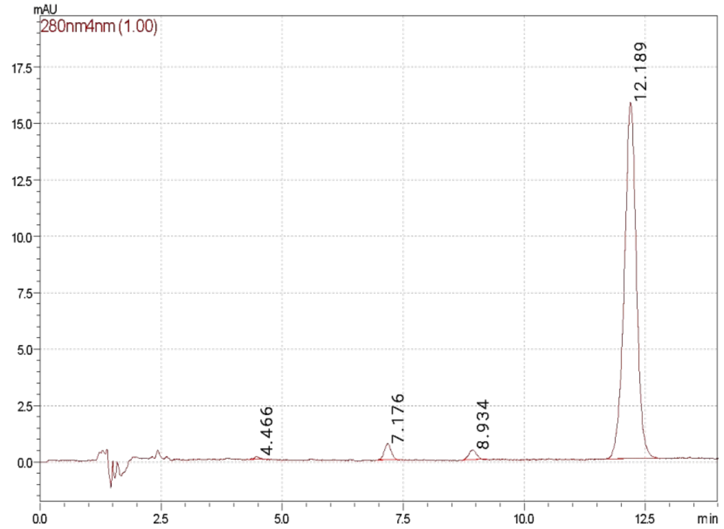
mAU
280nm4nm (1.00)
12.189
4.466
7.176
8.934
min

78

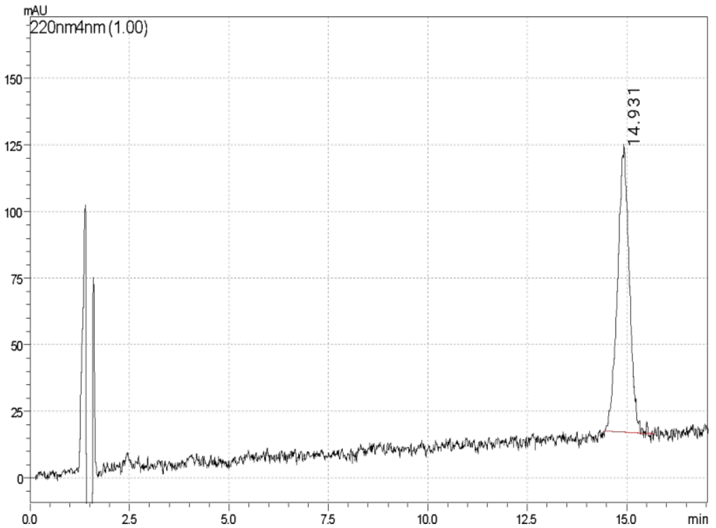
mAU
220nm4nm (1.00)
14.931
min

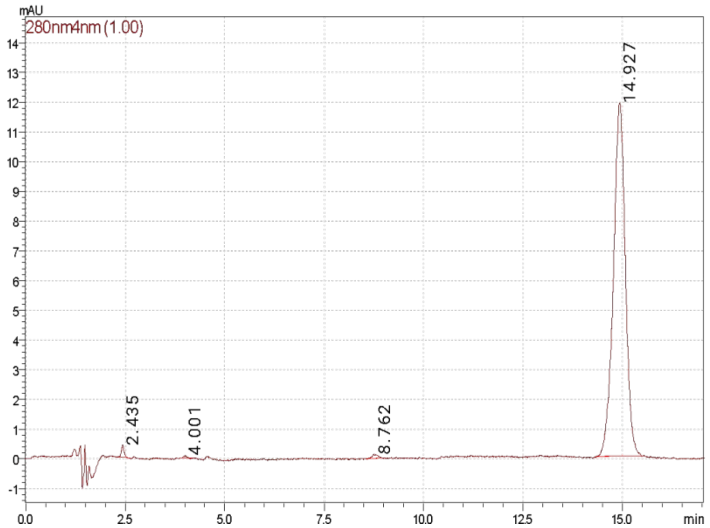
mAU
280nm4nm (1.00)
14.927
2.435
4.001
8.762
min

79

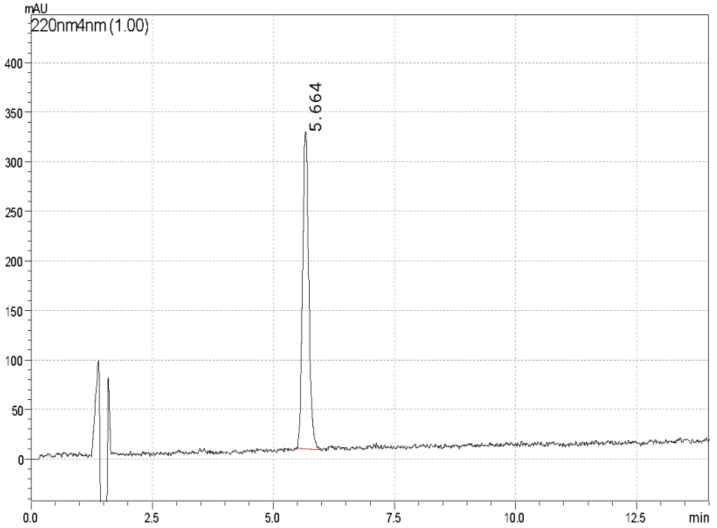
mAU
220nm4nm (1.00)
5.664
min

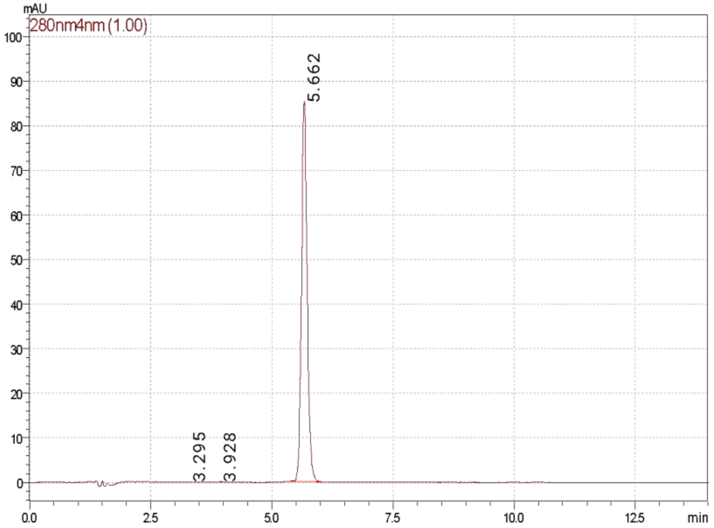
mAU
280nm4nm (1.00)
5.662
3.295
3.928
min

80

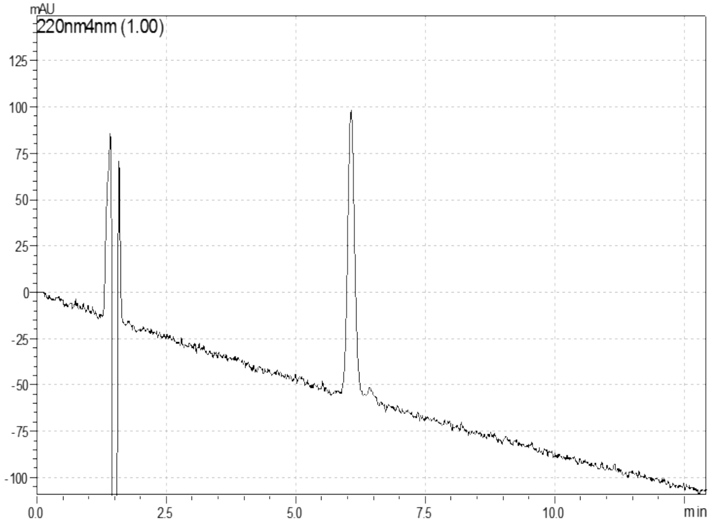
mAU
220nm4nm (1.00)
125
100
75
50
25
0
-25
-50
-75
-100
0.0
2.5
5.0
7.5
10.0
min

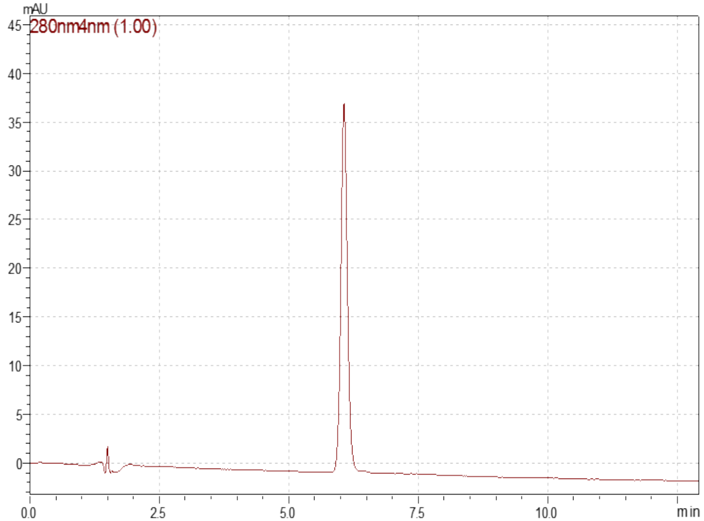
mAU
280nm4nm (1.00)
45
40
35
30
25
20
15
10
5
0
0.0
2.5
5.0
7.5
10.0
min

93

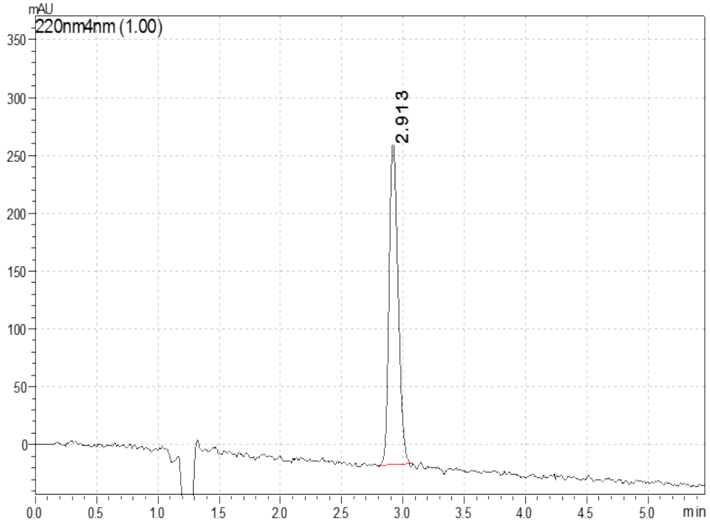
mAU
220nm4nm (1.00)
2.913
350
300
250
200
150
100
50
0
0.0
0.5
1.0
1.5
2.0
2.5
3.0
3.5
4.0
4.5
5.0
min

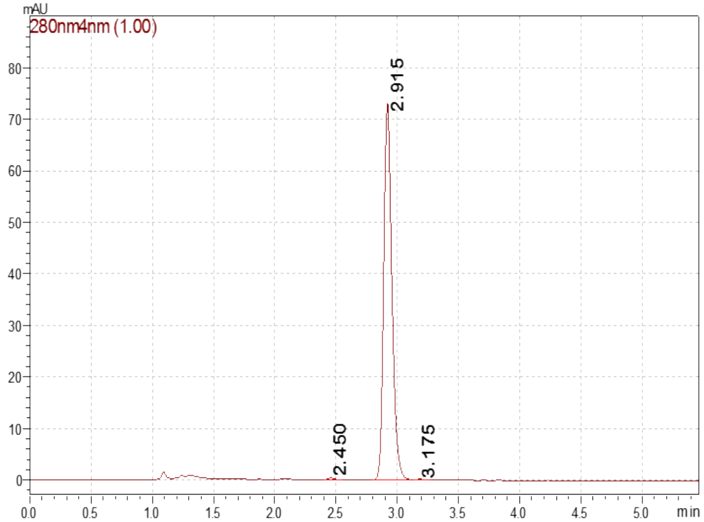
mAU
280nm4nm (1.00)
2.915
2.450
3.175
80
70
60
50
40
30
20
10
0
0.0
0.5
1.0
1.5
2.0
2.5
3.0
3.5
4.0
4.5
5.0
min

94

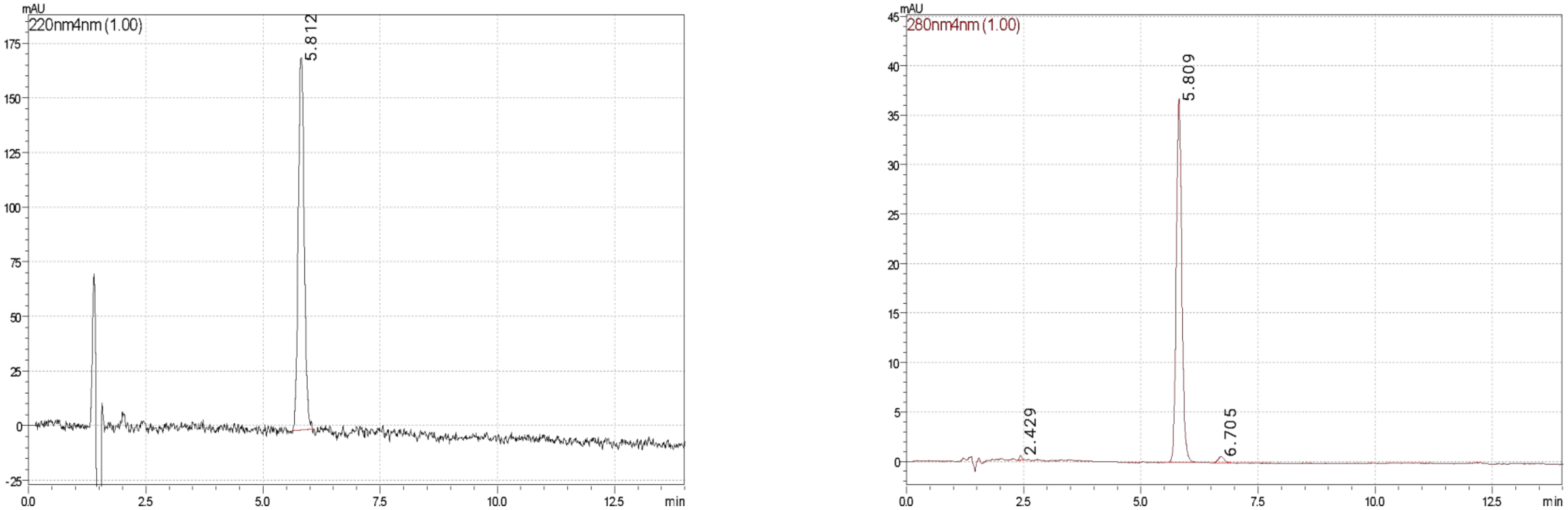
mAU
220nm4nm (1.00)
5.812
min
mAU
280nm4nm (1.00)
5.809
2.429
6.705
min

96

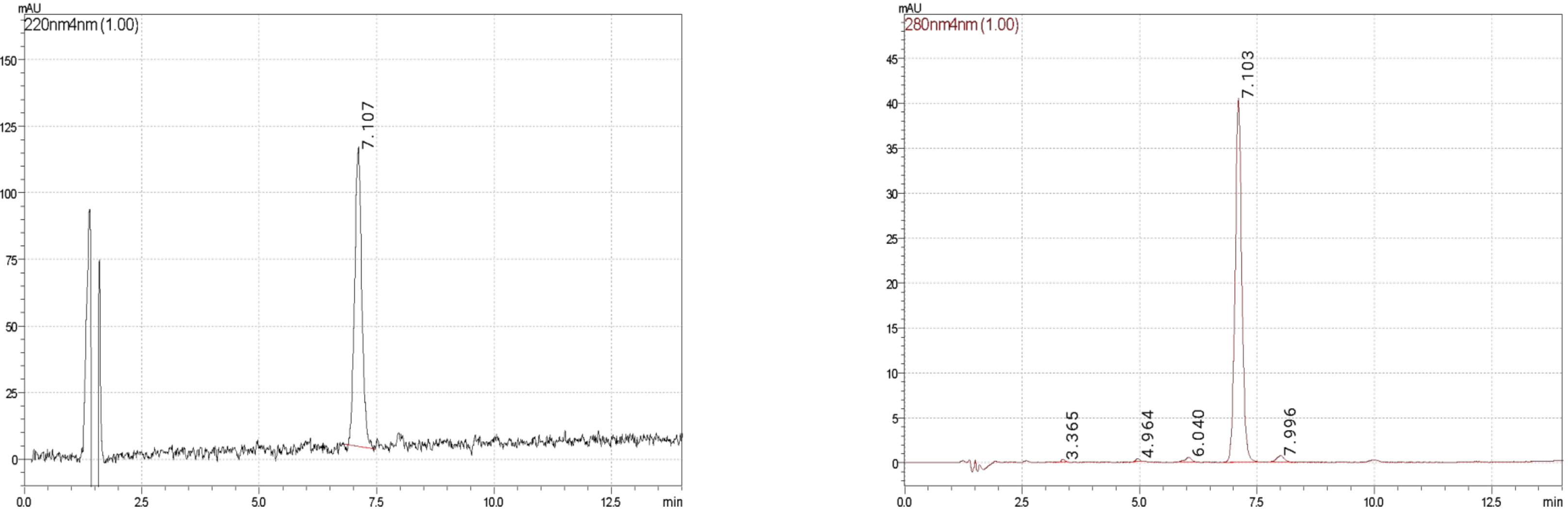
mAU
220nm4nm (1.00)
7.107
min
mAU
280nm4nm (1.00)
7.103
3.365
4.964
6.040
7.996
min

98

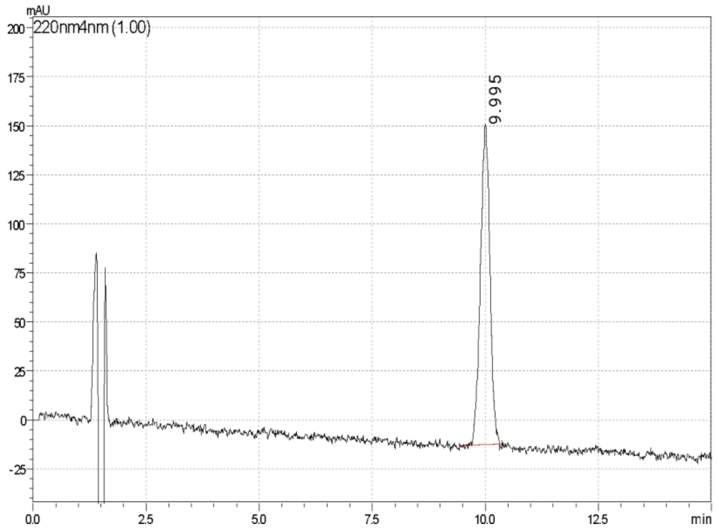

mAU
220nm4nm (1.00)
9.995
min

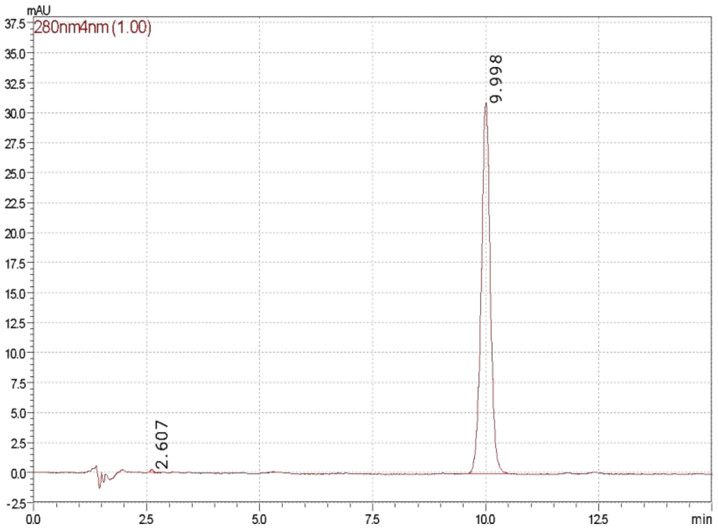

mAU
280nm4nm (1.00)
9.998
2.607
min

99

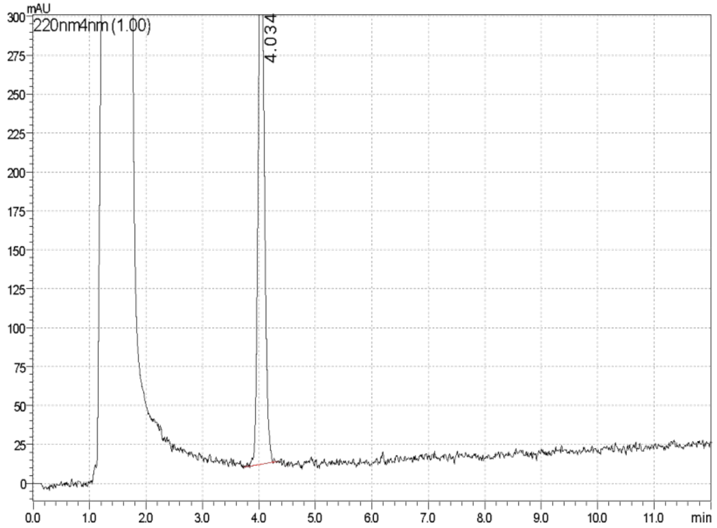

mAU
220nm4nm (1.00)
4.034
min

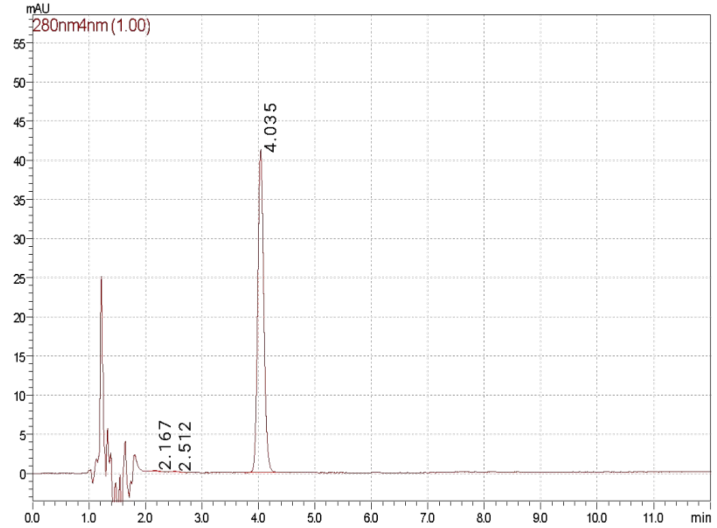

mAU
280nm4nm (1.00)
4.035
2.167
2.512
min

100

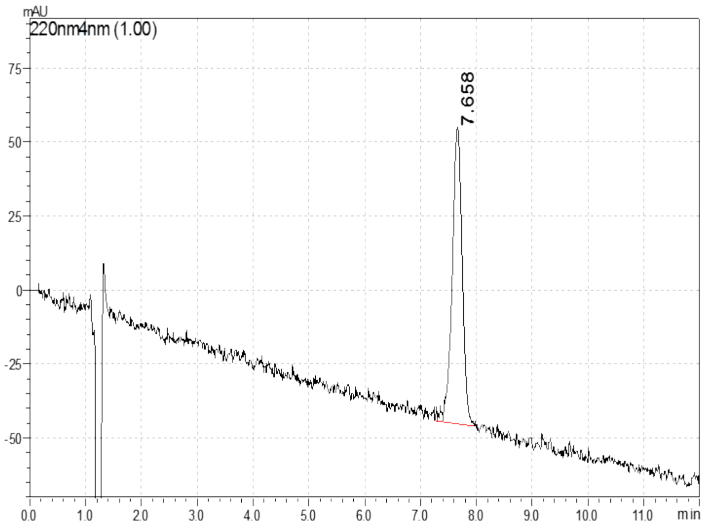
mAU
220nm4nm (1.00)
7.658
75
50
25
0
-25
-50
0.0 1.0 2.0 3.0 4.0 5.0 6.0 7.0 8.0 9.0 10.0 11.0 min

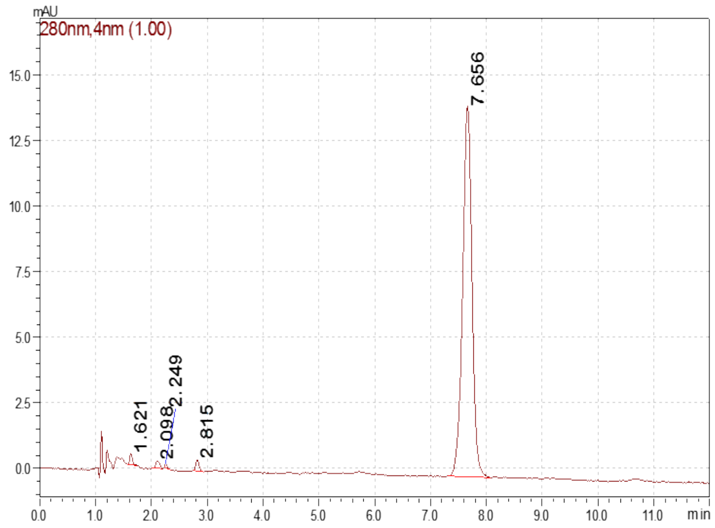
mAU
280nm,4nm (1.00)
7.656
1.621
2.098
2.249
2.815
15.0
12.5
10.0
7.5
5.0
2.5
0.0
0.0 1.0 2.0 3.0 4.0 5.0 6.0 7.0 8.0 9.0 10.0 11.0 min
